# Heterogeneous photocatalytic cyanomethylarylation of alkenes with acetonitrile: synthesis of diverse nitrogenous heterocyclic compounds

**DOI:** 10.3762/bjoc.17.89

**Published:** 2021-05-17

**Authors:** Guanglong Pan, Qian Yang, Wentao Wang, Yurong Tang, Yunfei Cai

**Affiliations:** 1 School of Chemistry and Chemical Engineering, Chongqing University, 174 Shazheng Street, Chongqing 400044, China; 2 Dalian Institute of Chemical Physics, Chinese Academy of Sciences, 457 Zhongshan Road, Dalian 116023, China

**Keywords:** carbon nitride, cyanoalkylarylation, heterocyclic compound, heterogeneous photocatalysis, recyclable

## Abstract

A visible light-mediated heterogeneous photocatalytic cyanomethylarylation of alkenes with acetonitrile has been established using K-modified carbon nitride (CN-K) as a recyclable semiconductor photocatalyst. This protocol, employing readily accessible alkyl *N*-hydroxyphthalimide (NHPI) ester as a radical initiator, allows the efficient construction of a broad array of structural diverse nitrogenous heterocyclic compounds including indolines, oxindoles, isoquinolinones, and isoquinolinediones.

## Introduction

Nitrogenous heterocyclic compounds, such as indolines [1–4], oxindoles [5–7], isoquinolinones [8–10], and isoquinolinediones [11–12], are pivotal structural motifs in numerous pharmaceuticals, agrochemicals, and bioactive natural products. The oxidative cyanomethylarylation of *N*-aryl/benzoyl acrylamide or allylamine involving the key C–H functionalization of readily available acetonitrile is a straightforward and powerful method to access these useful structures [13–17]. Over the last decade, although numerous protocols have been disclosed for the direct cyanomethylarylation of alkenes, most of them rely on the use of transition metals (such as catalytic amounts of Pd, Cu, Fe catalyst or stoichiometric amounts of Ag, Mn salts) and strong oxidants (including PhI(OPiv)_2_, DTBP, and *t*-BuONO) in the presence of various base additives under high reaction temperatures or microwave stimulations [18–26]. Recently, visible light photoredox catalysis has emerged as a powerful and environment-friendly method in organic synthesis by activating organic molecules under mild reaction conditions [27–28]. In this context, the Li and Cai groups independently disclosed a photocatalytic cyanomethylarylation of *N*-aryl/benzoyl acrylamide for the synthesis of oxindoles and isoquinolinediones using diazonium salts and PIFA/1,3,5-trimethoxybenzene as radical initiators, respectively [29–31]. In this case, expensive Ru and 4CzIPN-based homogeneous photocatalysts are used; and the scope remains limited to activated alkenes. Therefore, it is still highly desirable to develop a general, efficient, and practical protocol for the cyanomethylarylation of unactivated alkenes from readily available radical initiators under mild and environmentally benign conditions.

Recently, we demonstrated K-modified carbon nitride (CN-K), a semiconductor material, exhibited a remarkably enhanced photocatalytic activity in the decarboxylative Giese reaction. The effect was due to its K-intercalated poly(heptazine)-based structure existing as small lamellar nanocrystallites, thus leading to an enhanced optical absorption, improved electron-hole separation and easy dispersion in polar solvents [32]. CN-K, which can be easily prepared by the direct KCl-induced structure remodeling of bulk g-C_3_N_4_ [33–36], showed great potentials for future industrial applications given its low cost, recyclability, and high chemical/thermal stability [37–39]. Meanwhile, CN-K has a band gap of 2.72 eV with suitable valence band and conduction band potentials, which allows its promising use in organic synthesis for oxidation and reduction of various substrates [40–43]. In the last few years, carbon nitride-based heterogeneous photocatalysts have also been utilized for several other radical-initiated synthetic transformations [44–56]. However, to the best of our knowledge, the application of a CN-based photocatalyst for the generation of cyanomethyl radicals from readily available acetonitrile has not been reported yet.

**Scheme 1:**
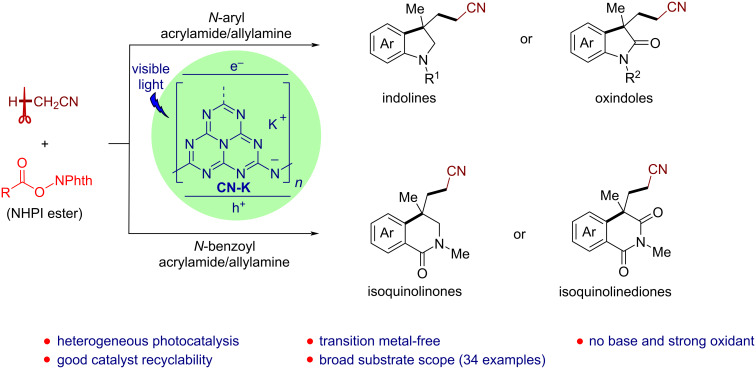
CN-K-Catalyzed cyanomethylarylation of alkenes to access diverse heterocyclic compounds.

Herein, we disclose a CN-K-catalyzed cyanomethylarylation of both unactivated and activated alkenes with acetonitrile utilizing readily available alkyl *N*-hydroxyphthalimide (NHPI) esters as the radical initiator under mild conditions. A wide range of structurally diverse nitrogenous heterocyclic compounds including indolines, oxindoles, isoquinolinones, and isoquinolinediones have been accessed in high yields. This heterogeneous protocol features base- and transition metal-free, good catalyst recyclability, broad substrate scope, and high functional group tolerance (Scheme 1).

## Results and Discussion

Our initial investigation focused on the CN-K photocatalyzed cascade alkyl radical addition/cyclization reaction of the *N*-arylallylamine **1a** with *tert*-butyl *N*-hydroxyphthalimide (NHPI) ester (**2a**), a classical alkyl radical precursor [57–59], to construct indoline product **4**. Surprisingly, the solvent acetonitrile incorporated indoline **3a** was observed as the major product (21%, Table 1, entry 1). Stimulated by this result, we questioned whether it would be possible to develop a general and efficient CN-K-based heterogeneous protocol for the cyanomethylarylation of *N*-arylallylamines with acetonitrile. Pleasingly, after the systematic evaluation of various NHPI esters, which are easily prepared from readily available carboxylic acids, the yield of the product **3a** could be increased to 75% when using the primary NHPI ester **2d** as a radical initiator. The concentration of the reactant had a significant effect on the reaction. When the reaction was performed under higher concentrations (0.05 M), the target product was obtained in 62% yield after prolonged reaction time (Table 1, entry 5). Traditional g-C_3_N_4_ exhibited a low catalytic activity for this transformation (Table 1, entry 6). Switching from CN-K to a homogeneous organo photocatalyst such as eosin Y and 4CzIPN, led to lower yields of the desired product (Table 1, entries 7 and 8). The expensive Ru/Ir-based metal complexes gave similar results for the transformation (Table 1, entries 9 and 10). In the control experiments, no reaction was observed in the absence of the NHPI ester, CN-K, or light, which unambiguously manifested all of them were requisite for this transformation (Table 1, entries 11–13). In addition, no desired product was detected under air, further indicating a radical-initiated process was involved in the reaction (Table 1, entry 14).

**Table 1:** Optimization of the reaction conditions and control experiments^a^.

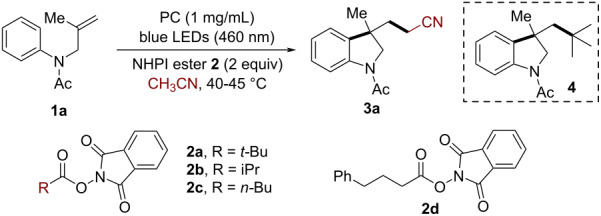			

entry	PC	NHPI ester **2**	yield (%)^b^

1	CN-K	**2a**	21
2	CN–K	**2b**	13
3	CN–K	**2c**	49
4	CN–K	**2d**	75 (72)^c^
5^d^	CN-K	**2d**	62
6	g-C_3_N_4_	**2d**	23
7	eosin Y	**2d**	20
8	4CzIPN	**2d**	18
9^e^	[Ru(bpy)_3_]Cl_2_	**2d**	73
10^e^	fac–Ir(ppy)_3_	**2d**	60
11	CN-K	–	0
12	–	**2d**	0
13^f^	CN-K	**2d**	0
14^g^	CN-K	**2d**	0

^a^Reaction conditions: **1a** (0.1 mmol, 1 equiv), **2** (0.2 mmol, 2 equiv), CH_3_CN (6 mL), PC (1 mg/mL), 2 × 24 W blue LEDs (460 ± 5 nm) without extra heating (at 40–45 °C) for 72 h. ^b^Determined by ^1^H NMR analysis using 1,3,5-trimethoxybenzene as an internal standard. ^c^Data in parentheses are the isolated yields. ^d^Performed with 0.05 M of **1a** for 120 h. ^e^Using 2 mol % of PC. ^f^In the dark. ^g^Under air.

**Scheme 2:**
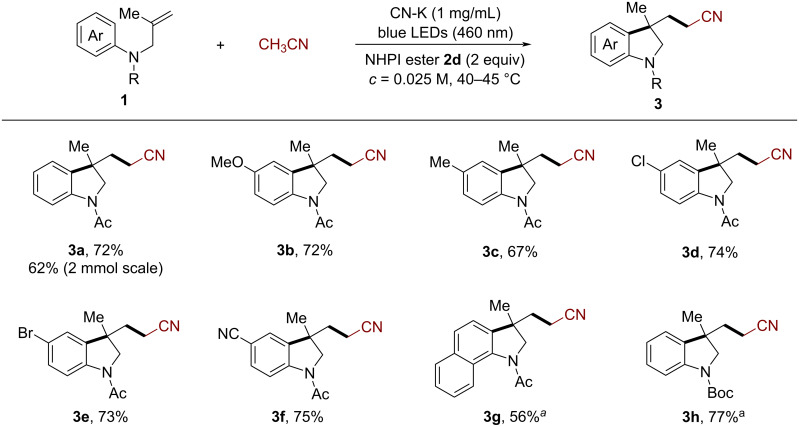
CN-K-catalyzed cyanomethylarylation of *N*-arylallylamines for the synthesis of indolines. Reaction conditions: **1** (0.2 mmol, 1 equiv), **2d** (0.4 mmol, 2 equiv), CH_3_CN (8 mL), CN-K (1 mg/mL), 2 × 24 W blue LEDs (460 ± 5 nm) without extra heating (at 40–45 °C). ^a^Performed with 0.017 M of **1**.

Under the optimal reaction conditions, we first evaluated the scope of *N*-arylallylamines for the synthesis of indolines. As shown in Scheme 2, substrates with various electron-donating groups (such as methyl or methoxy) and electron-withdrawing groups (chloro, bromo, or cyano) at the *para*-position of the aryl ring smoothly reacted with acetonitrile to afford the 5-substituted cyanomethylated indolines **3a**–**f** in good yields (67–75%). When the benzene ring of the substrates was changed to naphthalene, the reaction successfully provided the benzoindoline **3g** in 56% yield. Substrates with an *N*-Boc group also worked well to deliver the corresponding indoline **3h** in 77% yield. Moreover, a scale-up reaction of **1a** (2 mmol) was conducted, affording the corresponding product **3a** (0.28 g) in a yield of 62%.

**Scheme 3:**
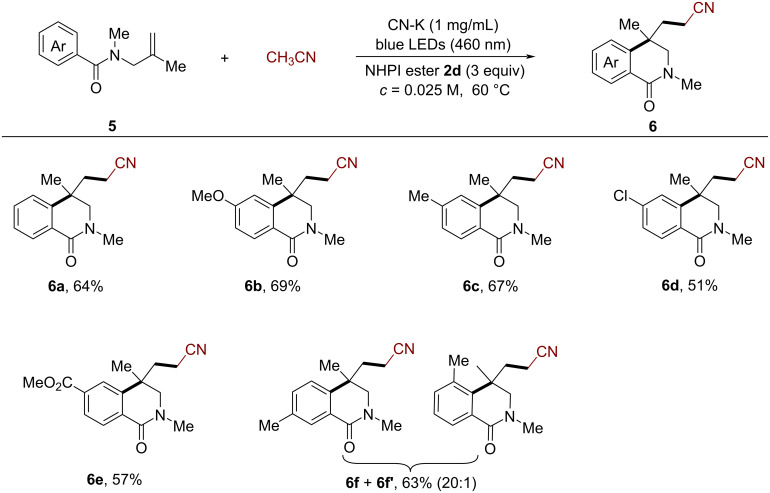
CN-K-catalyzed cyanomethylarylation of *N*-benzoylallylamines for the synthesis of isoquinolinones. Reaction conditions: **5** (0.2 mmol, 1 equiv), **2d** (0.6 mmol, 3 equiv), CH_3_CN (8 mL), CN-K (1 mg/mL), 2 × 24 W blue LEDs (460 ± 5 nm), 60 °C.

Next, we examined the efficiency of the CN-K-catalyzed cyanomethylarylation of *N*-benzoylallylamines for the synthesis of isoquinolinone derivatives. Under similar reaction conditions, various *N-*benzoylallylamines bearing either electron-donating or withdrawing substituents in the *para*-position of the phenyl ring (Scheme 3) were smoothly converted to the corresponding isoquinolinones **6a**–**e** in moderate to good yields (51–69%, Scheme 3). The regioselectivity of this reaction was studied using the *meta*-substituted substrate **5f**. The results indicated that the cyclization occurred at the less crowded positions, and the two regioisomers **6f** and **6f**’ were obtained in a 20:1 ratio.

**Scheme 4:**
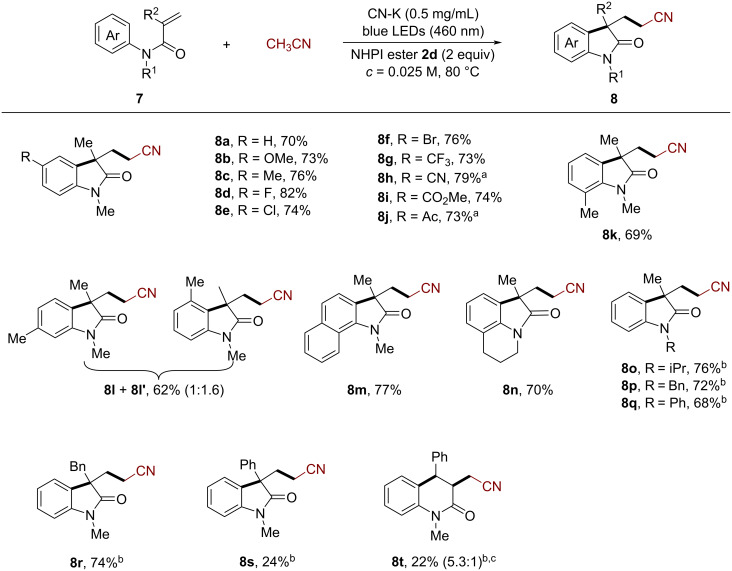
CN-K-catalyzed cyanomethylarylation of *N*-aryl acrylamides for the synthesis of oxindoles. Reaction conditions: **7** (0.2 mmol, 1 equiv), **2d** (0.4 mmol, 2 equiv), CH_3_CN (8 mL), CN-K (0.5 mg/mL), 2 × 24 W blue LEDs (460 ± 5 nm), 80 °C. ^a^Conducted at 100 °C. ^b^Performed with 1 mg/mL of CN-K. ^c^Ratio = *trans*/*cis*.

**Scheme 5:**
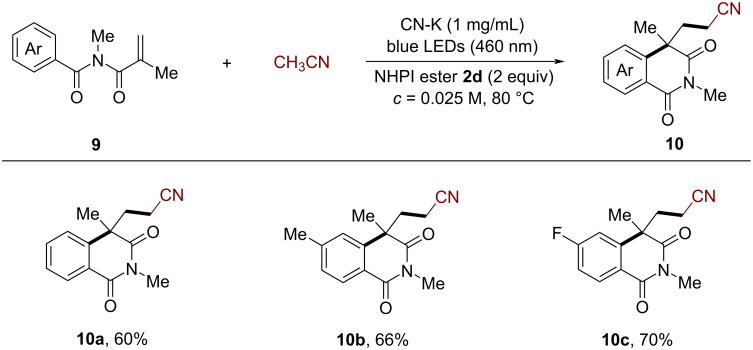
CN-K-catalyzed cyanomethylarylation of *N*-benzoyl acrylamides for the synthesis of isoquinolinediones. Reaction conditions: **9** (0.2 mmol, 1 equiv), **2d** (0.4 mmol, 2 equiv), CH_3_CN (8 mL), CN-K (1 mg/mL), 2 × 24 W blue LEDs (460 ± 5 nm), 80 °C.

The established CN-K-catalyzed cyanomethylarylation protocol is not only effective for the above unactivated alkenes. Also activated alkenes including *N*-aryl and *N*-benzoyl acrylamides can be employed as substrates, allowing for the construction of valuable oxindole and isoquinolinedione derivatives. With respect to the generality of the *N*-aryl acrylamides **7** (Scheme 4), a range of frequently encountered functional groups were well tolerated affording the methoxy (**8b**), methyl (**8c**), the halogenated (**8d**–**f**), trifluromethyl (**8g**), cyano (**8h**), and carbonyl (**8i** and **8j**) substituted products with 70–82% yield, which provide opportunities for further modification. The *ortho*-substituted substrate **7k** gave the corresponding product **8k** in a comparable yield (69%). Also the substrate bearing a *meta*-substituent exhibited good reactivity, delivering the corresponding regioisomers **8l** and **8l’** in 62% with 1:1.6 ratio. Moreover, the naphthalene and tetrahydroisoquinoline-derived acrylamides were also compatible, giving the polycyclic products **8m** and **8n** in 77% and 70%, respectively. Additionally, protecting groups such as isopropyl, benzyl, or phenyl on the nitrogen atom did not considerably affect the yield (**8o**–**8q**, 68–76%). Substrates with benzyl and phenyl groups at the α-position afforded the products **8r** and **8s** in 74% and 24%, respectively. Substrates with a phenyl group at the β-position underwent arylation with the opposite regioselectivity to afford the six-membered product **8t** in 22% yield. Regarding the scope of *N*-benzoyl acrylamides, the electronic property of a substituent on the phenyl ring also had little influence on the reaction, furnishing the desired isoquinolinediones **10a**–**c** in moderate to high yields (Scheme 5, 60–70%).

**Figure 1:**
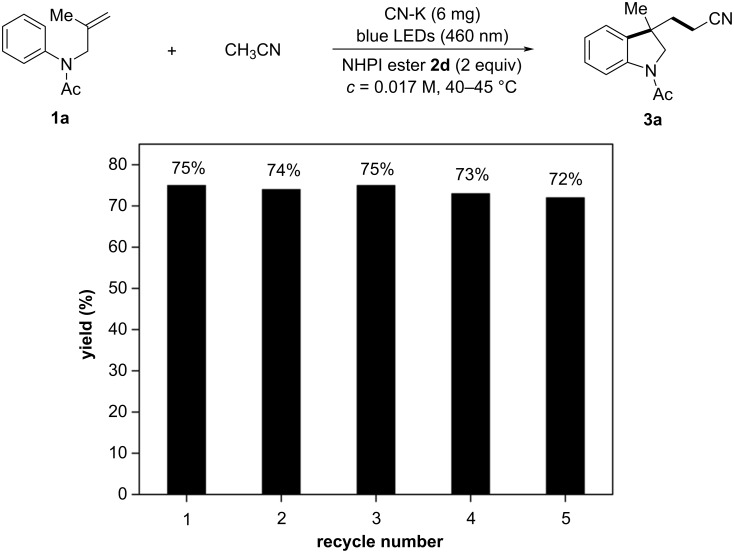
Evaluation of catalyst recycling. Reaction conditions: **1a** (0.1 mmol, 1 equiv), **2d** (0.2 mmol, 2 equiv), CH_3_CN (6 mL), CN-K (1 mg/mL), 2 × 24 W blue LEDs (460 ± 5 nm) without extra heating (at 40–45 °C). After each reaction, the catalyst was recovered by centrifugation and reused in the next cycle.

To illustrate the practicability of this CN-K-based heterogeneous photocatalysis protocol, a recycling procedure was established. The CN-K was recovered via simple centrifugation after the reaction and subsequently reused. As shown in Figure 1, the CN-K catalyst could be recycled at least five times without the loss of photocatalytic activity when applied in the cyanomethylarylation of *N*-arylallylamine **1a** with acetonitrile.

**Scheme 6:**
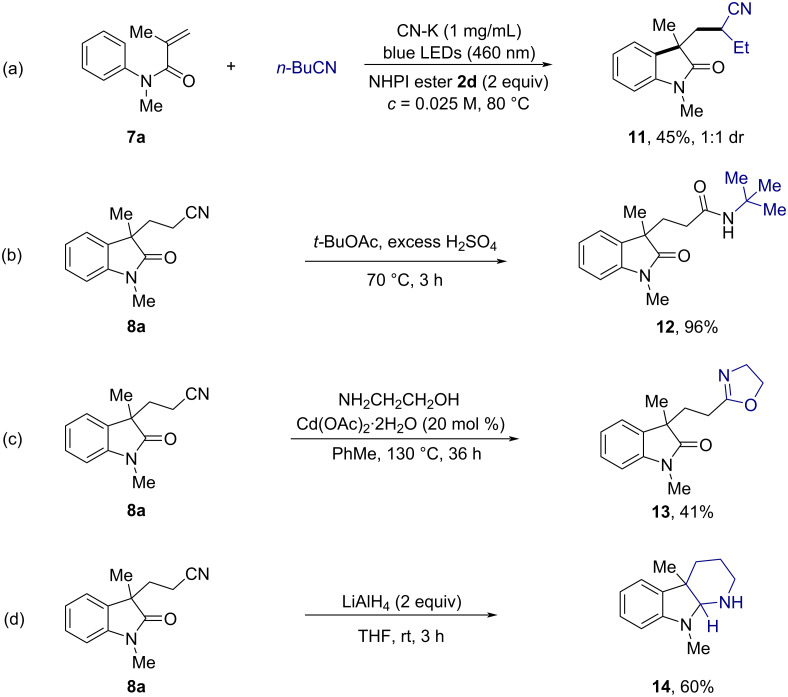
Further survey of reaction scope and derivatization studies of **8a**.

We also utilized this strategy to test *n*-butyl nitrile under the standard conditions. As shown in Scheme 6a, the corresponding oxindole **11** was obtained in 45% yield. The synthetic utility was further demonstrated by a series of successful derivatizations of the cyano-substituted oxindole **8a**. For instance, after the Ritter reaction, **8a** was smoothly converted to *N-tert*-butylated acetamide **12** in 96% yield (Scheme 6b). A modified Witte–Seeliger reaction led to the formation of oxazoline **13** in 41% yield (Scheme 6c). Furthermore, the tricyclic indoline **14**, a structural motif in diverse natural products [4], could be obtained in 60% yield through a reductive cyclization reaction (Scheme 6d).

**Scheme 7:**
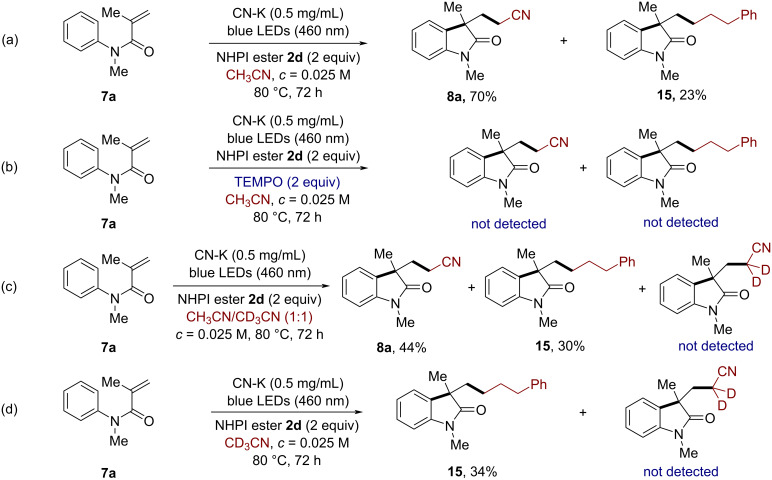
Experiments for the mechanistic study.

To investigate the mechanism of the cyanomethylarylation of alkenes, a series of control experiments was performed (Scheme 7). The cyanomethylarylation reaction of **7a** gave the desired compound **8a** as the major product in 70% yield, along with 23% yield of the byproduct **15**. The latter compound was generated through a cascade alkyl radical addition/cyclization of the NHPI ester **2d** to *N*-aryl acrylamide **7a** (Scheme 7a). When the reaction was conducted in the presence of the radical scavenger 2,2,6,6-tetramethyl-1-piperidinyloxyl (TEMPO), both the desired product **8a** and the byproduct **15** were not observed, which indicated that the reaction proceeds through a radical pathway (Scheme 7b). It is worth mentioning that when **7a** was submitted to the reaction in a 1:1 mixture of CH_3_CN/CD_3_CN as the solvent, the product **8a** was furnished in 44% yield and the corresponding deuterated product was not observed (Scheme 7c). In addition, when **7a** was reacted in CD_3_CN, only the byproduct **15** was obtained in 34% yield (Scheme 7d). These results indicate a large primary isotope effect, which suggest that the C(sp^3^)–H bond cleavage of acetonitrile contributed to the rate-determining step.

**Scheme 8:**
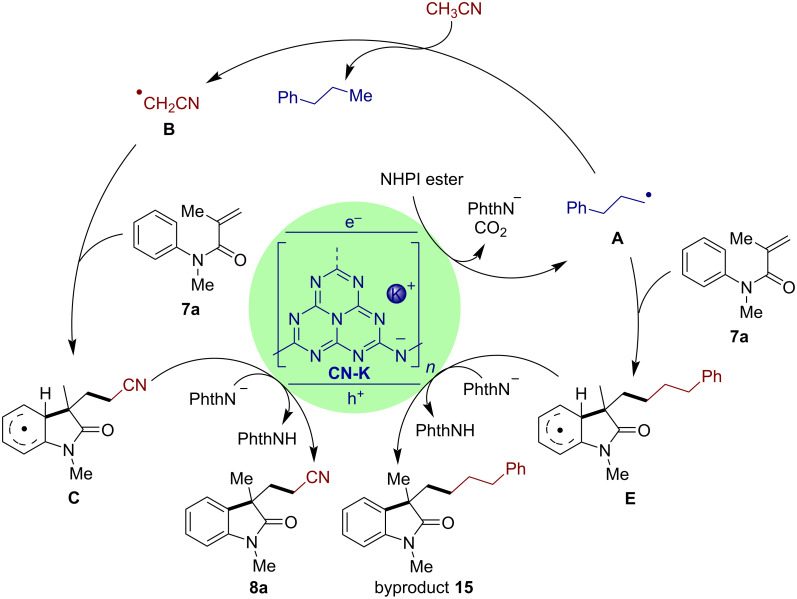
Plausible mechanism of the CN-K-catalyzed cyanomethylarylation of alkenes.

Based on the present results and the literature [18–26,31,60], the reaction pathway is proposed as shown in Scheme 8. As reported in our previous work [32], CN-K has a band gap of 2.72 eV with a conduction band potential of −1.88 V vs SCE, which is thermodynamically favored to induce a single-electron reduction of the NHPI ester (<−1.28 V vs SCE in CH_3_CN) [61] to generate the corresponding alkyl radical **A**. The subsequent hydrogen abstraction by radical **A** yields the key alkyl radical **B**. The addition of radical **B** or **A** to the alkene **7a** following by an intramolecular cyclization provides the radical intermediates **C** or **E**, which are oxidized by the hole of CN-K via SET delivering the product **8a** or byproduct **15** after deprotonation.

## Conclusion

We have demonstrated the application of a heterogeneous CN-K semiconducting photocatalyst in the cyanomethylarylation of alkenes with acetonitrile utilizing a readily available NHPI ester as radical initiator. This transition metal-free protocol tolerates a broad range of both unactivated and activated alkenes including *N*-aryl/benzoylallylamines and acrylamides, providing a facile access to a series of structural diverse indoline, oxindole, isoquinolinone, and isoquinolinedione derivatives with high efficiency. The CN-K catalyst can be easily recovered from the reaction mixture and reused several times, illustrating the practicability of this heterogeneous photocatalysis protocol. Further applying this sustainable and environmentally friendly CN-K heterogeneous photocatalyst to realize other synthetic useful transformations is undergoing.

## Supporting Information

File 1 Full experimental details, compound characterisation, and copies of NMR spectra.
