# Supplementary material for: Heterogeneous photocatalytic cyanomethylarylation of alkenes with acetonitrile: synthesis of diverse nitrogenous heterocyclic compounds
Source: Beilstein J Org Chem. 2021 May 17;17:1171–80. doi: 10.3762/bjoc.17.89 (PMC8144907; doi:10.3762/bjoc.17.89)
Supplement: File 1 — Full experimental details, compound characterisation, and copies of NMR spectra. [file Beilstein_J_Org_Chem-17-1171-s001.pdf]

BEILSTEIN JOURNAL OF ORGANIC CHEMISTRY

Supporting Information

for

Heterogeneous photocatalytic cyanomethylarylation of alkenes with acetonitrile: synthesis of diverse nitrogenous heterocyclic compounds

Guanglong Pan, Qian Yang, Wentao Wang, Yurong Tang and Yunfei Cai

Beilstein J. Org. Chem. **2021,** *17,* 1171–1180. doi:10.3762/bjoc.17.89

Full experimental details, compound characterisation, and copies of NMR spectra

License and Terms: This is a supporting information file under the terms of the Creative Commons Attribution License (https://creativecommons.org/licenses/by/4.0). Please note that the reuse, redistribution and reproduction in particular requires that the author(s) and source are credited and that individual graphics may be subject to special legal provisions.
The license is subject to the *Beilstein Journal of Organic Chemistry* terms and conditions: (https://www.beilstein-journals.org/bjoc/terms)

Table of contents

General Information S2

Synthesis of CN-K S3

General procedure for the CN-K-catalyzed cyanomethylarylation of *N*-aryl allylamines (GP1) S3

General procedure for the CN-K-catalyzed cyanomethylarylation of *N*-benzoyl allylamines (GP2) S7

General procedure for the CN-K-catalyzed cyanomethylarylation of *N*-arylacrylamides (GP3) S10

General procedure for the CN-K-catalyzed cyano-methylarylation of *N*-benzoyl acrylamides (GP4) S17

Scale-up reaction S18

Recycling experiments S19

Synthesis of 2-((1,3-dimethyl-2-oxoindolin-3-yl)methyl)butanenitrile **11** S19

Ritter synthesis of *N*-*tert*-butylpropanamide **12** S20

Witte–Seeliger synthesis of oxazoline **13** S20

Reductive cyclization for the synthesis of pyrroloindoline **14** S21

Synthesis of 1,3-dimethyl-3-(4-phenylbutyl)indolin-2-one **15** S21

^{1}H NMR and ^{13}C NMR spectra of products S23

References S65

General information

All reactions were carried out using oven-dried glassware and magnetic stirring under an inert atmosphere (argon) unless otherwise stated. All chemicals were obtained from commercial supplier and were used without further purification unless otherwise stated. All solvents were dried and distilled under argon prior to use. Solvents for chromatography were of technical grade and distilled prior to use. Analytical thin layer chromatography was carried out using silica gel GF254, visualized under UV light (at 254 nm). Analytical thin layer chromatography was carried out using silica gel GF254. ^{1}H NMR spectra were recorded at 500 MHz, and ^{13}C NMR at 126 MHz NMR spectrometer. All the NMR spectra were processed in MestReNova software. Chemical shifts are quoted in parts per million (ppm) relative to the residual solvent peak for $CDCl_3$. The following abbreviations have been used: δ (chemical shift), *J* (coupling constant expressed in hertz), app. (apparent), br. (broad), s (singlet), d (doublet), dd (doublet of doublets), t (triplet), q (quartet) and m (multiplet). High resolution mass spectra were acquired on a Bruker Solarix 7.0 T FT-ICR mass spectrometer (ESI). The light-promoted reactions were carried out by using standard blue LEDs with 28 blue LED beads (EPISTAR, 1 W LED beads and wavelength 460 ± 5 nm, in Figure S1A). The distance from the blue LEDs to the reaction tube was 4 cm (18.9 mW/cm^2 determined by a power meter). All of the NHPI esters **2**[1], *N*-aryl allylamines **1**[2,3], *N*-benzoyl allylamines **5**[4,5], *N*-aryl acrylamides **7**[6,7], *N*-benzoyl acrylamides **9**[8] were prepared according to literature methods.

Synthesis of CN-K[9]

CN-K was prepared by a two-step polymerization. In the first place, g-C_3N_4 was synthesized by polymerization of melamine (10 g) at 550 °C with a heating rate of 2.3 °C/min under air atmosphere for 4 h. After cooling to room temperature, the product g-C_3N_4 (7 g) was obtained. Then, g-C_3N_4 (1 g) was thoroughly grinded with 10 g of KCl. The mixture was heated to 550 °C at a rate of 2.3 °C/min and kept at this temperature for 4 h under an argon flow (90 mL/min). After cooling to room temperature, the solid mixture was washed with DI water (3 × 9 mL) to remove the metal salts. The final product CN-K (0.85 g) was obtained after drying at 60 °C under vacuum for overnight.

General procedure for the CN-K-catalyzed Cyanomethylarylation of *N*-aryl allylamines (GP1)

To a 30 mL oven-dried sealed tube equipped with a magnetic stirring bar was added **1** (0.2 mmol, 1.0 equiv), **2d** (0.4 mmol, 2.0 equiv), and CN-K (8 mg). The tube was capped with a rubber septum, evacuated, and backfilled with argon. Then, degassed CH_3CN (8 mL) was added via syringe. The sealed tube was irradiated by two blue LEDs (460 ± 5 nm) and the reaction mixture was stirred without extra heating (at 40–45 °C) for the indicated time (see Figure S1A). After complete consumption of the starting material (followed by TLC), the reaction mixture was concentrated under reduced pressure to evaporate the solvent, and the crude residue was purified by silica gel column chromatography to obtain the product **3**. For comparison, two 34 W Kessil PR lamps (75% power) were used as alternative light sources (Figure S1B), and a similar yield of **3a** (0.1 mmol, 16.5 mg, 72% yield) was obtained.

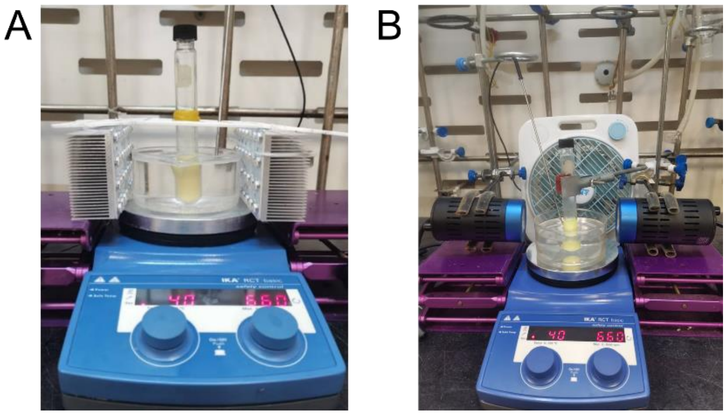

Figure S1 Experimental setup. (A) The photoreactions were performed using two 24 W blue LEDs (460±5 nm). (B) The photoreactions were performed using two 34W Kessil PR lamps (456 nm) made in Taiwan.

3-(1-Acetyl-3-methylindolin-3-yl)propanenitrile (3a)[10]

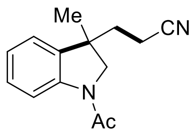

3a

According to **GP1**, workup and flash column chromatography (petroleum ether/ethyl acetate: 1/1) gave product **3a** (32.9 mg, 72% yield, 72 h) as a colorless oil. ^{1}H NMR (500 MHz, $CDCl_3$): δ 8.21 (d, *J* = 7.8 Hz, 1H), 7.27 (s, 1H), 7.09 (s, 2H), 3.98 (d, *J* = 10.6 Hz, 1H), 3.78 (d, *J* = 10.4 Hz, 1H), 2.25 (s, 3H), 2.22 – 2.10 (m, 2H), 2.09 – 1.87 (m, 2H), 1.44 (s, 3H). ^{13}C NMR (126 MHz, $CDCl_3$): δ 168.7, 142.4, 136.0, 128.8, 124.2, 122.2, 119.5, 117.2, 60.6, 43.2, 37.0, 26.6, 24.3, 12.9.

3-(1-Acetyl-5-methoxy-3-methylindolin-3-yl)propanenitrile (3b)[11]

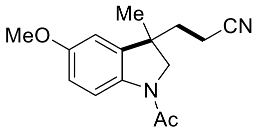

3b

According to **GP1**, workup and flash column chromatography (petroleum ether/ethyl acetate: 1/1) gave product **3b** (37.2 mg, 72% yield, 72 h) as a colorless oil. ^{1}H NMR (500 MHz, $CDCl_3$): δ 8.14 (d, *J* = 8.7 Hz, 1H), 6.78 (d, *J* = 8.7 Hz, 1H), 6.63 (s, 1H), 3.97 (d, *J* = 10.6 Hz, 1H), 3.80 (s, 4H), 2.22 (s, 3H), 2.20 – 2.08 (m, 2H), 2.06 – 1.90 (m, 2H), 1.43 (s, 3H). ^{13}C NMR (126 MHz, $CDCl_3$): δ 168.0, 156.7, 137.6, 136.1, 119.48, 118.0, 112.8, 108.8, 60.8, 55.7, 43.3, 37.0, 26.5, 24.0, 12.9.

3-(1-Acetyl-3,5-dimethylindolin-3-yl)propanenitrile (3c)[11]

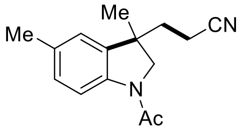

3c

According to **GP1**, workup and flash column chromatography (petroleum ether/ethyl acetate: 1/1) gave product **3c** (32.5 mg, 67% yield, 72 h) as a white solid. ^{1}H NMR (500 MHz, $CDCl_3$): δ 8.08 (d, *J* = 8.1 Hz, 1H), 7.06 (d, *J* = 7.8 Hz, 1H), 6.87 (s, 1H), 3.95 (d, *J* = 10.6 Hz, 1H), 3.76 (d, *J* = 10.5 Hz, 1H), 2.33 (s, 3H), 2.23 (s, 3H), 2.19 – 2.10 (m, 2H), 2.05 – 1.90 (m, 2H), 1.42 (s, 3H). ^{13}C NMR (126 MHz, $CDCl_3$): δ 168.4, 140.1 136.0, 133.9, 129.3, 122.7, 119.6, 117.0, 60.7, 43.2, 37.1, 26.6, 24.2, 21.2, 12.9.

3-(1-Acetyl-5-chloro-3-methylindolin-3-yl)propanenitrile (3d)[11]

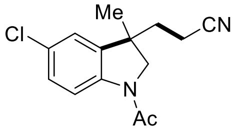

3d

According to **GP1**, workup and flash column chromatography (petroleum ether/ethyl acetate: 1/1) gave product 3d (38.9 mg, 74% yield, 72 h) as a colorless oil. ^{1}H NMR (500 MHz, $CDCl_3$): δ 8.16 (d, *J* = 8.4 Hz, 1H), 7.22 (d, *J* = 8.5 Hz, 1H), 7.04 (s, 1H), 4.03 (d, *J* = 10.5 Hz, 1H), 3.80 (d, *J* = 10.6 Hz, 1H), 2.24 (s, 3H), 2.22 – 2.16 (m, 2H), 2.09 – 1.92 (m, 2H), 1.44 (s, 3H). ^{13}C NMR (126 MHz, $CDCl_3$): δ 168.7, 141.0, 138.0, 129.1, 128.8, 122.5, 119.2, 118.3, 60.6, 43.3, 36.9, 26.5, 24.2, 12.9.

3-(1-Acetyl-5-bromo-3-methylindolin-3-yl)propanenitrile (3e)[11]

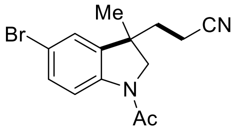

3e

According to **GP1**, workup and flash column chromatography (petroleum ether/ethyl acetate: 1/1) gave product **3e** (44.8 mg, 73% yield, 72 h) as a colorless oil. ^{1}H NMR (500 MHz, $CDCl_3$): δ 8.10 (d, *J* = 8.5 Hz, 1H), 7.36 (d, *J* = 8.4 Hz, 1H), 7.18 (s, 1H), 4.02 (d, *J* = 10.6 Hz, 1H), 3.79 (d, *J* = 10.6 Hz, 1H), 2.24 (s, 3H), 2.21 – 2.15 (m, 2H), 2.09 – 1.92 (m, 2H), 1.43 (s, 1H). ^{13}C NMR (126 MHz, $CDCl_3$): δ 168.8, 141.5, 138.4, 131.7, 125.4, 119.2, 118.7, 116.5, 60.6, 43.3, 36.9, 26.6, 24.2, 12.9.

1-Acetyl-3-(2-cyanoethyl)-3-methylindoline-5-carbonitrile (3f)[10]

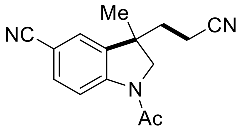

3f

According to **GP1**, workup and flash column chromatography (petroleum ether/ethyl acetate: 1/1) gave product **3f** (38.0 mg, 75% yield, 72 h) as a colorless oil. ^{1}H NMR (500 MHz, $CDCl_3$): δ 8.21 (d, *J* = 7.8 Hz, 1H), 7.27 (s, 1H), 7.09 (s, 2H), 3.98 (d, *J* = 10.6 Hz, 1H), 3.78 (d, *J* = 10.4 Hz, 1H), 2.25 (s, 3H), 2.22 – 2.10 (m, 2H), 2.09 – 1.87 (m, 2H), 1.44 (s, 3H). ^{13}C NMR (126 MHz, $CDCl_3$): δ 169.4, 145.9, 137.5, 133.8, 126.1, 119.0, 118.9, 117.5, 107.0, 60.6, 43.3, 36.6, 26.5, 24.4, 12.9.

3-(1-Acetyl-3-methyl-2,3-dihydro-1*H*-benzo[*g*]indol-3-yl)propanenitrile (3g)[11]

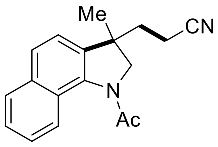

3g

According to **GP1**, workup and flash column chromatography (petroleum ether/ethyl acetate: 3/1) gave product **3g** (31.2 mg, 56% yield, 72 h) as a white solid. ^{1}H NMR (500 MHz, $CDCl_3$): δ 7.78 (d, *J* = 8.1 Hz, 1H), 7.70 (d, *J* = 8.1 Hz, 1H), 7.52 (t, *J* = 7.6 Hz, 1H), 7.47 (t, *J* = 7.7 Hz, 1H), 7.38 (d, *J* = 6.9 Hz, 1H), 7.33 – 7.26 (m, 1H), 4.53 (s, 1H), 3.50 (d, *J* = 12.4 Hz, 1H), 2.58 – 2.42 (m, 1H), 2.40 (s, 3H), 2.38 – 2.25 (m, 1H), 2.13 – 1.87 (m, 2H), 1.45 (s, 3H). ^{13}C NMR (126 MHz, $CDCl_3$): δ 170.1, 134.1, 128.5, 128.4, 127.2, 126.2, 125.6, 125.2, 123.7, 122.3, 119.8, 119.4, 50.4, 39.4, 35.0, 23.6, 22.9, 12.9.

***tert*-Butyl 3-(2-cyanoethyl)-3-methylindoline-1-carboxylate (3h)**[11]

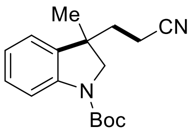

3h

According to GP1, workup and flash column chromatography (petroleum ether/ethyl acetate: 6/1) gave product 3h (44.1 mg, 77% yield, 72 h) as a white solid. ^{1}H NMR (500 MHz, $CDCl_3$): δ 8.01 – 7.35 (m, 1H), 7.22 (t, *J* = 7.2 Hz, 1H), 7.06 (d, *J* = 6.9 Hz, 1H), 7.00 (t, *J* = 7.2 Hz, 1H), 3.84 (d, *J* = 11.3 Hz, 1H), 3.66 (d, *J* = 11.3 Hz, 1H), 2.30 – 2.15 (m, 1H), 2.14 – 1.95 (m, 3H), 1.57 (s, 9H), 1.40 (s, 3H). ^{13}C NMR (126 MHz, $CDCl_3$): δ 152.2, 141.6, 136.0, 128.6, 122.7, 122.4, 119.5, 115.0, 81.8, 59.3, 44.2, 37.3, 28.5, 26.6, 12.9.

General procedure for the CN-K-catalyzed cyanomethylarylation of *N*-benzoyl allylamines (GP2)

To a 30 mL oven-dried sealed tube equipped with a magnetic stirring bar was added **2d** (0.6 mmol, 3.0 equiv) and CN-K (8 mg). It was capped with a rubber septum, evacuated, and backfilled with argon. Then, degassed CH_3CN (8 mL) and **5** (0.2 mmol, 1.0 equiv) were added via syringe. The sealed tube was irradiated by two blue LEDs (460 ± 5 nm) and the reaction mixture was stirred at 60 °C for the indicated time (see Figure S1A). After complete consumption of the starting material (followed by TLC), the reaction mixture was concentrated under reduced pressure to evaporate the solvent, and the crude residue was purified by silica gel column chromatography to obtain the product **6**.

3-(2,4-Dimethyl-1-oxo-1,2,3,4-tetrahydroisoquinolin-4-yl)propane-nitrile (6a)[10]

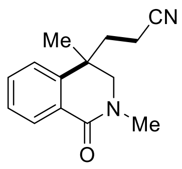

6a

According to **GP2**, workup and flash column chromatography (petroleum ether/ethyl acetate: 1/1) gave product **6a** (29.2 mg, 64% yield, 72 h) as a colorless oil. ^{1}H NMR (500 MHz, $CDCl_3$): δ 8.15 (d, *J* = 7.5 Hz, 1H), 7.51 (t, *J* = 7.2 Hz, 1H), 7.40 (t, *J* = 7.4 Hz, 1H), 7.23 (d, *J* = 7.5 Hz, 1H), 3.58 (d, *J* = 12.8 Hz, 1H), 3.23 (d, *J* = 12.8 Hz, 1H), 3.18 (s, 3H), 2.32 – 2.07 (m, 3H), 2.01 – 1.90 (m, 1H), 1.42 (s, 3H). ^{13}C NMR (126 MHz, $CDCl_3$): δ 164.2, 142.5, 132.1, 129.1, 128.4, 127.7, 124.2, 119.5, 58.7, 36.9, 35.3, 34.9, 22.6, 12.9.

3-(6-Methoxy-2,4-dimethyl-1-oxo-1,2,3,4-tetrahydroisoquinolin-4-yl)propanenitrile (6b)[10]

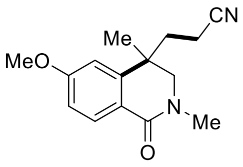

6b

According to **GP2**, workup and flash column chromatography (petroleum ether/ethyl acetate: 1/1) gave product **6b** (35.6 mg, 69% yield, 72 h) as a brown oil. ^{1}H NMR (500 MHz, $CDCl_3$): δ 8.10 (d, J = 8.6 Hz, 1H), 6.89 (d, J = 8.6 Hz, 1H), 6.70 (s, 1H), 3.87 (s, 3H), 3.55 (d, J = 12.6 Hz, 1H), 3.19 (d, J = 12.7 Hz, 1H), 3.15 (s, 3H), 2.28 – 2.03 (m, 3H), 2.01 – 1.87 (m, 1H), 1.39 (s, 3H). ^{13}C NMR (126 MHz, $CDCl_3$): δ 164.2, 162.6, 144.6, 131.4, 121.2, 119.5, 111.9, 110.6, 58.8, 55.5, 37.0, 35.2, 34.9, 22.7, 13.0.

3-(2,4,6-Trimethyl-1-oxo-1,2,3,4-tetrahydroisoquinolin-4-yl)propanenitrile (6c)[10]

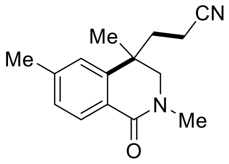

6c

According to **GP2**, workup and flash column chromatography (petroleum ether/ethyl acetate: 1/1) gave product **6c** (32.5 mg, 67% yield, 72 h) as a colorless oil. ^{1}H NMR (500 MHz, $CDCl_3$): δ 8.03 (d, J = 7.8 Hz, 1H), 7.20 (d, J = 7.7 Hz, 1H), 7.00 (s, 1H), 3.55 (d, J = 12.7 Hz, 1H), 3.20 (d, J = 12.7 Hz, 1H), 3.16 (s, 3H), 2.40 (s, 3H), 2.27 – 2.07 (m, 3H), 2.00 – 1.90 (m, 1H), 1.40 (s, 3H). ^{13}C NMR (126 MHz, $CDCl_3$): δ 164.4, 142.6, 142.4, 129.2, 128.5, 125.8, 124.7, 119.6, 58.8, 36.9, 35.2, 35.0, 22.7, 21.9, 13.0.

3-(6-Chloro-2,4-dimethyl-1-oxo-1,2,3,4-tetrahydroisoquinolin-4-yl)propanenitrile (6d)[10]

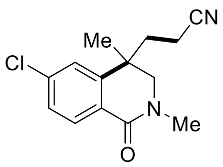

6d

According to **GP2**, workup and flash column chromatography (petroleum ether/ethyl acetate: 1/1) gave product **6d** (26.8 mg, 51% yield, 72 h) as a colorless oil. ^{1}H NMR (500 MHz, $CDCl_3$): δ 8.09 (d, J = 8.3 Hz, 1H), 7.37 (d, J = 8.3 Hz, 1H), 7.20 (s, 1H), 3.58 (d, J = 12.8 Hz, 1H), 3.25 (d, J = 12.8 Hz, 1H), 3.17 (s, 3H), 2.31 – 2.21 (m, 1H), 2.20 – 2.06 (m, 2H), 2.01 – 1.94 (m, 1H), 1.41 (s, 3H). ^{13}C NMR (126 MHz, $CDCl_3$): δ 164.2, 142.5, 132.1, 129.1, 128.4, 127.7, 124.2, 119.5, 58.7, 36.9, 35.3, 34.9, 22.6, 12.9.

Methyl 4-(2-cyanoethyl)-2,4-dimethyl-1-oxo-1,2,3,4-tetrahydroiso-quinoline-6-carboxylate (6e)[10]

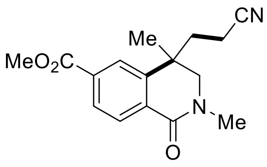

6e

According to **GP2**, workup and flash column chromatography (petroleum ether/ethyl acetate: 1/1) gave product **6e** (32.6 mg, 57% yield, 72 h) as a brown oil. ^{1}H NMR (500 MHz, $CDCl_3$): δ 8.22 (d, *J* = 7.8 Hz, 1H), 8.05 (d, *J* = 7.7 Hz, 1H), 7.89 (s, 1H), 3.96 (s, 3H), 3.61 (d, *J* = 12.9 Hz, 1H), 3.29 (d, *J* = 12.9 Hz, 1H), 3.20 (s, 3H), 2.38 – 2.21 (m, 1H), 2.18 – 2.08 (m, 2H), 2.05 – 1.96 (m, 1H), 1.46 (s, 3H). ^{13}C NMR (126 MHz, $CDCl_3$): δ 166.2, 163.3, 142.9, 133.2, 132.0, 129.4, 128.7, 125.5, 119.2, 58.4, 52.6, 37.0, 35.4, 34.9, 22.5, 12.9.

3-(2,4,7-Trimethyl-1-oxo-1,2,3,4-tetrahydroiso-quinolin-4-yl) propanenitrile (6f) and 3-(2,4,5-trimethyl-1-oxo-1,2,3,4-tetrahydro-isoquinolin-4-yl)propanenitrile (6f')[10]

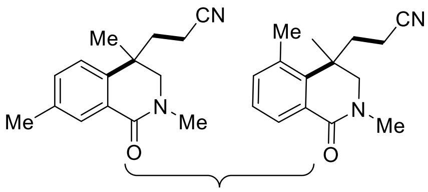

6f+6f'

According to **GP2**, workup and flash column chromatography (petroleum ether/ethyl acetate: 1/1) gave products **6f** and **6f**' (30.5 mg, 63% yield, 72 h) as a colorless oil. ^{1}H NMR (500 MHz, $CDCl_3$): δ 7.31 (d, *J* = 7.8 Hz, 1H), 7.11 (d, *J* = 7.8 Hz, 1H), 3.55 (d, *J* = 12.7 Hz, 1H), 3.20 (d, *J* = 12.7 Hz, 1H), 3.17 (s, 3H), 2.39 (s, 3H), 2.27 – 2.14 (m, 1H), 2.15 – 2.06 (m, 2H), 1.99 – 1.88 (m, 1H), 1.39 (s, 3H). ^{13}C NMR (126 MHz, $CDCl_3$): δ 164.4, 139.4, 137.6, 132.8, 129.5, 128.1, 124.2, 119.6, 58.9, 36.6, 35.4, 35.0, 22.7, 21.0, 13.0. 7.96 (s, 1H).

General procedure for the CN-K-catalyzed cyanomethylarylation of *N*-arylacrylamides (GP3)

To a 30 mL oven-dried sealed tube equipped with a magnetic stirring bar was added **7** (0.2 mmol, 1.0 equiv), **2d** (0.4 mmol, 2.0 equiv), and CN-K (4 mg). It was capped with a rubber septum, evacuated, and backfilled with argon. Then, degassed CH_3CN (8 mL) was added via syringe. The sealed tube was irradiated by two blue LEDs (460 ± 5 nm) and the reaction mixture was stirred at 80 °C for the indicated time (see Figure S1A). After complete consumption of the starting material (followed by TLC), the reaction mixture was concentrated under reduced pressure to evaporate the solvent, and the crude residue was purified by silica gel column chromatography to obtain the product **8**.

3-(1,3-Dimethyl-2-oxoindolin-3-yl)propanenitrile (8a)[12]

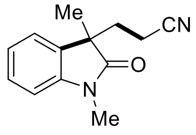

8a

According to **GP3**, workup and flash column chromatography (petroleum ether/ethyl acetate: 5/1) gave product **8a** (30.0 mg, 70% yield, 72 h) as a white solid. ^{1}H NMR (500 MHz, $CDCl_3$): δ 7.33 (t, *J* = 7.6 Hz, 1H), 7.20 (d, *J* = 7.1 Hz, 1H), 7.12 (t, *J* = 7.4 Hz, 1H), 6.89 (d, *J* = 7.7 Hz, 1H), 3.23 (s, 3H), 2.38 – 2.28 (m, 1H), 2.21 – 1.91 (m, 3H), 1.40 (s, 3H). ^{13}C NMR (126 MHz, $CDCl_3$): δ 178.9, 143.2, 131.7, 128.7, 123.1, 122.7, 118.8, 108.6, 47.4, 33.5, 26.4, 23.5, 12.9.

3-(5-Methoxy-1,3-dimethyl-2-oxoindolin-3-yl)propanenitrile (8b)[12]

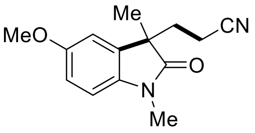

8b

According to **GP3**, workup and flash column chromatography (petroleum ether/ethyl acetate: 5/1) gave product **8b** (35.6 mg, 73% yield, 96 h) as a yellow oil. ^{1}H NMR (500 MHz, $CDCl_3$): δ 7.05 – 6.66 (m, 3H), 3.82 (s, 3H), 3.20 (s, 3H), 2.32 (m, 1H), 2.18 – 1.94 (m, 3H), 1.39 (s, 3H). ^{13}C NMR (126 MHz, $CDCl_3$): δ 178.5, 156.5, 136.6, 133.1, 118.9, 112.6, 110. 3, 108.90, 55.9, 47.8, 33.5, 26.4, 23.6, 12.9.

3-(1,3,5-Trimethyl-2-oxoindolin-3-yl)propanenitrile (8c)[12]

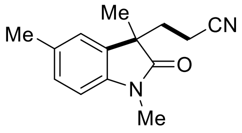

8c

According to **GP3**, workup and flash column chromatography (petroleum ether/ethyl acetate: 5/1) gave product **8c** (34.7 mg, 76% yield, 72 h) as a white solid. ^{1}H NMR (500 MHz, $CDCl_3$): δ 7.11 (d, *J* = 7.8 Hz, 1H), 7.00 (s, 1H), 6.77 (d, *J* = 7.9 Hz, 1H), 3.20 (s, 3H), 2.36 (s, 3H), 2.35 – 2.28 (m, 1H), 2.13 – 1.94 (m, 3H), 1.39 (s, 3H). ^{13}C NMR (126 MHz, $CDCl_3$): δ 178.9, 140.8, 132.7, 131.8, 128.9, 123.5, 118.9, 108.27, 47.4, 33.5, 26.3, 23.5, 21.2, 12.9.

3-(5-Fluoro-1,3-dimethyl-2-oxoindolin-3-yl)propanenitrile (8d)[13]

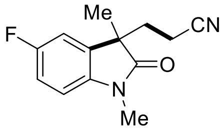

8d

According to **GP3**, workup and flash column chromatography (petroleum ether/ethyl acetate: 5/1) gave product **8d** (38.1 mg, 82% yield, 72 h) as a white solid. ^{1}H NMR (500 MHz, $CDCl_3$): δ 7.03 (t, *J* = 8.7 Hz, 1H), 6.96 (d, *J* = 7.4 Hz, 1H), 6.86 – 6.77 (m, 1H), 3.22 (s, 3H), 2.38 – 2.28 (m, 1H), 2.20 – 2.00 (m, 3H), 1.41 (s, 3H). ^{13}C NMR (126 MHz, $CDCl_3$): δ 178.5, 159.6 (d, *J* = 242.1 Hz), 139.1, 133.4 (d, *J* = 7.5 Hz), 118.57, 115.0 (d, *J* = 23.6 Hz), 110.9 (d, *J* = 24.7 Hz), 109.1 (d, *J* = 8.2 Hz), 47.8, 33.3, 26.5, 23.5, 12.9.

3-(5-Chloro-1,3-dimethyl-2-oxoindolin-3-yl)propanenitrile (8e)[13]

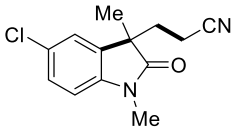

8e

According to **GP3**, workup and flash column chromatography (petroleum ether/ethyl acetate: 5/1) gave product **8e** (36.8 mg, 74% yield, 72 h) as a pale yellow solid. ^{1}H NMR (500 MHz, $CDCl_3$): δ 7.31 (d, *J* = 8.2 Hz, 1H), 7.18 (s, 1H), 6.81 (d, *J* = 8.3 Hz, 1H), 3.22 (s, 3H), 2.37 – 2.28 (m, 1H), 2.19 – 2.00 (m, 3H), 1.41 (s, 3H). ^{13}C NMR (126 MHz, $CDCl_3$): δ 178.4, 141.8, 133.5, 128.7, 128.5, 123.3, 118.5, 109.53, 47.6, 33.3, 26.5, 23.5, 12.9.

3-(5-Bromo-1,3-dimethyl-2-oxoindolin-3-yl)propanenitrile (8f)[13]

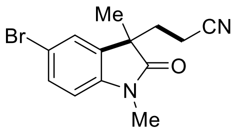

8f

According to **GP3**, workup and flash column chromatography (petroleum ether/ethyl acetate: 5/1) gave product **8f** (44.4 mg, 76% yield, 72 h) as a pale yellow solid. ^{1}H NMR (500 MHz, $CDCl_3$): δ 7.45 (d, J = 8.2 Hz, 1H), 7.31 (s, 1H), 6.77 (d, J = 8.2 Hz, 1H), 3.21 (s, 3H), 2.41 – 2.27 (m, 1H), 2.16 – 1.99 (m, 3H), 1.40 (s, 3H). ^{13}C NMR (126 MHz, $CDCl_3$): δ 178.3, 142.3, 133.9, 131.6, 126.0, 118.5, 115.8, 110.0, 47.5, 33.3, 26.5, 23.5, 12.9.

3-(1,3-Dimethyl-2-oxo-5-(trifluoromethyl)indolin-3-yl)propanenitrile (8g)[15, 16]

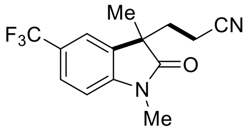

8g

According to **GP3**, workup and flash column chromatography (petroleum ether/ethyl acetate: 5/1) gave product **8g** (41.2 mg, 73% yield, 96 h) as a white solid. ^{1}H NMR (500 MHz, $CDCl_3$): δ 7.63 (d, J = 8.0 Hz, 1H), 7.44 (s, 1H), 6.98 (d, J = 8.1 Hz, 1H), 3.27 (s, 3H), 2.46 – 2.28 (m, 1H), 2.22 – 1.95 (m, 3H), 1.44 (s, 3H). ^{13}C NMR (126 MHz, $CDCl_3$): δ 178.8, 146.2, 132.4, 126.6 (q, J = 3.8 Hz), 125.3 (q, J = 32.7 Hz), 124.2 (q, J = 271.6 Hz), 119.8 (q, J = 3.5 Hz) 108.4, 47.3, 33.2, 26.6, 23.4, 12.8.

3-(2-Cyanoethyl)-1,3-dimethyl-2-oxoindoline-5-carbonitrile (8h)[13]

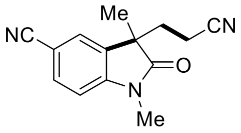

8h

According to **GP3**, workup and flash column chromatography (petroleum ether/ethyl acetate: 5/1) gave product **8h** (37.8 mg, 79% yield, 60 h) as a white solid. ^{1}H NMR (500 MHz, $CDCl_3$): δ 7.68 (d, J = 8.1 Hz, 1H), 7.48 (s, 1H), 6.98 (d, J = 8.1 Hz, 1H), 3.27 (s, 3H), 2.42 – 2.28 (m, 1H), 2.22 – 2.01 (m, 3H), 1.44 (s, 3H). ^{13}C NMR (126 MHz, $CDCl_3$): δ 178.6, 147.1, 134.1, 132.9, 126.2, 118.9, 118.2, 109.1, 106.3, 47.1, 33.0, 26.6, 23.4, 12.9.

Methyl 3-(2-cyanoethyl)-1,3-dimethyl-2-oxoindoline-5-carboxylate (8i)[13]

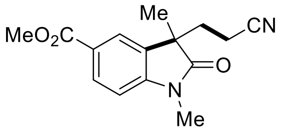

8i

According to **GP3**, workup and flash column chromatography (petroleum ether/ethyl acetate: 3/1) gave product **8i** (40.3 mg, 74% yield, 72 h) as a white solid. ^{1}H NMR (500 MHz, $CDCl_3$): δ 8.08 (d, *J* = 8.0 Hz, 1H), 7.88 (s, 1H), 6.94 (d, *J* = 8.1 Hz, 1H), 3.93 (s, 3H), 3.27 (s, 3H), 2.42 – 2.30 (m, 1H), 2.22 – 1.98 (m, 3H), 1.44 (s, 3H). ^{13}C NMR (126 MHz, $CDCl_3$): δ 179.2, 166.6, 147.3, 131.8, 131.4, 125.0, 124.0, 118.5, 108.1, 52.2, 47.2, 33.2, 26.6, 23.5, 12.9.

3-(5-Acetyl-1,3-dimethyl-2-oxoindolin-3-yl)propanenitrile (8j)[13]

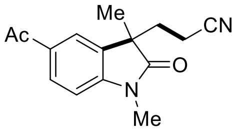

8j

According to **GP3**, workup and flash column chromatography (petroleum ether/ethyl acetate: 3/1) gave product **8j** (37.4 mg, 73% yield, 72 h) as a white solid. ^{1}H NMR (500 MHz, $CDCl_3$): δ 7.98 (d, *J* = 8.1 Hz, 1H), 7.85 (s, 1H), 6.94 (d, *J* = 8.1 Hz, 1H), 3.28 (s, 3H), 2.61 (s, 3H), 2.43 – 2.29 (m, 1H), 2.18 – 2.03 (m, 3H), 1.44 (s, 3H). ^{13}C NMR (126 MHz, $CDCl_3$): δ 196.7, 179.3, 147.5, 132.6, 132.2, 130. 8, 122.5, 118.4, 108.0, 47.2, 33.2, 26.6, 26.4, 23.5, 12.9.

3-(1,3,7-Trimethyl-2-oxoindolin-3-yl)propanenitrile (8k)[12]

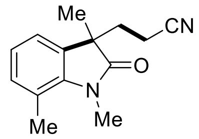

8k

According to **GP3**, workup and flash column chromatography (petroleum ether/ethyl acetate: 5/1) gave product **8k** (31.5 mg, 69% yield, 72 h) as a white solid. ^{1}H NMR (500 MHz, $CDCl_3$): δ 6.95 – 7.08 (m, 3H), 3.58 – 3.35 (m, 3H), 2.59 (s, 3H), 2.39 – 2.25 (m, 1H), 2.12 – 1.92 (m, 3H), 1.37 (s, 3H). ^{13}C NMR (126 MHz, $CDCl_3$): δ 179.7, 140.9, 132.4, 123.0, 120.5, 120.3, 118.9, 46.7, 33.7, 29.6, 23.9, 19.0, 12.9.

3-(1,3,6-Trimethyl-2-oxoindolin-3-yl)propanenitrile (8l) and 3-(1,3,4-trimethyl-2-oxoindolin-3-yl)propanenitrile (8l')[12]

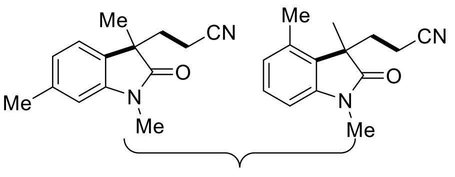

8l+8l'

According to **GP3**, workup and flash column chromatography (petroleum ether/ethyl acetate: 5/1) gave products **8l+8l'** (28.3 mg, 62% yield, 84 h) as a colorless oil. ^{1}H NMR (500 MHz, $CDCl_3$) δ 7.22 (t, *J* = 7.7 Hz, 1.6H), 7.06 (d, *J* = 7.4 Hz, 1H), 6.92 (d, *J* = 7.4 Hz, 1H), 6.88 (d, *J* = 7.7 Hz, 1.6H), 6.76 – 6.67 (m, 2.6H), 3.23 – 2.19 (m, 7.8H), 2.44 – 2.36 (m, 9.4H), 2.36 – 2.25 (m, 2.6H), 2.15 – 1.87 (m, 6.2H), 1.48 (s, 4.8H), 1.38 (s, 3H). ^{13}C NMR (126 MHz, $CDCl_3$) δ 179.2, 178.9, 143.5, 143.3, 138.9, 134.5, 128.7, 128.6, 128.2, 125.6, 123.6, 122.4, 118.9, 118.7, 109.5, 106.3, 48.4, 47.2, 33.5, 31.5, 26.4, 26.3, 23.6, 21.9, 21.8, 18.2, 13.1, 12.9.

3-(1,3-Dimethyl-2-oxo-2,3-dihydro-1H-benzo[*g*] indol-3-yl)propane-nitrile (8m)[13]

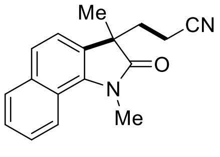

8m

According to **GP3**, workup and flash column chromatography (petroleum ether/ethyl acetate: 5/1) gave products **8m** (40.7 mg, 77% yield, 72 h) as a pale yellow oil. ^{1}H NMR (500 MHz, $CDCl_3$): δ 7.78 (d, *J* = 8.4 Hz, 1H), 7.61 – 7.53 (m, 2H), 7.51 – 7.39 (m, 2H), 6.99 (d, *J* = 7.5 Hz, 1H), 3.55 (s, 3H), 2.93 – 2.73 (m, 1H), 2.36 – 1.92 (m, 3H), 1.69 (s, 3H). ^{13}C NMR (126 MHz, $CDCl_3$): δ 171.8, 136.2, 135.7, 133.5, 127.3, 127.0, 126.8, 123.0, 122.6, 119.5, 119.1, 109.0, 47.0, 38.3, 31.5, 29.9, 13.8.

3-(1-Methyl-2-oxo-1,2,5,6-tetrahydro-4*H*-pyrrolo [3,2,1-*ij*]quinolin-1-yl) propan-enitrile (8n)[12]

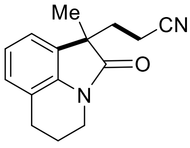

8n

According to **GP3**, workup and flash column chromatography (petroleum ether/ethyl acetate: 4/1) gave products **8n** (33.6 mg, 70% yield, 72 h) as a yellow solid. ^{1}H NMR (500 MHz, $CDCl_3$): δ 7.13 – 6.88 (m, 3H), 3.80 – 3.63 (m, 2H), 2.80 (s, 2H), 2.47 – 2.25 (m, 1H), 2.18 – 1.96 (m, 5H), 1.41 (s, 3H). ^{13}C NMR (126 MHz, $CDCl_3$): δ 177.8, 139.0, 130.3, 127.5, 122.5, 120.7, 120.5, 118.9, 48.7, 38.9, 33.3, 24. 6, 23.2, 21.2, 13.0.

3-(1-Isopropyl-3-methyl-2-oxoindolin-3-yl)propanenitrile (8o)[14]

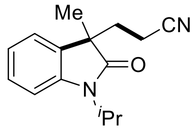

8o

According to **GP3**, workup and flash column chromatography (petroleum ether/ethyl acetate: 4/1) gave products **8o** (36.8 mg, 76% yield, 72 h) as a colorless oil. ^{1}H NMR (500 MHz, $CDCl_3$): δ 7.31 – 7.27 (m, 1H), 7.19 (d, *J* = 7.1 Hz, 1H), 7.09 (t, *J* = 7.4 Hz, 1H), 7.05 (d, *J* = 7.8 Hz, 1H), 4.71 – 4.55 (m, 1H), 2.39 – 2.25 (m, 1H), 2.13 – 1.87 (m, 3H), 1.48 (d, *J* = 6.8 Hz, 6H), 1.38 (s, 3H). ^{13}C NMR (126 MHz, $CDCl_3$): δ 178.5, 141.8, 132.2, 128.4, 122.9, 122.5, 118.9, 110.2, 47.1, 43.9, 33.7, 23.7, 19.5, 19.4, 12.7.

3-(1-Benzyl-3-methyl-2-oxoindolin-3-yl)propanenitrile (8p)[12]

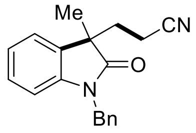

8p

According to **GP3**, workup and flash column chromatography (petroleum ether/ethyl acetate: 4/1) gave products **8p** (41.8 mg, 72% yield, 84 h) as a colorless oil. ^{1}H NMR (500 MHz, $CDCl_3$): δ 7.37 – 7.31 (m, 2H), 7.30 – 7.26 (m, 3H), 7.24 – 7.18 (m, 2H), 7.08 (t, *J* = 7.4 Hz, 1H), 6.79 (d, *J* = 7.8 Hz, 1H), 4.91 (q, *J* = 15.6 Hz, 2H), 2.47 – 2.30 (m, 1H), 2.18 – 1.94 (m, 3H), 1.45 (s, 3H). ^{13}C NMR (126 MHz, $CDCl_3$): δ 179.1, 142.3, 135.8, 131.7, 128.9, 128.7, 127.9, 127.3, 123.1, 122.7, 118.9, 109.6, 47.4, 43.9, 33.6, 23.8, 12.9.

3-(3-Methyl-2-oxo-1-phenylindolin-3-yl)propanenitrile (8q)[15]

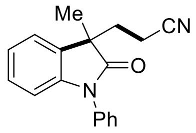

8q

According to **GP3**, workup and flash column chromatography (petroleum ether/ethyl acetate: 4/1) gave products **8q** (33.0 mg, 68% yield, 84 h) as a pale yellow oil. ^{1}H NMR (500 MHz, $CDCl_3$): δ 7.54 (t, *J* = 7.2 Hz, 2H), 7.46 – 7.37 (m, 3H), 7.26 (s, 2H), 7.16 (t, *J* = 7.2 Hz, 1H), 6.87 (d, *J* = 7.7 Hz, 1H), 2.48 – 2.39 (m, 1H), 2.30 – 2.10 (m, 3H), 1.53 (s, 3H). ^{13}C NMR (126 MHz, $CDCl_3$): δ 178.4, 143.2, 134.2, 131.5, 129.7, 128.7, 128.3, 126.46, 123.6, 123.0, 118.8, 109.9, 47.5, 33.8, 24.0, 13.0.

3-(3-Benzyl-2-oxo-1-phenylindolin-3-yl)propanenitrile (8r)[17]

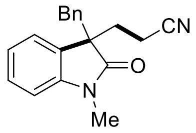

8r

According to **GP3**, workup and flash column chromatography (petroleum ether/ethyl acetate: 4/1) gave products **8r** (43.0 mg, 74% yield, 72 h) as a white solid. ^{1}H NMR (500 MHz, $CDCl_3$): δ 7.23 (d, *J* = 7.8 Hz, 1H), 7.14 (d, *J* = 7.2 Hz, 1H), 7.12 – 7.02 (m, 4H), 6.80 (d, *J* = 7.2 Hz, 2H), 6.63 (d, *J* = 7.8 Hz, 1H), 3.12 (d, *J* = 13.1 Hz, 1H), 3.02 (d, *J* = 13.0 Hz, 1H), 2.97 (s, 3H), 2.56 – 2.46 (m, 1H), 2.27 – 2.18 (m, 1H), 2.12 – 2.04 (m, 1H), 1.97 – 1.88 (m, 1H). ^{13}C NMR (126 MHz, $CDCl_3$): δ 177.5, 143.8, 134.8, 129.9, 128.8, 128.7, 127.7, 126.8, 123.7, 122.7, 118.8, 108.3, 53.7, 44.0, 32.0, 26.0, 13.0.

3-(1-Methyl-2-oxo-3-phenylindolin-3-yl)propanenitrile (8s)[16]

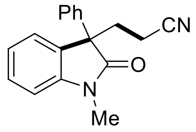

8s

According to **GP3**, workup and flash column chromatography (petroleum ether/ethyl acetate: 5/1) gave products **8s** (13.3 mg, 24% yield, 72 h) as a white solid. ^{1}H NMR (500 MHz, $CDCl_3$): δ 7.41 – 7.30 (m, 5H), 7.28 – 7.24 (m, 2H), 7.17 (t, *J* = 7.3 Hz, 1H), 6.94 (d, *J* = 7.2 Hz, 1H), 3.24 (s, 3H), 2.78 – 2.88 (m, 1H), 2.54 – 2.44 (m, 1H), 2.23 – 2.08 (m, 2H). ^{13}C NMR (126 MHz, $CDCl_3$): δ 177.1, 143.8, 138.4, 130.0, 129.2, 128.9, 127.9, 126.7, 124.8, 123.2, 118.8, 108.9, 55.4, 33.3, 26.6, 13.2.

2-(1-Methyl-4-phenyl-1,2,3,4-tetrahydroquinolin-3-yl)acetonitrile (8t)[16]

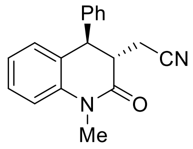

trans-**8t**

According to **GP3**, workup and flash column chromatography (petroleum ether/ethyl acetate: 5/1) gave products **8t** (12.2 mg, 22% yield, 72 h) as a white solid. *trans*-**8t**: ^{1}H NMR (500 MHz, $CDCl_3$): δ 7.44 (t, *J* = 7.6 Hz, 2H), 7.39 (t, *J* = 6.2 Hz, 1H), 7.31 (t, *J* = 7.9 Hz, 1H), 7.27 (d, *J* = 7.0 Hz, 2H), 7.08 (d, *J* = 8.3 Hz, 1H), 6.97 (t, *J* = 7.7 Hz, 1H), 6.68 (d, *J* = 7.7 Hz, 1H), 4.26 (d, *J* = 13.2 Hz, 1H), 3.47 (s, 3H), 3.10 – 3.04 (m, 1H), 2.94 (dd, *J* = 16.5, 4.9 Hz, 1H), 2.25 (dd, *J* = 16.9, 4.3 Hz, 1H). ^{13}C NMR (126 MHz, $CDCl_3$): δ 168.1, 139.5, 138.5, 129.5, 129.0, 128.4, 128.3, 128.3, 128.2, 123.5, 117.7, 114.9, 45.8, 43.8, 30.4, 17.4.

General procedure for the CN-K-catalyzed cyano-methylarylation of *N*-benzoyl acrylamides (GP4)

To a 30 mL oven-dried sealed tube equipped with a magnetic stirring bar was added **9** (0.2 mmol, 1.0 equiv), **2d** (0.4 mmol, 2.0 equiv), and CN-K (8 mg). It was capped with a rubber septum, evacuated, and backfilled with argon. Then, degassed CH_3CN (8 mL) was added via syringe. The sealed tube was irradiated by two blue LEDs (460 ± 5 nm) and the reaction mixture was stirred at 80 °C for the indicated time (see Figure S1A). After complete consumption of the starting material (followed by TLC), the reaction mixture was concentrated under reduced pressure to evaporate the solvent, and the crude residue was purified by silica gel column chromatography to obtain the product **10**.

3-(2,4-Dimethyl-1,3-dioxo-1,2,3,4-tetrahydroisoquinolin-4-yl)propanenitrile (10a)[12, 15]

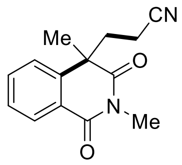

10a

According to **GP4**, workup and flash column chromatography (petroleum ether/ethyl acetate: 5/1) gave products **10a** (29.1 mg, 60% yield, 72 h) as a pale yellow oil. ^{1}H NMR (500 MHz, $CDCl_3$): δ 8.30 (d, *J* = 7.8 Hz, 1H), 7.71 (t, *J* = 7.6 Hz, 1H), 7.51 (t, *J* = 7.5 Hz, 1H), 7.43 (d, *J* = 7.8 Hz, 1H), 3.40 (s, 3H), 2.82 – 2.73 (m, 1H), 2.35 – 2.22 (m, 1H), 2.10 – 1.86 (m, 2H), 1.65 (s, 3H). ^{13}C NMR (126 MHz, $CDCl_3$): δ 175.2, 163.8, 141.0, 134.6, 129.6, 128.3, 125.0, 124.9, 118.2, 47.0, 36.7, 30.2, 27.4, 13.6.

3-(2,4,6-Trimethyl-1,3-dioxo-1,2,3,4-tetrahydroisoquinolin-4-yl)propanenitrile (10b)[15]

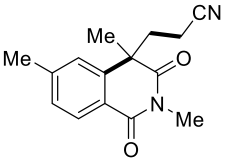

10b

According to **GP4**, workup and flash column chromatography (petroleum ether/ethyl acetate: 5/1) gave products **10b** (33.8 mg, 66% yield, 72 h) as a white solid. ^{1}H NMR (500 MHz, $CDCl_3$): δ 8.16 (d, *J* = 7.9 Hz, 1H), 7.31 (d, *J* = 8.0 Hz, 1H), 7.19 (s, 1H), 3.38 (s, 3H), 2.85 – 2.65 (m, 1H), 2.48 (s, 3H), 2.32 – 2.19 (m, 1H), 2.08 – 1.98 (m, 1H), 1.97 – 1.88 (m, 1H), 1.63 (s, 3H). ^{13}C NMR (126 MHz, $CDCl_3$): δ 175.3, 163.8, 145.7, 141.1, 129.5, 129.4, 125.3, 122.5, 118.3, 47.0, 36.8, 30.2, 27.3, 22.0, 13.6.

3-(6-Fluoro-2,4-dimethyl-1,3-dioxo-1,2,3,4-tetrahydroisoquinolin-4-yl)propanenitrile (10c)[15]

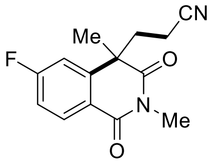

10c

According to **GP4**, workup and flash column chromatography (petroleum ether/ethyl acetate: 5/1) gave products **10c** (36.4 mg, 70% yield, 72 h) as a white solid. ^{1}H NMR (500 MHz, $CDCl_3$): δ 8.33 (t, *J* = 7.1 Hz, 1H), 7.22 (t, *J* = 8.3 Hz, 1H), 7.10 (d, *J* = 9.2 Hz, 1H), 3.39 (s, 3H), 2.83 – 2.72 (m, 1H), 2.25 – 2.17 (m, 1H), 2.13 – 1.92 (m, 2H), 1.65 (s, 3H). ^{13}C NMR (126 MHz, $CDCl_3$): δ 174.6, 166.6 (d, *J* = 257.2 Hz), 162.8, 144.1 (d, *J* = 8.6 Hz), 132.7 (d, *J* = 9.7 Hz), 121.5, 117.9, 116.3 (d, *J* = 22.0 Hz), 112.0 (d, *J* = 23.1 Hz), 47.2, 36.8, 30.0, 27.4, 13.6.

Scale-up reaction

To a 250 mL oven-dried Schlenk tube equipped with a magnetic stirring bar was added **1a** (378.5 mg, 2 mmol), **2d** (1.24 g, 4 mmol), CN-K (120 mg) under argon atmosphere. Then, degassed CH_3CN (120 mL) was added via syringe. The Schlenk tube was irradiated by blue LEDs (see Figure S2) and the reaction mixture was stirred without extra heating (55–60 °C) for 120 h. Then, the solvent was removed by evaporation and the residue was purified via chromatography on silica gel using petroleum ether/ethyl acetate 2:1 to 1:1 to afford the desired product **3a** (0.28 g, 62% yield).

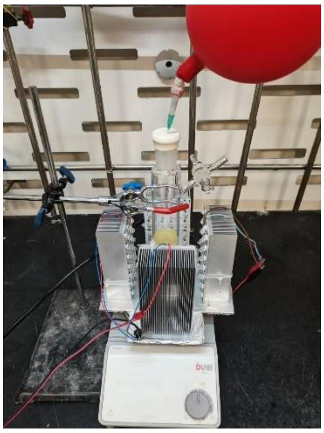

Figure S2 Experimental setup in batch.

Recycling experiments

To a 10 mL oven-dried sealed tube equipped with a magnetic stirring bar was added **1a** (0.1 mmol, 1.0 equiv), **2d** (0.2 mmol, 2.0 equiv), and CN-K (6 mg). It was capped with a rubber septum, evacuated, and backfilled with argon. Then, degassed CH_3CN (6 mL) was added via syringe. The sealed tube was irradiated by two blue LEDs (460 ± 5 nm) and the reaction mixture was stirred without extra heating (at 40–45 °C). After completion, the reaction mixture was centrifuged to separate the CN-K solid catalyst and the liquid mixture. Then, the catalyst CN-K was washed with CH_3CN (3 × 5 mL) and reused in the subsequent reaction. The combined liquid was concentrated and the residue was analyzed by ^{1}H NMR using 1,3,5-trimethoxybenzene as an internal standard.

Synthesis of 2-((1,3-dimethyl-2-oxoindolin-3-yl)methyl)butanenitrile (11)[16]

To a 30 mL oven-dried sealed tube equipped with a magnetic stirring bar was added **7a** (0.2 mmol, 1.0 equiv), **2d** (0.4 mmol, 2.0 equiv), and CN-K (8 mg). It was capped with a rubber septum, evacuated, and backfilled with argon. Then, degassed *n*-BuCN (8 mL) was added via syringe. The sealed tube was irradiated by two blue LEDs (460 ± 5 nm) and the reaction mixture was stirred at 80 °C for the indicated time (see Figure S1A). After complete consumption of the starting material (followed by TLC), the reaction mixture was concentrated under reduced pressure to evaporate the solvent. The residue was purified via chromatography on silica gel using petroleum ether/ethyl acetate 7:1 to 5:1 to afford the desired product **11** (21.8 mg, 45% yield, 1:1 dr). ^{1}H NMR (500 MHz, $CDCl_3$): δ 7.38 – 7.28 (m, 3H), 7.16 (t, *J* = 7.5 Hz, 2H), 7.09 (t, *J* = 7.5 Hz, 1H), 6.90 (d, *J* = 7.8 Hz, 2H), 3.25 (s, 3H), 3.24 (s, 3H), 2.41 – 2.07 (m, 5H), 1.88 – 1.82 (m, 1H), 1.56 – 1.47 (m, 4H), 1.43 (s, 3H), 1.41 (s, 3H), 0.98 (t, *J* = 7.5 Hz, 6H). ^{13}C NMR (126 MHz, $CDCl_3$): δ 179.4, 179.3, 143.5, 143.2, 132.2, 131.6, 128.8, 128.6, 123.6, 123.1, 122.7, 122.5, 120.9, 108.7, 108.5, 47.7, 47.0, 39.6, 39.6, 29.3, 28.9, 26.7, 26.6, 26.4, 25.0, 24.5, 11.3, 11.1.

Ritter synthesis of *N*-*tert*-butylpropanamide (12)[18]

To a solution of **8a** (42.9 mg, 0.2 mmol, 1.0 equiv) in *tert*-butyl acetate (1 mL, 0.2 M), excess H_2SO_4 (ca. 3 drops delivered with a Pasteur pipet) was added. The reaction mixture was stirred at 70 °C for 3 h. After cooling, the mixture was quenched with saturated aqueous $NaHCO_3$, extracted (3 × 10 mL) with EtOAc, and washed with brine (20 mL). The combined organic layers were sequentially dried over $MgSO_4$, filtered, and concentrated under vacuum. The residue was purified via chromatography on silica gel using petroleum ether/ethyl acetate 2:1 to 1:1 to afford the desired product **12** (55.4 mg, 96% yield). ^{1}H NMR (500 MHz, $CDCl_3$): δ 7.27 (s, 1H), 7.19 (d, *J* = 6.9 Hz, 1H), 7.06 (t, *J* = 7.4 Hz, 1H), 6.84 (d, *J* = 7.7 Hz, 1H), 4.10 (t, *J* = 9.4 Hz, 1H), 3.69 (t, *J* = 9.1 Hz, 1H), 3.21 (s, 1H), 2.33 – 2.23 (m, 1H), 2.18 – 2.08 (m, 1H), 2.03 – 1.95 (m, 1H), 1.90 – 1.82 (m, 1H), 1.39 (s, 3H). ^{13}C NMR (126 MHz, $CDCl_3$): δ 180.3, 171.2, 143.0, 133.4, 127.9, 122.9, 122.8, 108.0, 51.1, 47.8, 33.76, 32.5, 28.7, 26.2, 23.6. HRMS (ESI) m/z: calcd for $[M + Na]^+$, $C_{17}H_{24}N_2NaO_2$ 311.1730; found, 311.1730.

Witte–Seeliger synthesis of oxazoline (13)[19]

To a solution of **8a** (42.9 mg, 0.2 mmol, 1.0 equiv) and $Cd(OAc)_2{\cdot}2H_2O$ (11 mg, 0.04 mmol, 20 mol %) in toluene (800 µL), ethanolamine (180 µL, 1.0 mmol, 5.0 equiv) was added. The reaction mixture was heated at 130 °C for 36 h. The mixture was extracted (3 × 10 mL) with EtOAc and the combined organic layers were sequentially dried over $MgSO_4$, filtered, and concentrated under vacuo. The residue was purified via chromatography on silica gel using ethyl acetate/methanol 50:1 to 20:1 to afford the desired product **13** (21.2 mg, 41% yield). ^{1}H NMR (500 MHz, $CDCl_3$): δ 7.27 (s, 1H), 7.19 (d, *J* = 6.9 Hz, 1H), 7.06 (t, *J* = 7.4 Hz, 1H), 6.84 (d, *J* = 7.7 Hz, 1H), 4.10 (t, *J* = 9.4 Hz, 2H), 3.69 (t, *J* = 9.1 Hz, 2H), 3.21 (s, 3H), 2.33 – 2.23 (m, 1H), 2.18 – 2.08 (m, 1H), 2.03 – 1.95 (m, 1H), 1.90 – 1.82 (m, 1H), 1.39 (s, 3H). ^{13}C NMR (126 MHz, $CDCl_3$): δ 179.9, 167.6, 143.4, 133.1, 128.0, 122.7, 122.6, 108.1, 67.1, 54.3, 47.8, 34.1, 26.2, 23.7, 23.4. HRMS (ESI) m/z: calcd for $[M + Na]^+$, $C_{15}H_{18}N_2NaO_2$ 281.1260; found, 281.1255.

Reductive cyclization for the synthesis of tricyclic indoline (14)[20]

To a suspension of $LiAlH_4$ (15.2 mg, 0.4 mmol, 2.0 equiv) in THF (800 µL) at room temperature, **8a** (42.9 mg, 0.2 mmol, 1.0 equiv) was added dropwise as a solution in THF (200 µL, final volume = 1 mL, 0.2 M). The resulting mixture was stirred at room temperature for 3 h. The reaction was carefully quenched with water and extracted (3 × 10 mL) with EtOAc. The combined organic layers were washed with brine (20 mL) and the combined organic layers were sequentially dried over $MgSO_4$, filtered, and concentrated under vacuo. The residue was purified via chromatography on silica gel using petroleum ether/ethyl acetate 1:1 to 1:2 to afford the desired product **14** (24.3 mg, 60% yield). ^{1}H NMR (500 MHz, $CDCl_3$): δ 7.11 (t, *J* = 7.5 Hz, 1H), 7.00 (d, *J* = 7.1 Hz, 1H), 6.72 (t, *J* = 7.2 Hz, 1H), 6.54 (d, *J* = 7.7 Hz, 1H), 3.69 (d, *J* = 14.9 Hz, 1H), 2.91 (t, *J* = 11.3 Hz, 1H), 2.74 (d, *J* = 12.2 Hz, 1H), 2.70 (s, 3H), 1.99 (s, 1H), 1.63 – 1.52 (m, 3H), 1.44 – 1.36 (m, 4H). ^{13}C NMR (126 MHz, $CDCl_3$): δ 150.0, 138.6, 127.4, 121.1, 118.2, 108.0, 86.4, 39.5, 34.6, 32.2, 21.7, 21.6. HRMS (ESI) m/z: calcd for $C_{13}H_{19}N_2$ $[M + H]^+$, 203.1542; found, 203.1539.

Synthesis of 1,3-dimethyl-3-(4-phenylbutyl)indolin-2-one (15)[21]

To a 30 mL oven-dried sealed tube equipped with a magnetic stirring bar was added **7a** (0.2 mmol, 1.0 equiv), **2d** (0.4 mmol, 2.0 equiv), and CN-K (4 mg). It was capped with a rubber septum, evacuated, and backfilled with argon. Then, degassed CH_3CN (8 mL) was added via syringe. The sealed tube was irradiated by two blue LEDs (460 ± 5 nm) and the reaction mixture was stirred at 80 °C for the indicated time (see Figure S1A). After complete consumption of the starting material (followed by TLC), the reaction mixture was concentrated under reduced pressure to evaporate the solvent. The residue was purified via chromatography on silica gel using petroleum ether/ethyl acetate 15:1 to 10:1 to afford the by-product **15** (13.5 mg, 23% yield). ^{1}H NMR (500 MHz, $CDCl_3$): δ 7.29 – 7.20 (m, 3H), 7.19 – 7.11 (m, 2H), 7.10 – 7.02 (m, 3H), 6.84 (d, *J* = 7.7 Hz, 1H), 3.20 (s, 3H), 2.46 (t, *J* = 8.0 Hz, 2H), 1.98 – 1.89 (m, 1H), 1.82 – 1.72 (m, 1H), 1.56 – 1.41 (m, 2H), 1.35 (s, 3H),

1.13 – 1.01 (m, 1H), 0.98 – 0.85 (m, 1H). ^{13}C NMR (126 MHz, $CDCl_3$): δ 180.8, 143.3, 142.6, 134.2, 128.3, 128.3, 127.7, 125.6, 122.5, 122.5, 107.9, 48.4, 38.3, 35.7, 31.7, 26.2, 24.3, 23.8. HRMS (ESI) m/z: calcd for $[M + Na]^+$, $C_{20}H_{23}NNaO$ 316.1671; found, 316.1675.

^{1}H NMR and ^{13}C NMR spectra of products

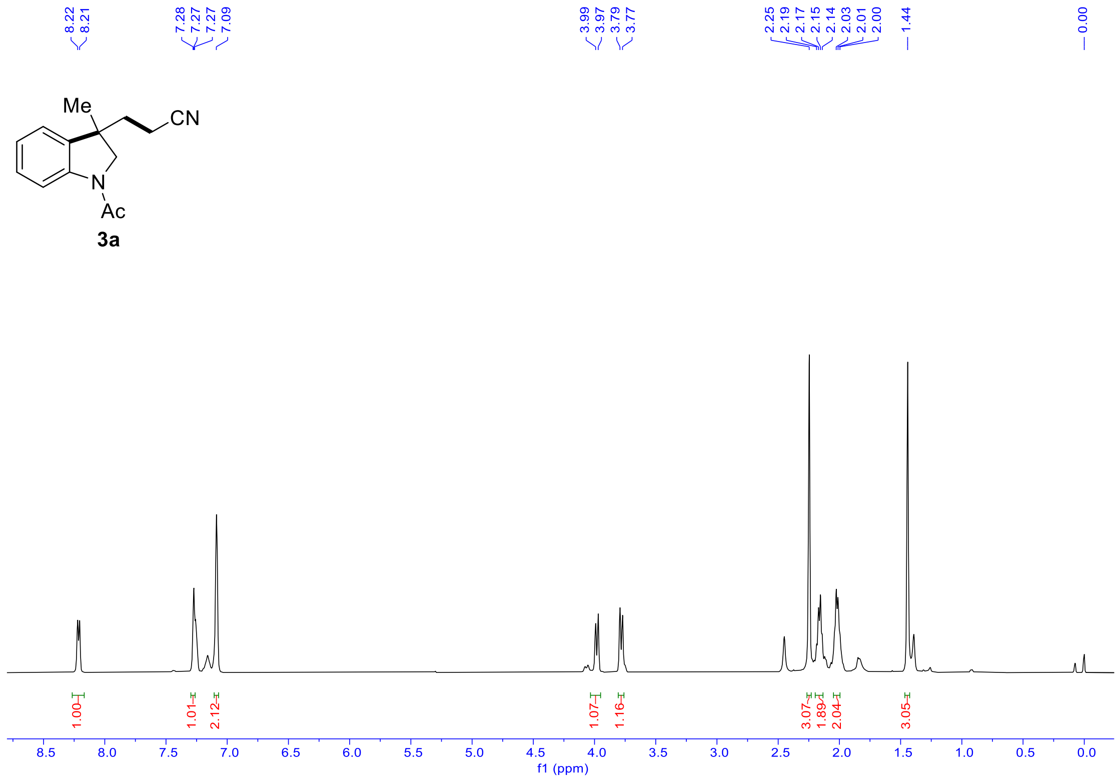

Figure S3: ^{1}H NMR of **3a**, ($CDCl_3$, 500 MHz)

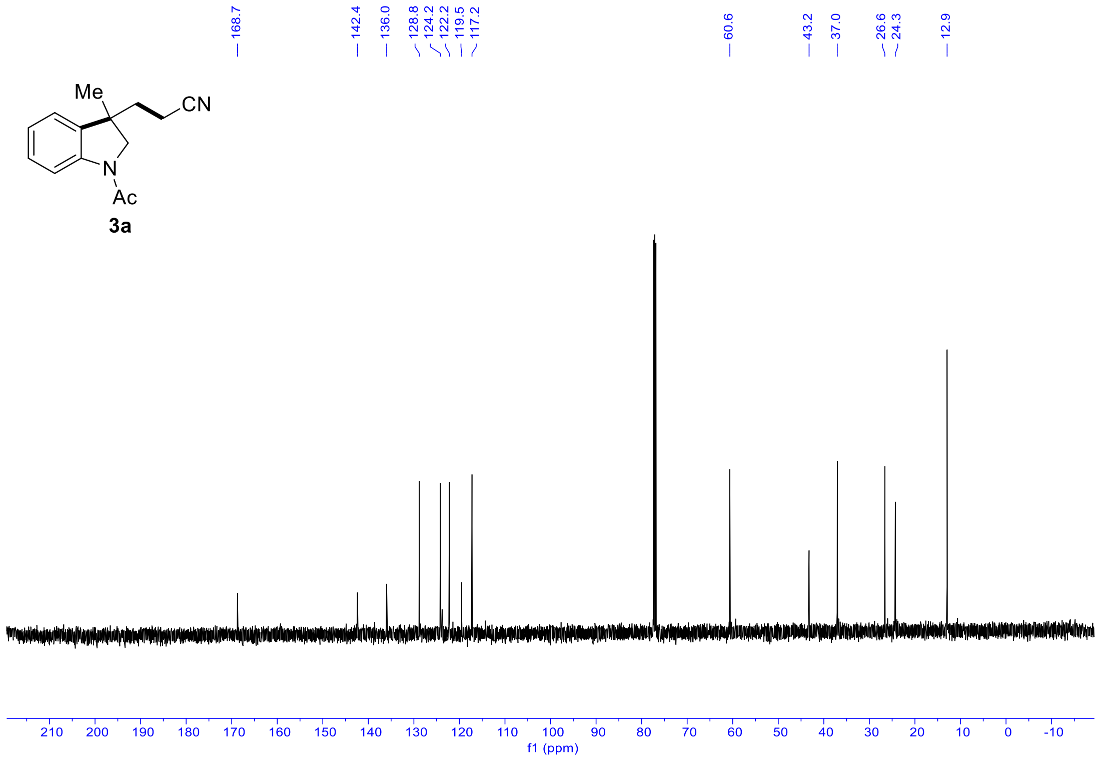

Figure S4: ^{13}C NMR of **3a**, ($CDCl_3$, 126 MHz)

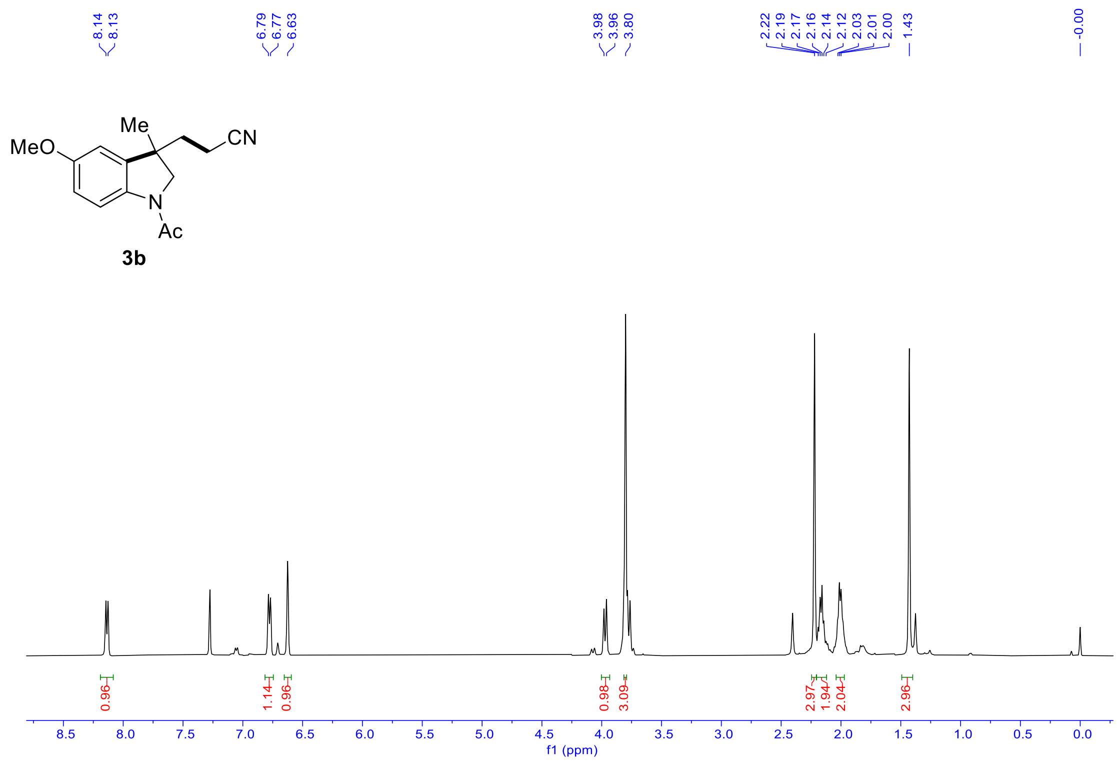

Figure S5: ^{1}H NMR of **3b**, ($CDCl_3$, 500 MHz)

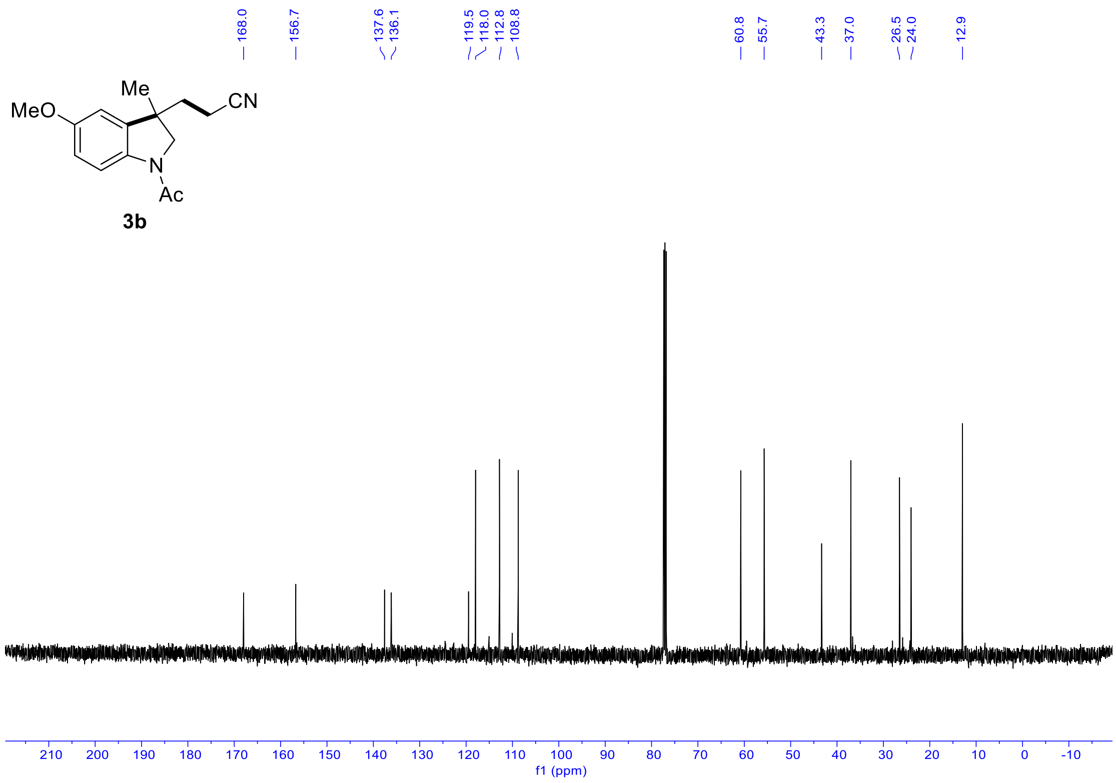

Figure S6: ^{13}C NMR of **3b**, ($CDCl_3$, 126 MHz)

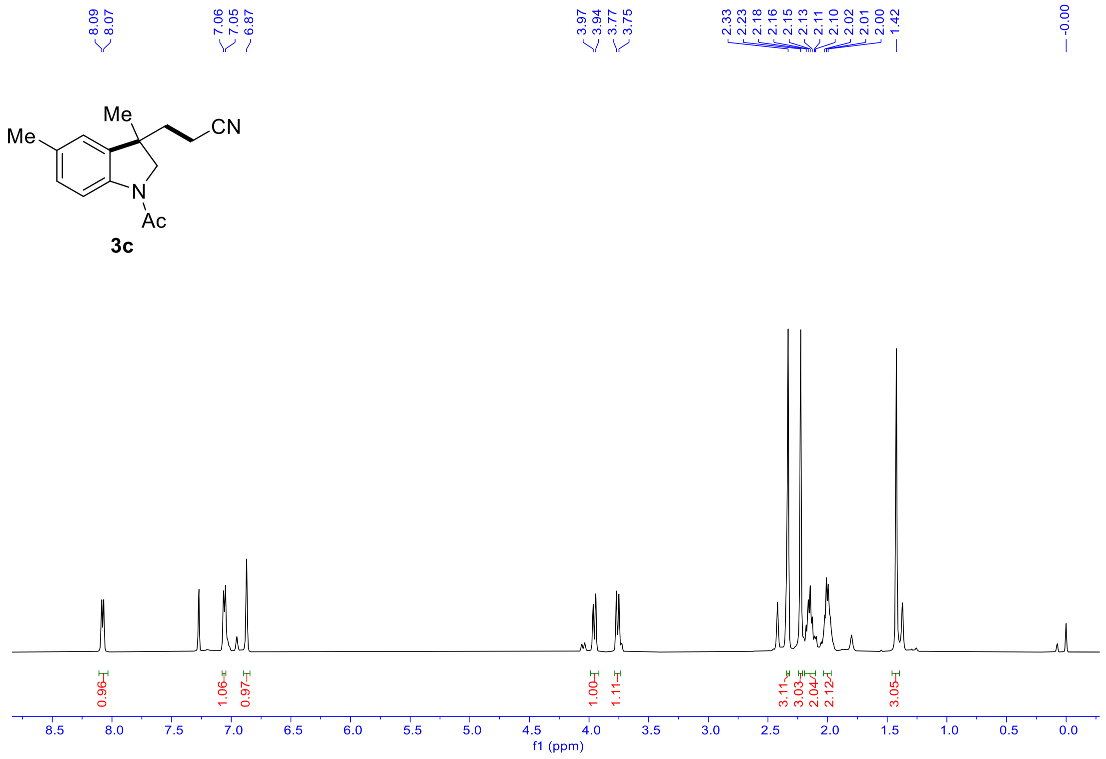

Figure S7: ^{1}H NMR of **3c**, ($CDCl_3$, 500 MHz)

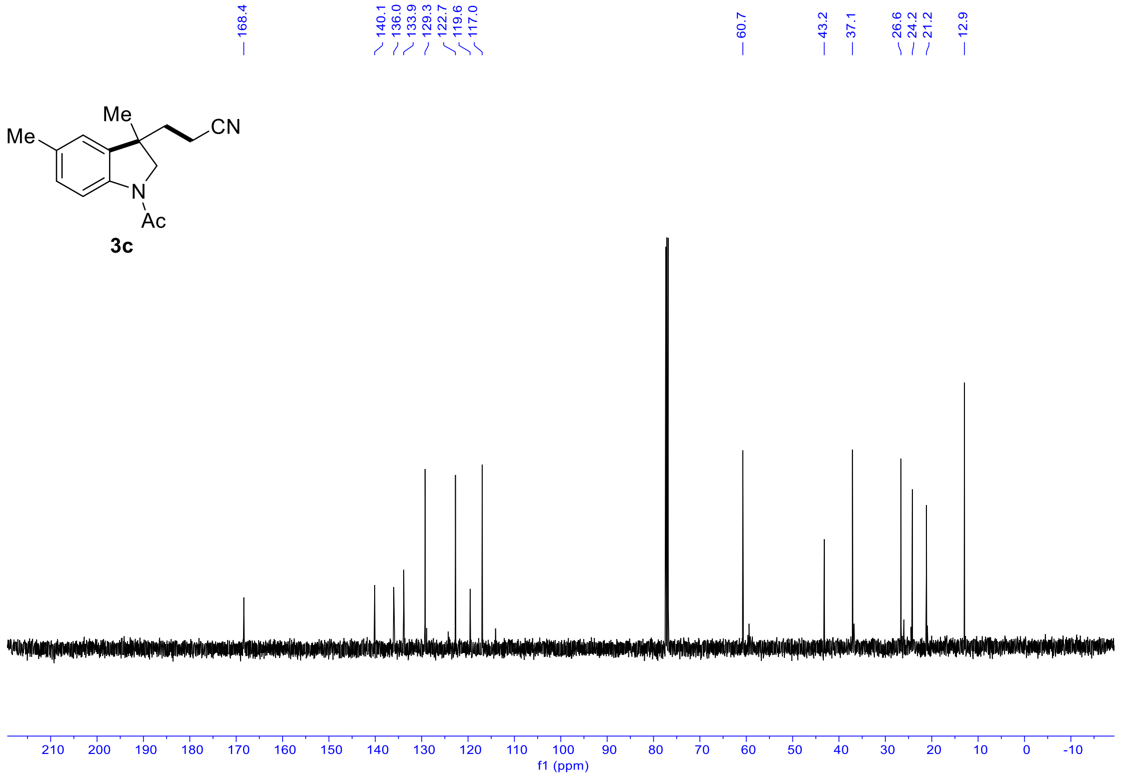

Figure S8: ^{13}C NMR of **3c**, ($CDCl_3$, 126 MHz)

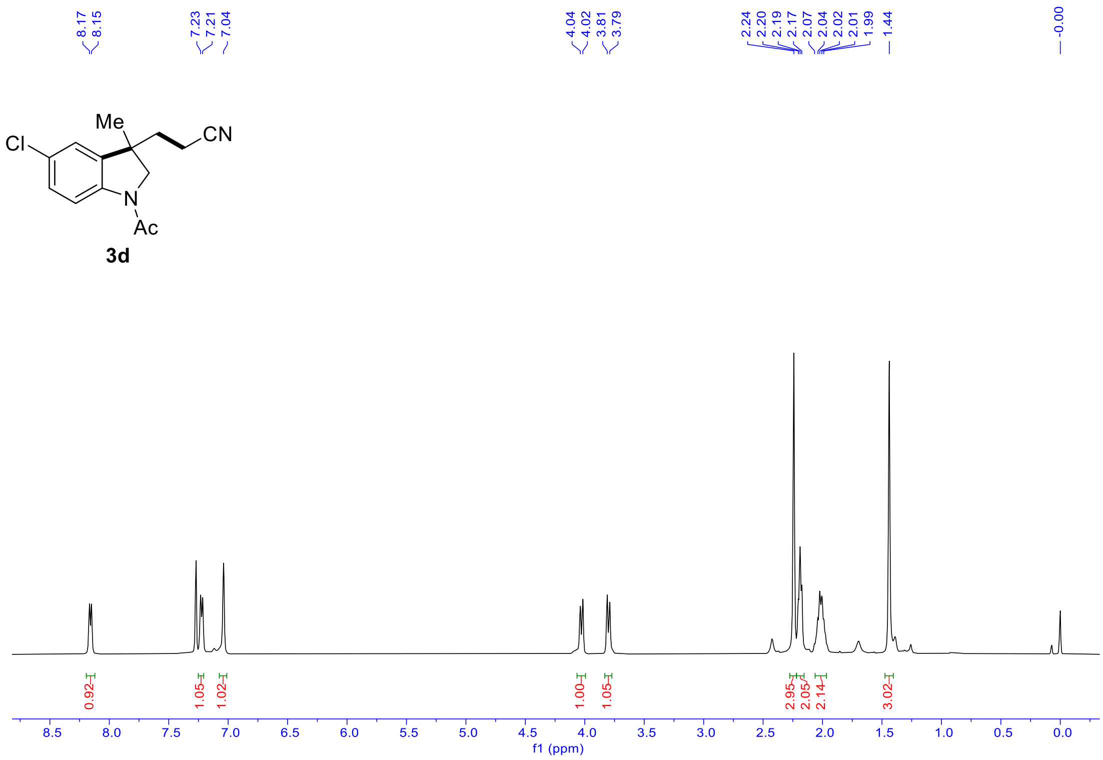

Figure S9: ^{1}H NMR of **3d**, ($CDCl_3$, 500 MHz)

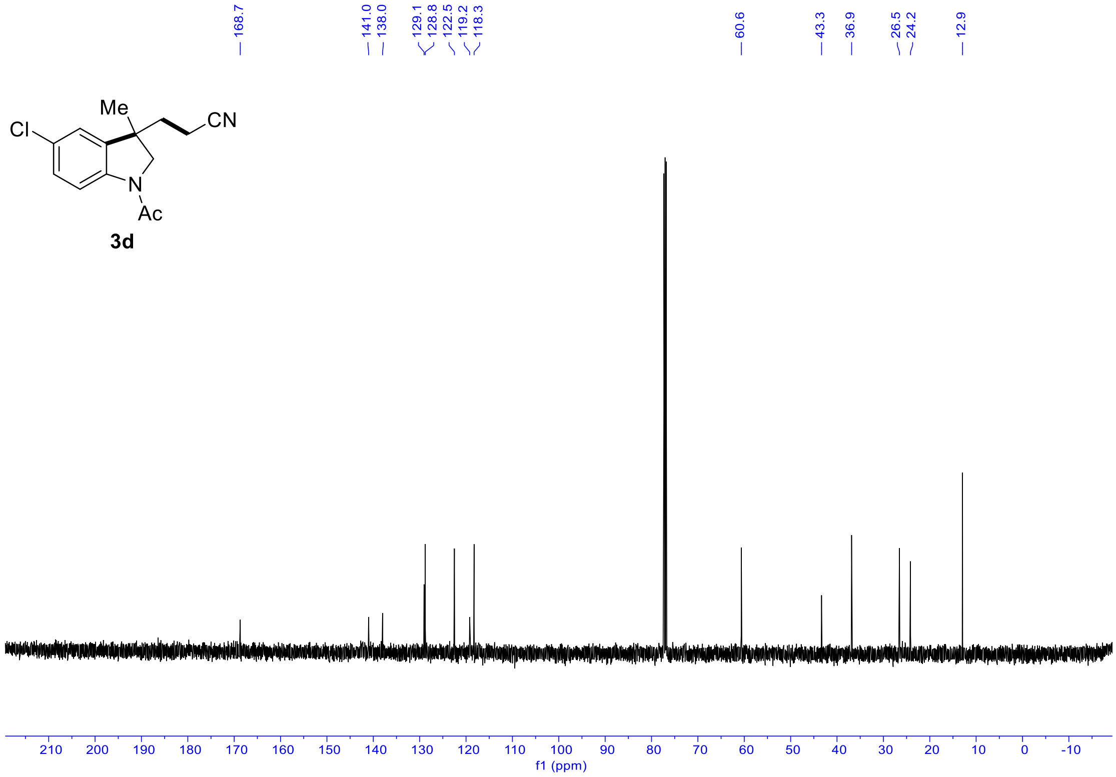

Figure S10: ^{13}C NMR of **3d**, ($CDCl_3$, 126 MHz)

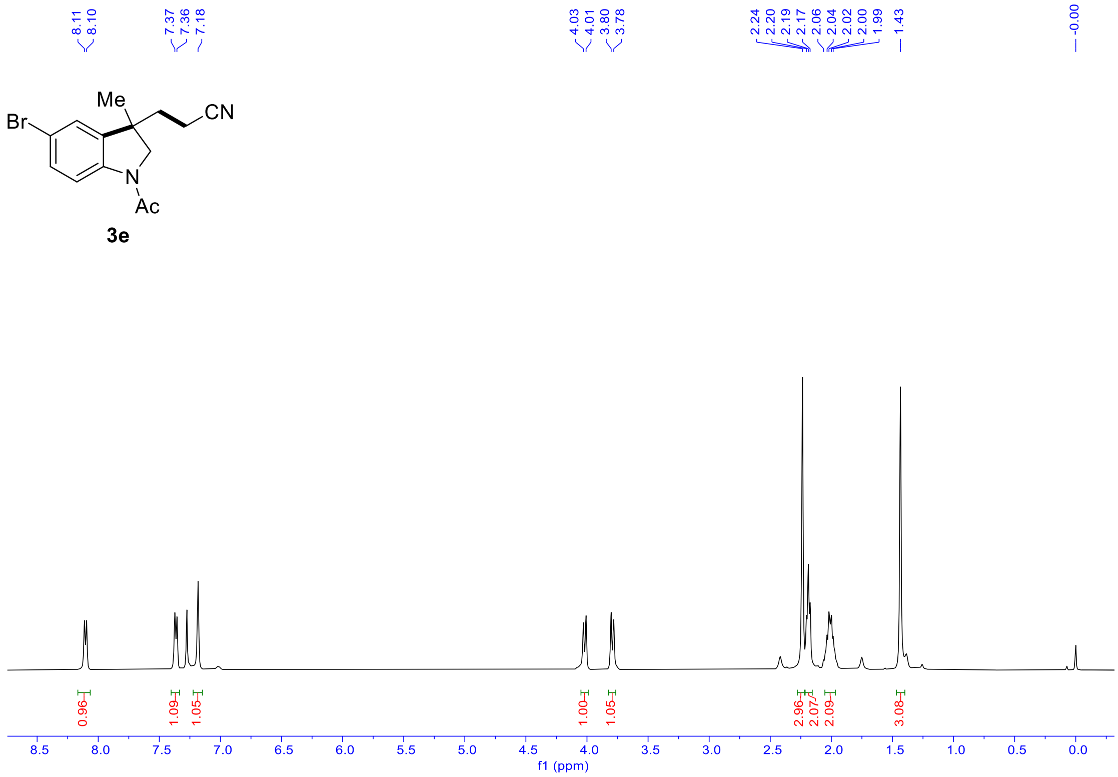

Figure S11: ^{1}H NMR of **3e**, ($CDCl_3$, 500 MHz)

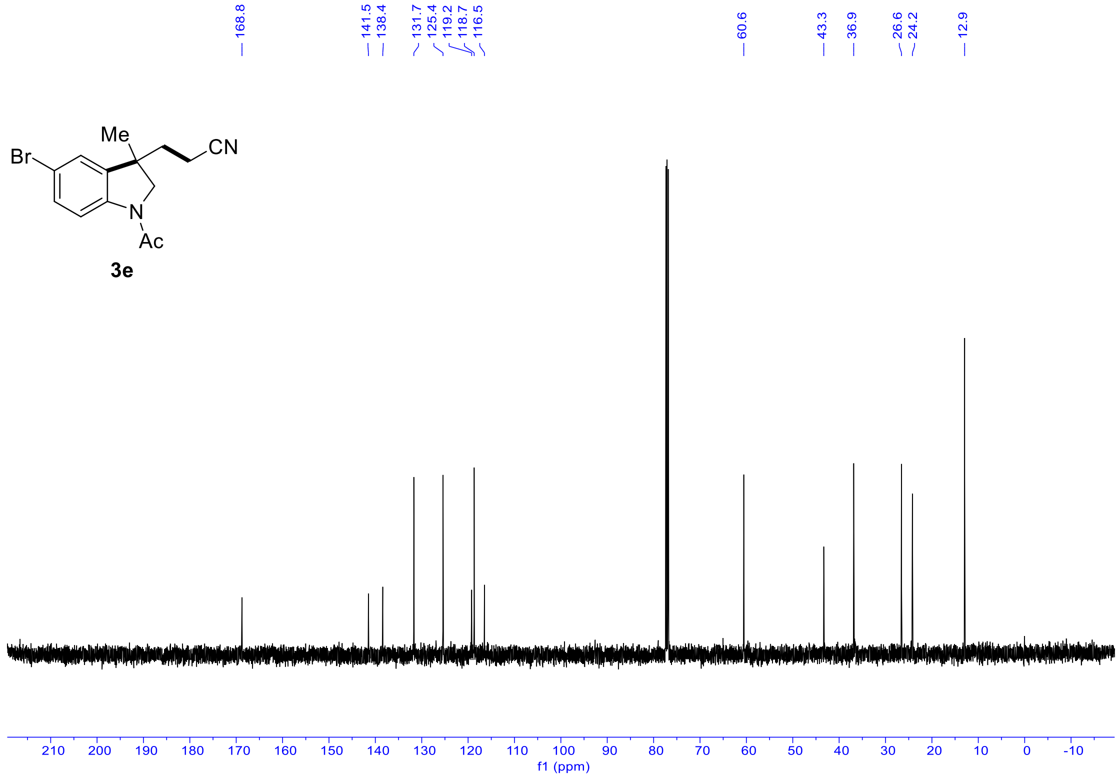

Figure S12: ^{13}C NMR of **3e**, ($CDCl_3$, 126 MHz)

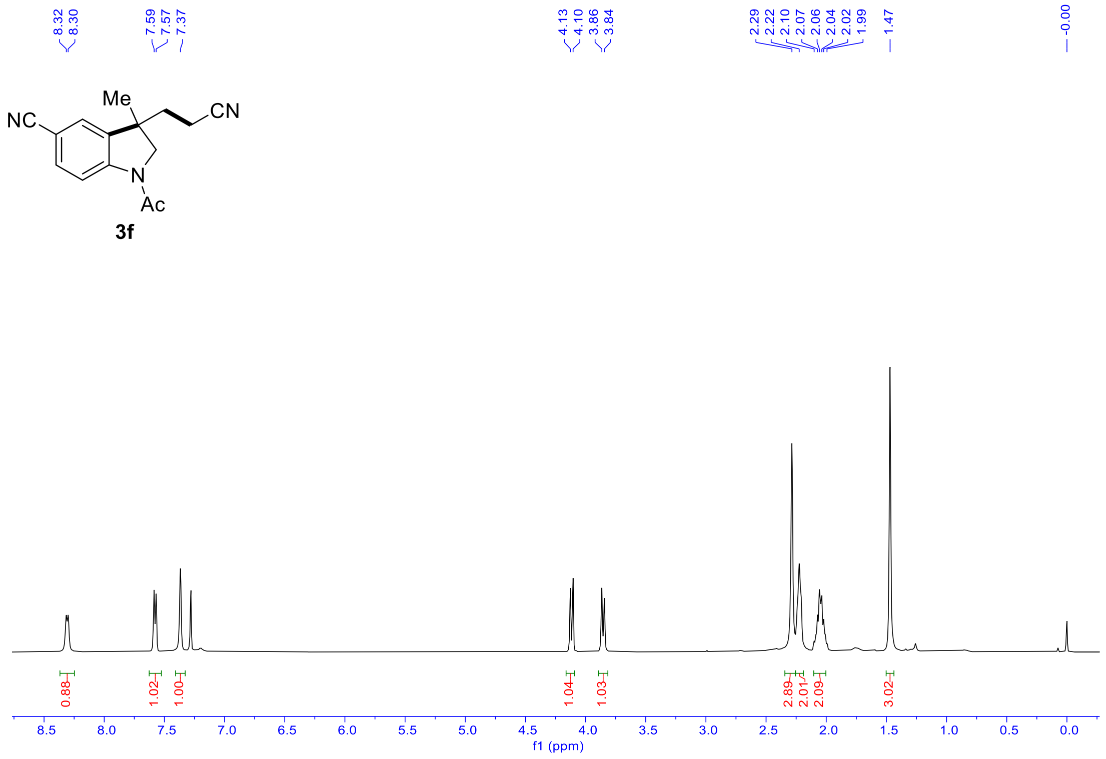

Figure S13: ^{1}H NMR of **3f**, ($CDCl_3$, 500 MHz)

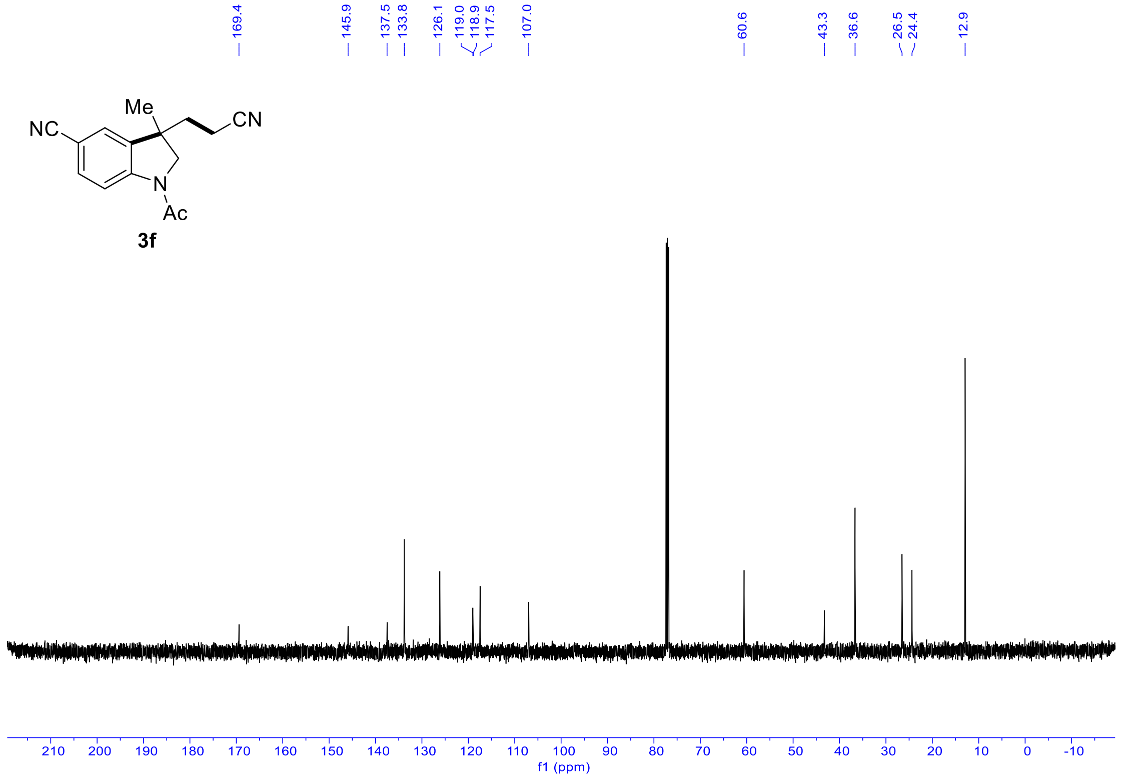

Figure S14: ^{13}C NMR of **3f**, ($CDCl_3$, 126 MHz)

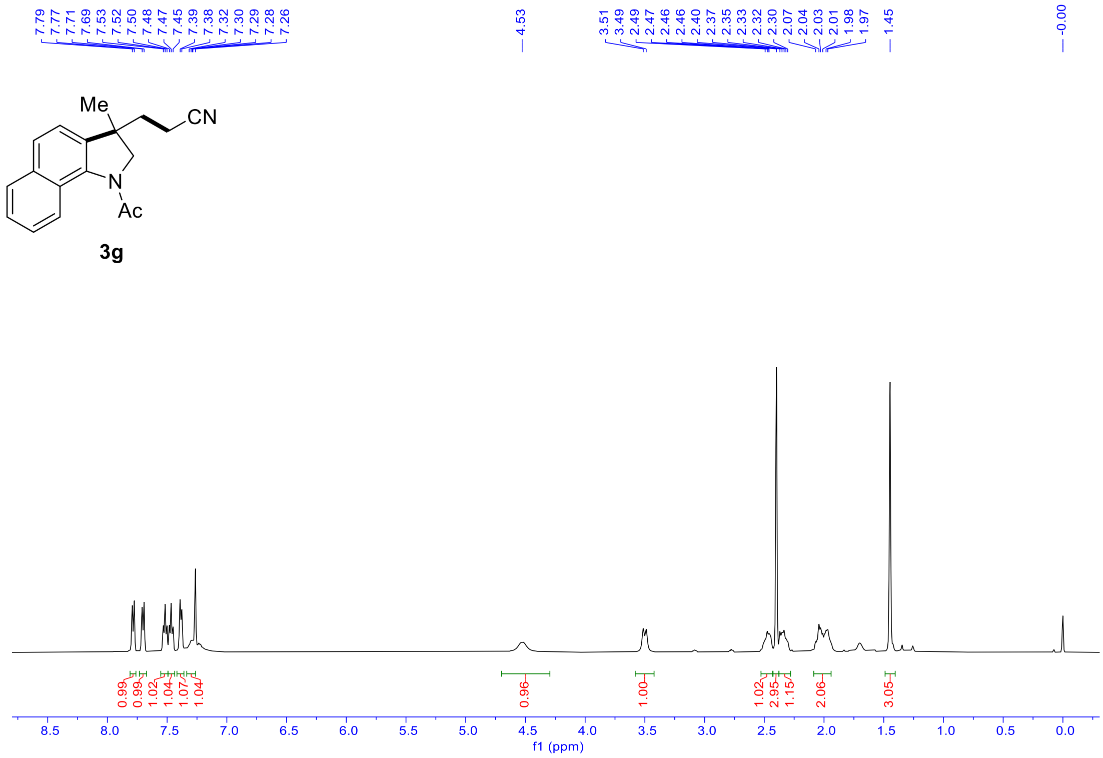

Figure S15: ^{1}H NMR of **3g**, ($CDCl_3$, 500 MHz)

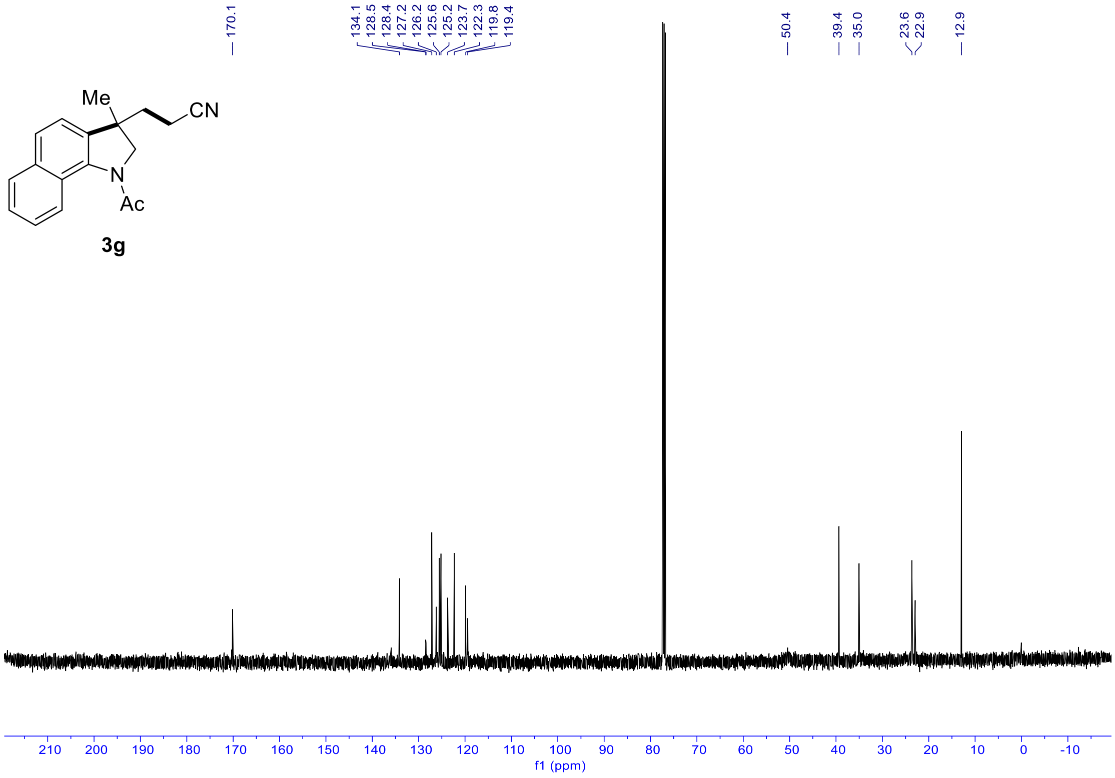

Figure S16: ^{13}C NMR of **3g**, ($CDCl_3$, 126 MHz)

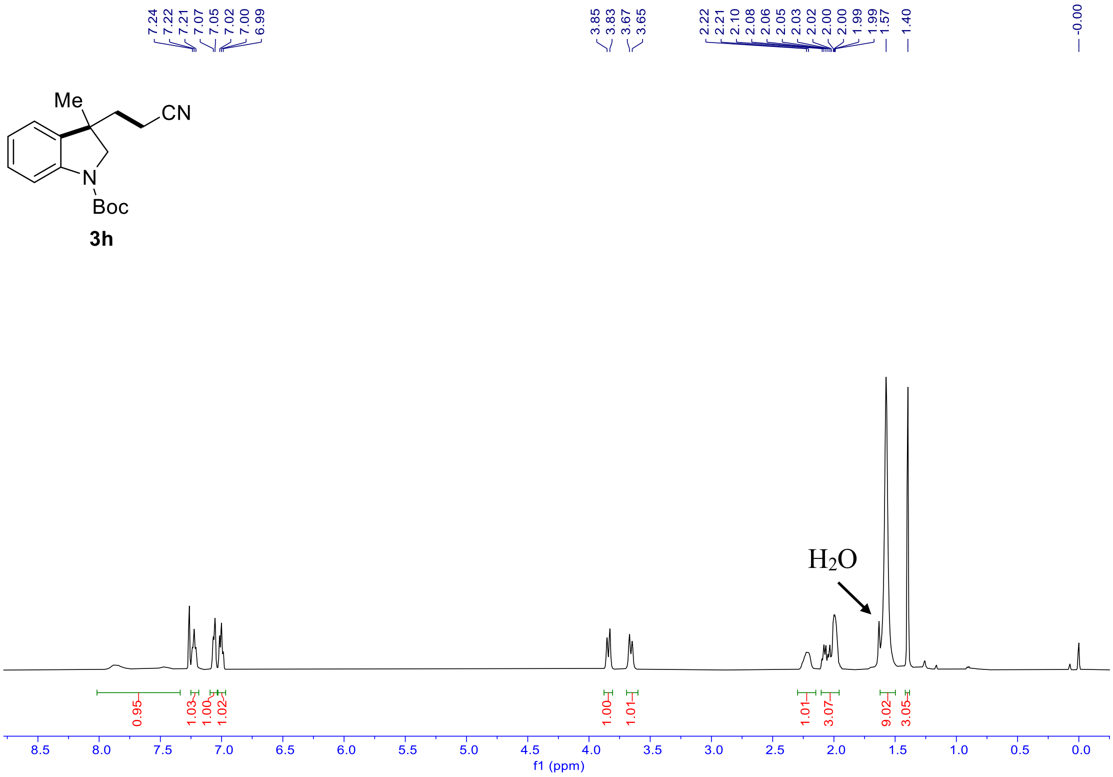

Figure S17: ^{1}H NMR of **3h**, ($CDCl_3$, 500 MHz)

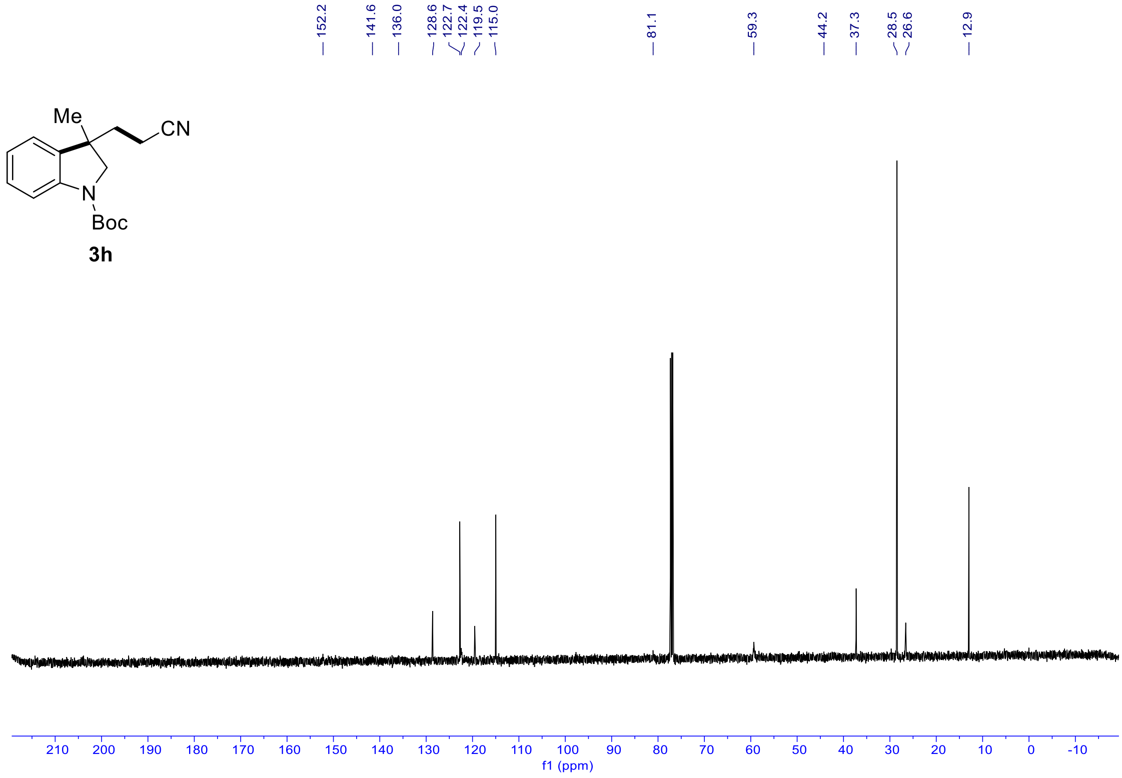

Figure S18: ^{13}C NMR of **3h**, ($CDCl_3$, 126 MHz)

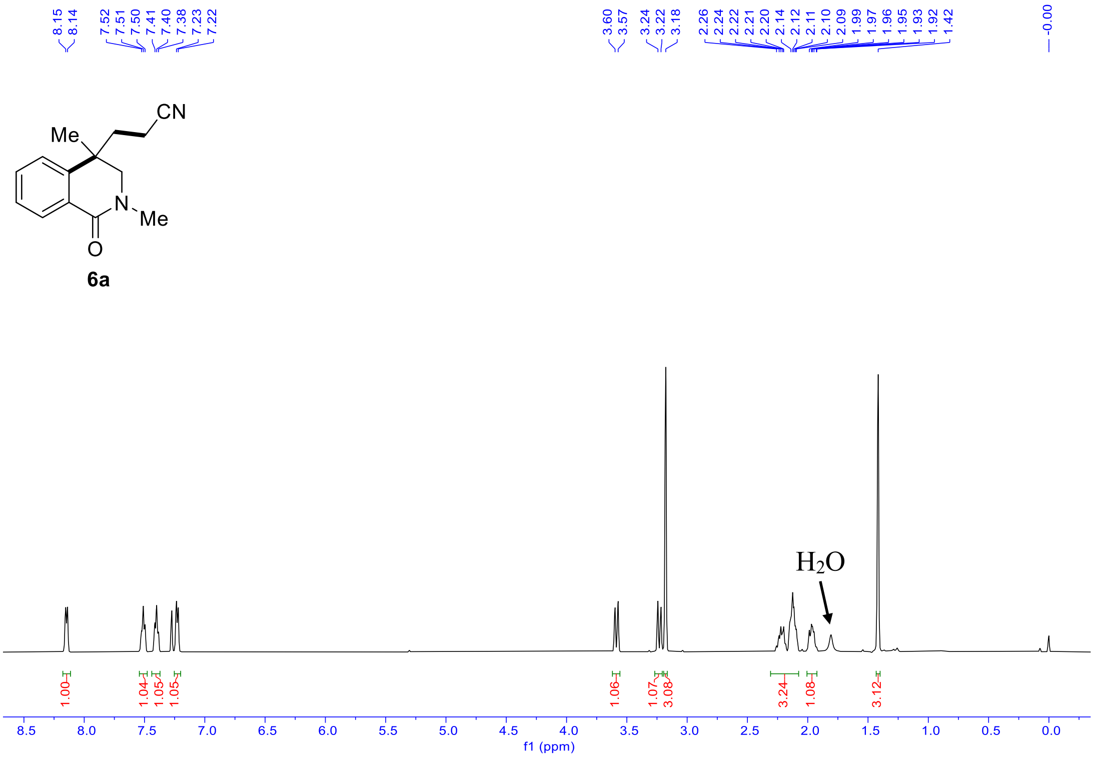

Figure S19: ^{1}H NMR of **6a**, ($CDCl_3$, 500 MHz)

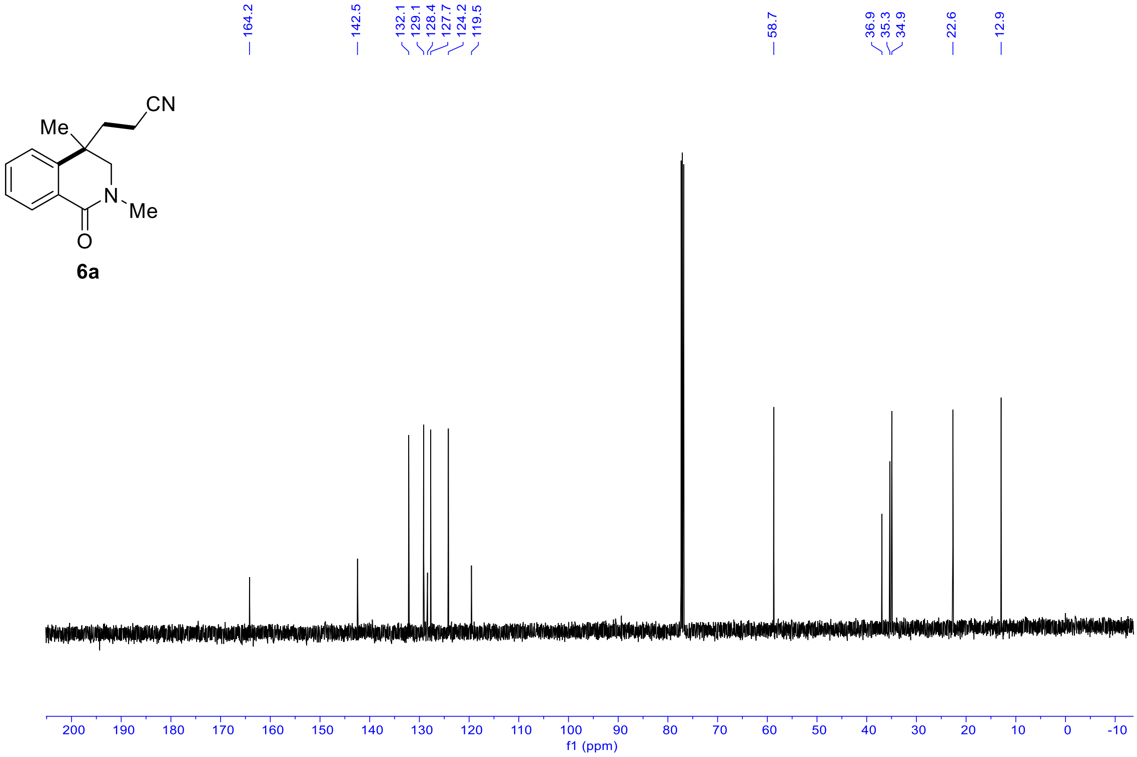

Figure S20: ^{13}C NMR of **6a**, ($CDCl_3$, 126 MHz)

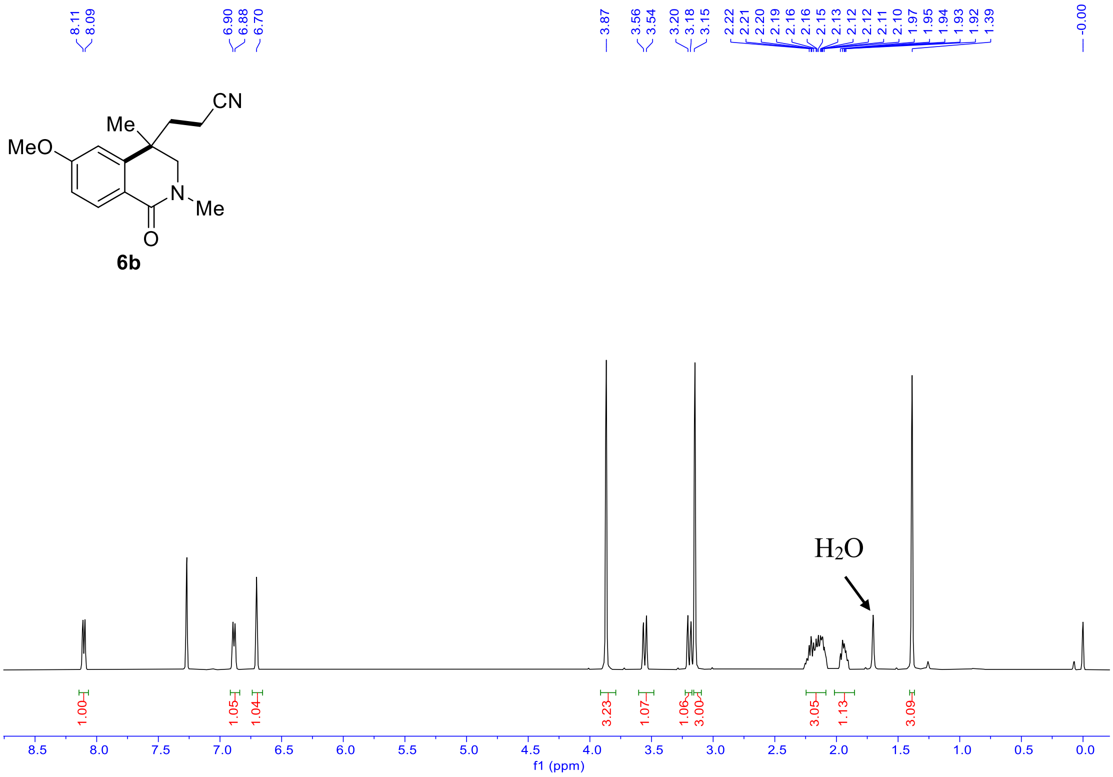

Figure S21: ^{1}H NMR of **6b**, ($CDCl_3$, 500 MHz)

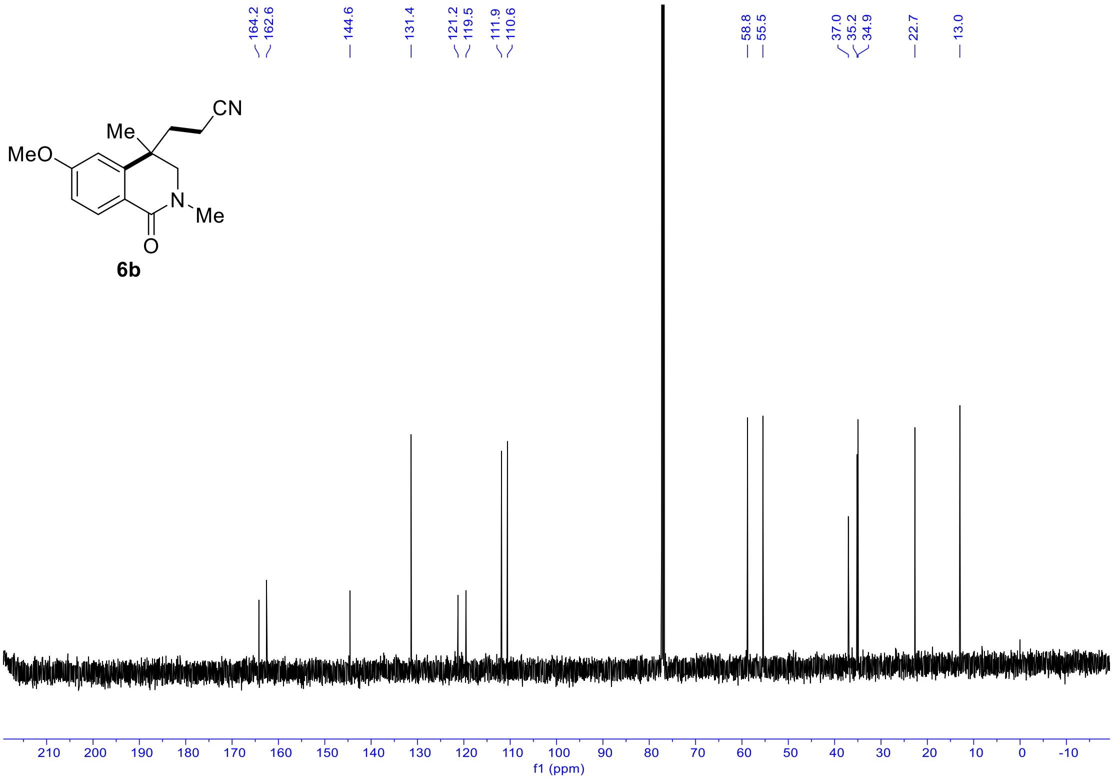

Figure S22: ^{13}C NMR of **6b**, ($CDCl_3$, 126 MHz)

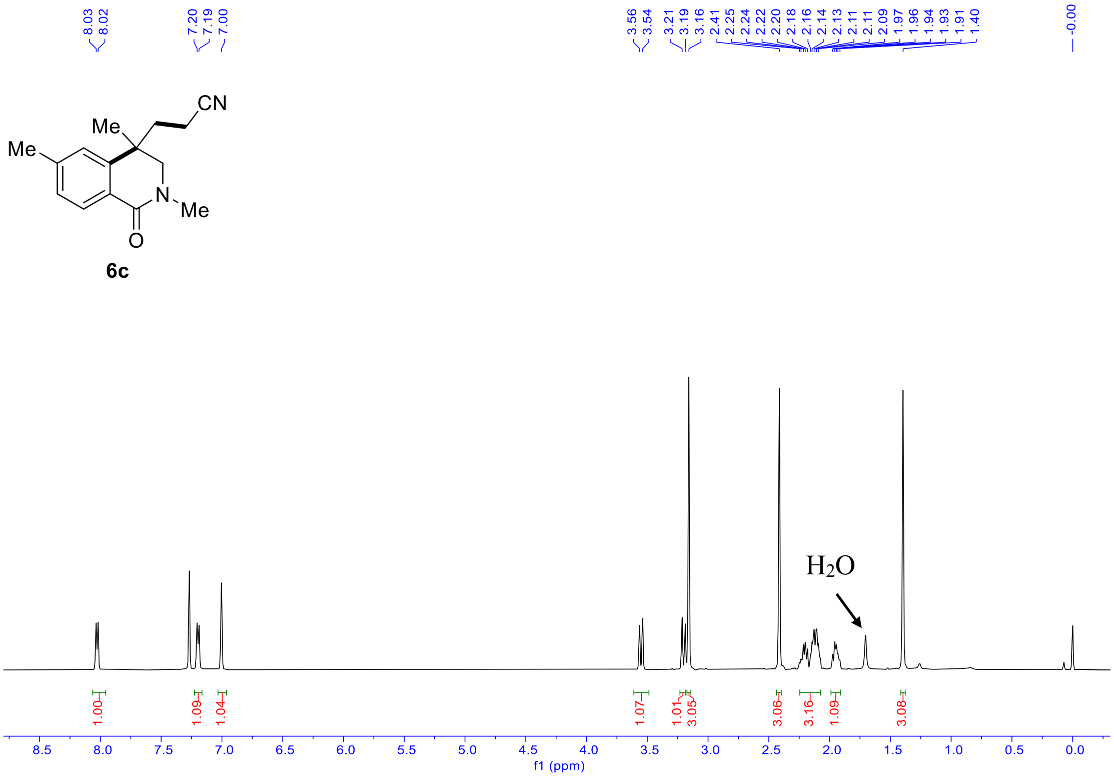

Figure S23: ^{1}H NMR of **6c**, ($CDCl_3$, 500 MHz)

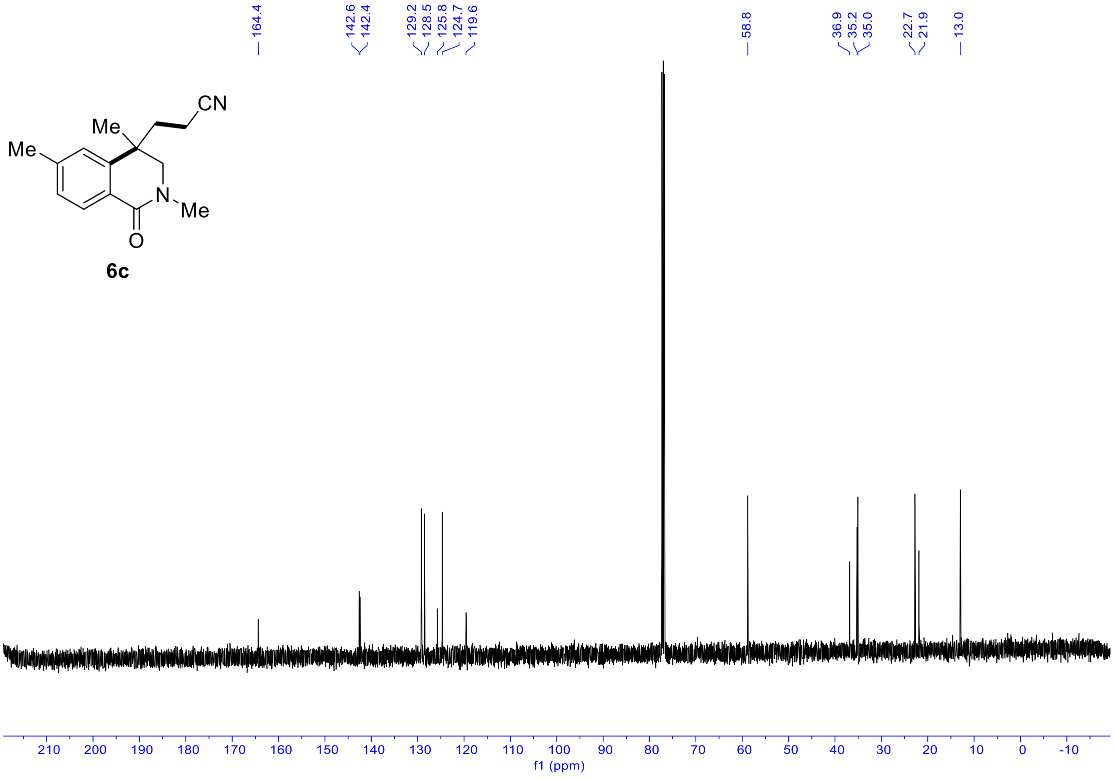

Figure S24: ^{13}C NMR of **6c**, ($CDCl_3$, 126 MHz)

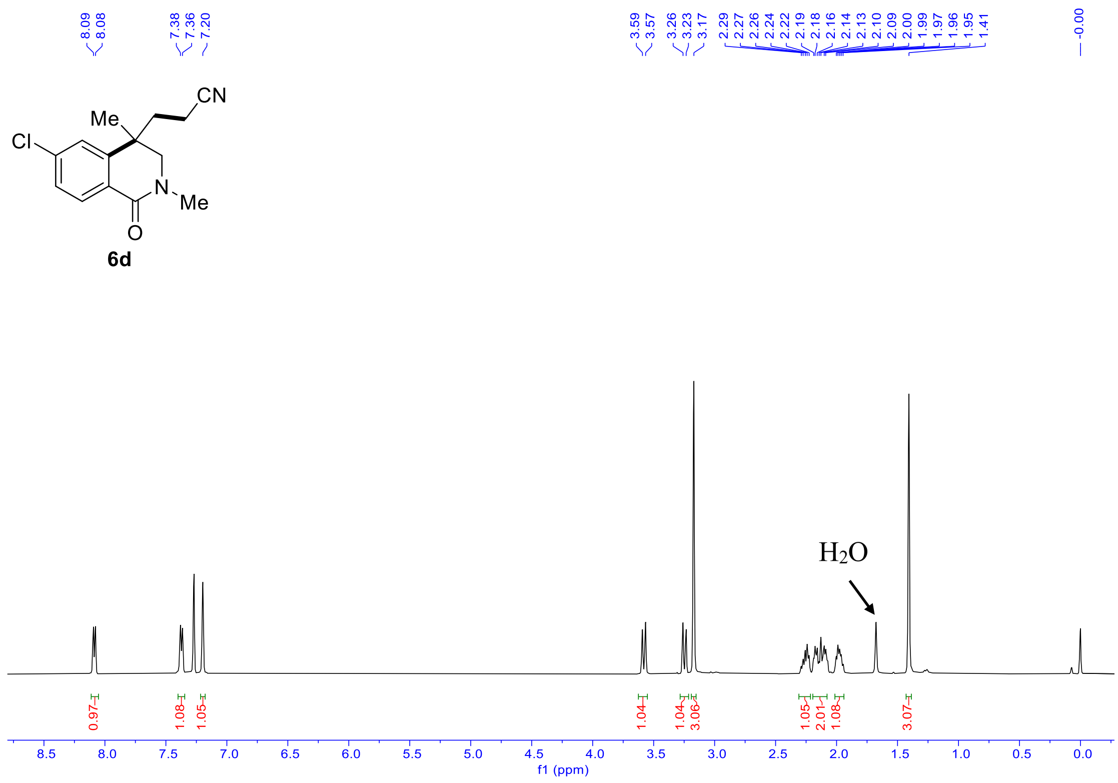

Figure S25: ^{1}H NMR of **6d**, ($CDCl_3$, 500 MHz)

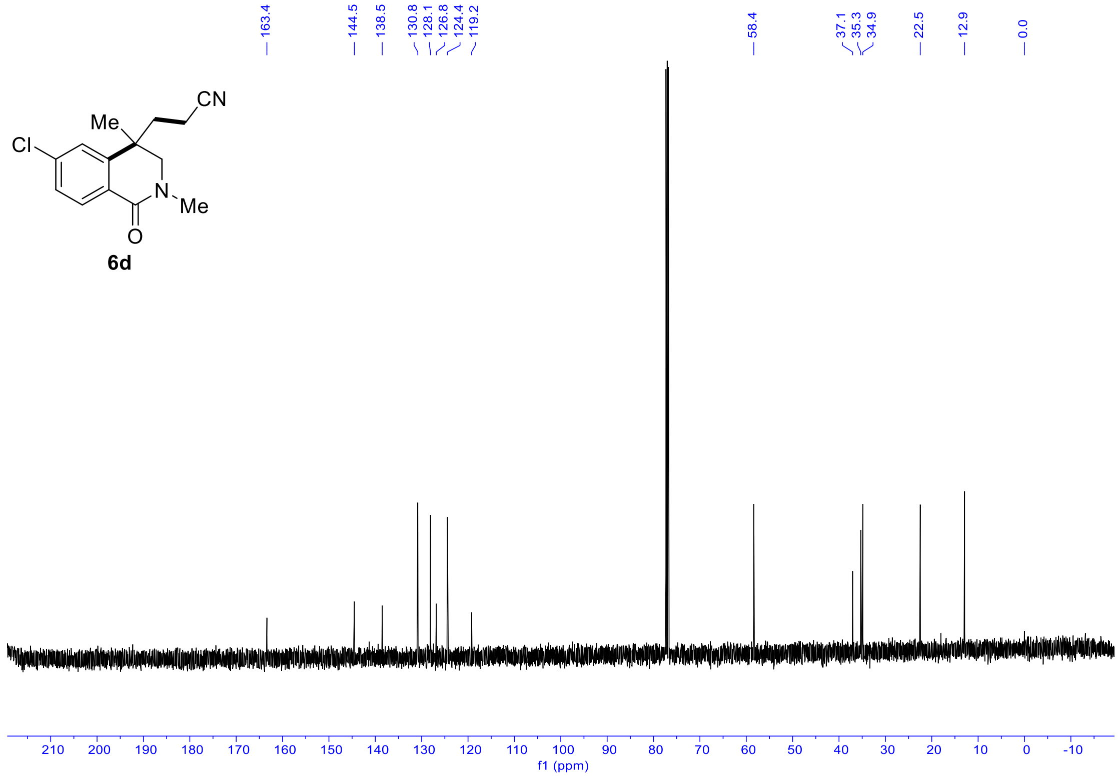

Figure S26: ^{13}C NMR of **6d**, ($CDCl_3$, 126 MHz)

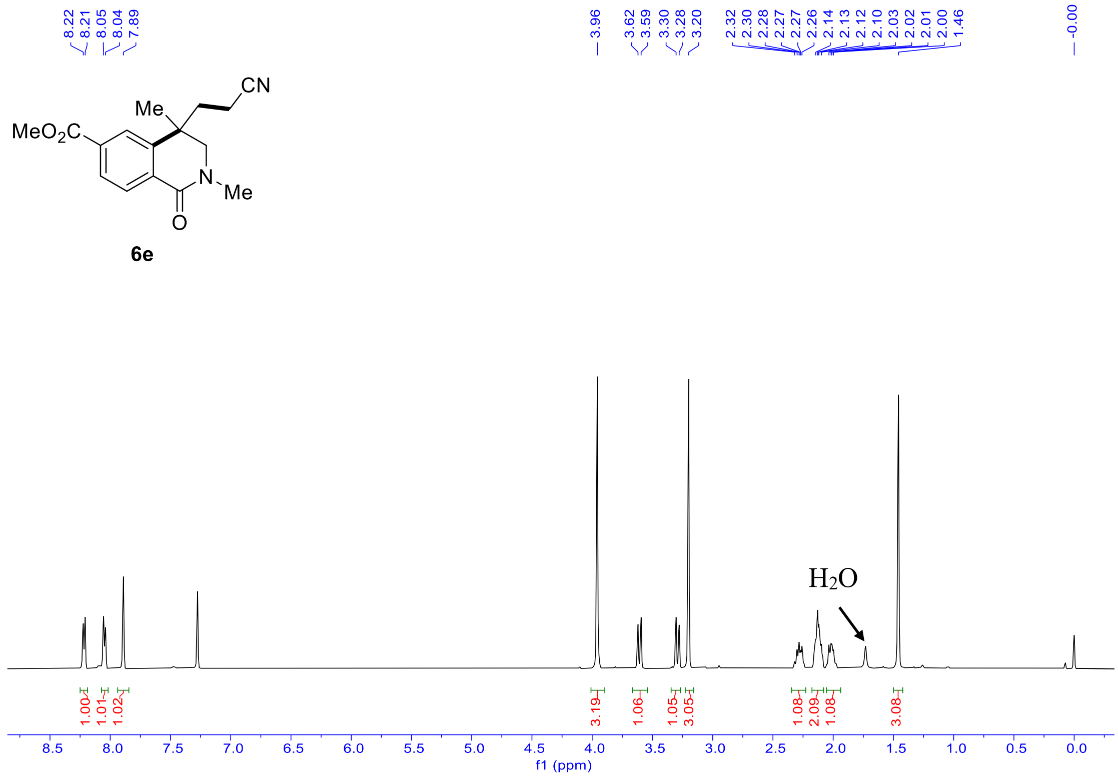

Figure S27: ^{1}H NMR of **6e**, ($CDCl_3$, 500 MHz)

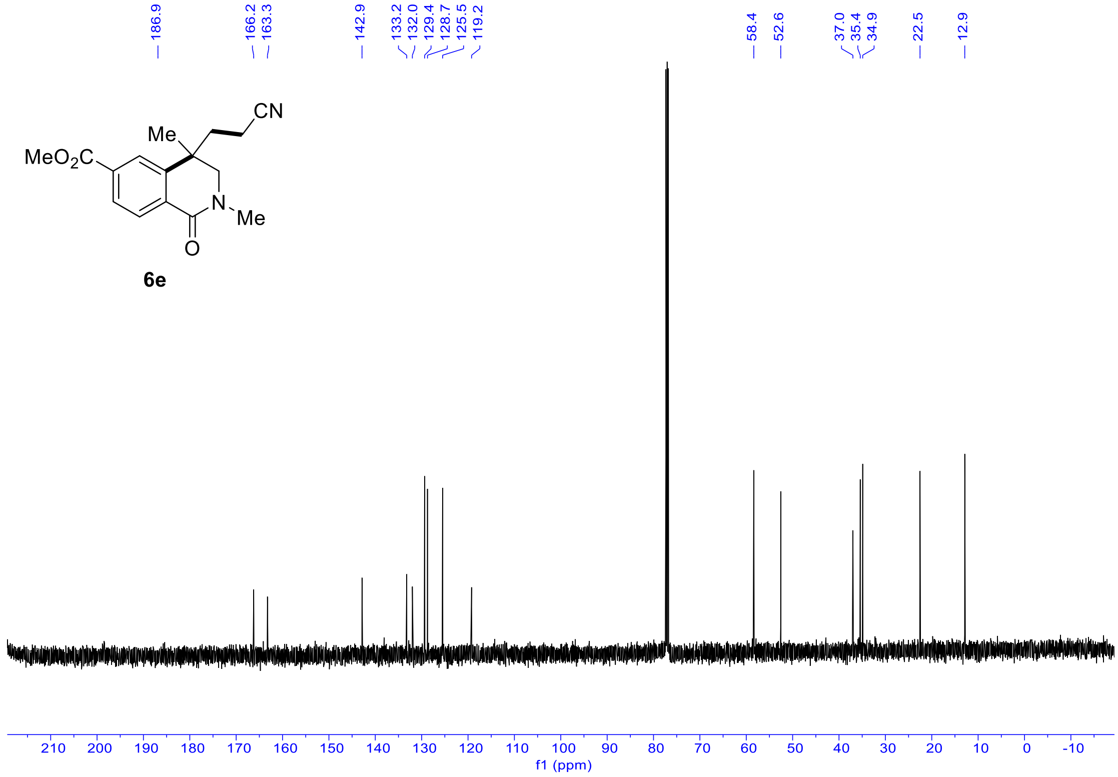

Figure S28: ^{13}C NMR of **6e**, ($CDCl_3$, 126 MHz)

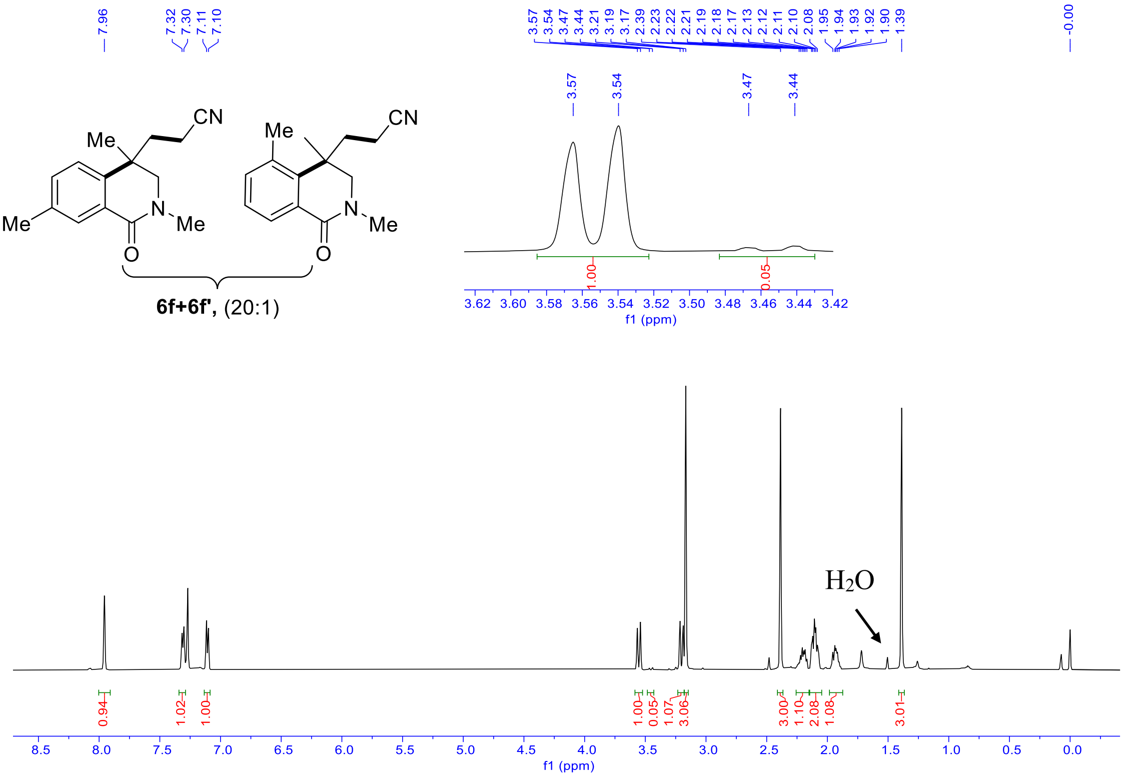

Figure S29: ^{1}H NMR of **6f+6f'**, ($CDCl_3$, 126 MHz)

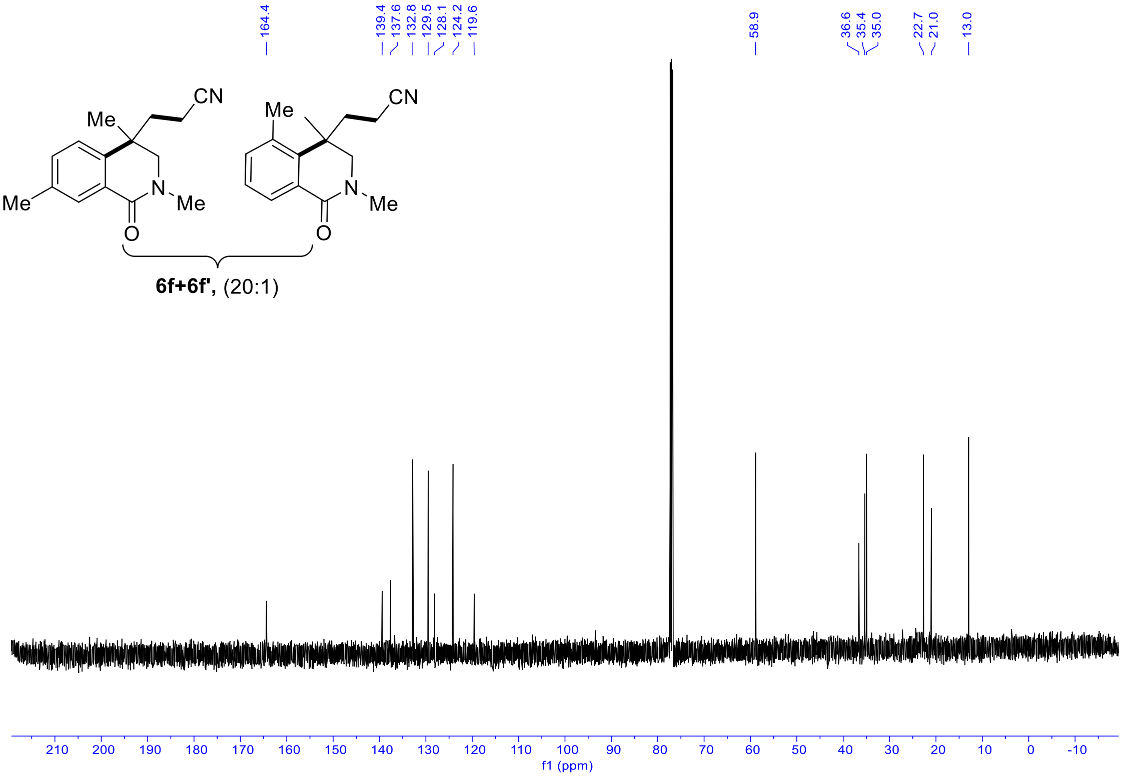

Figure S30: ^{13}C NMR of **6f+6f'**, ($CDCl_3$, 126 MHz)

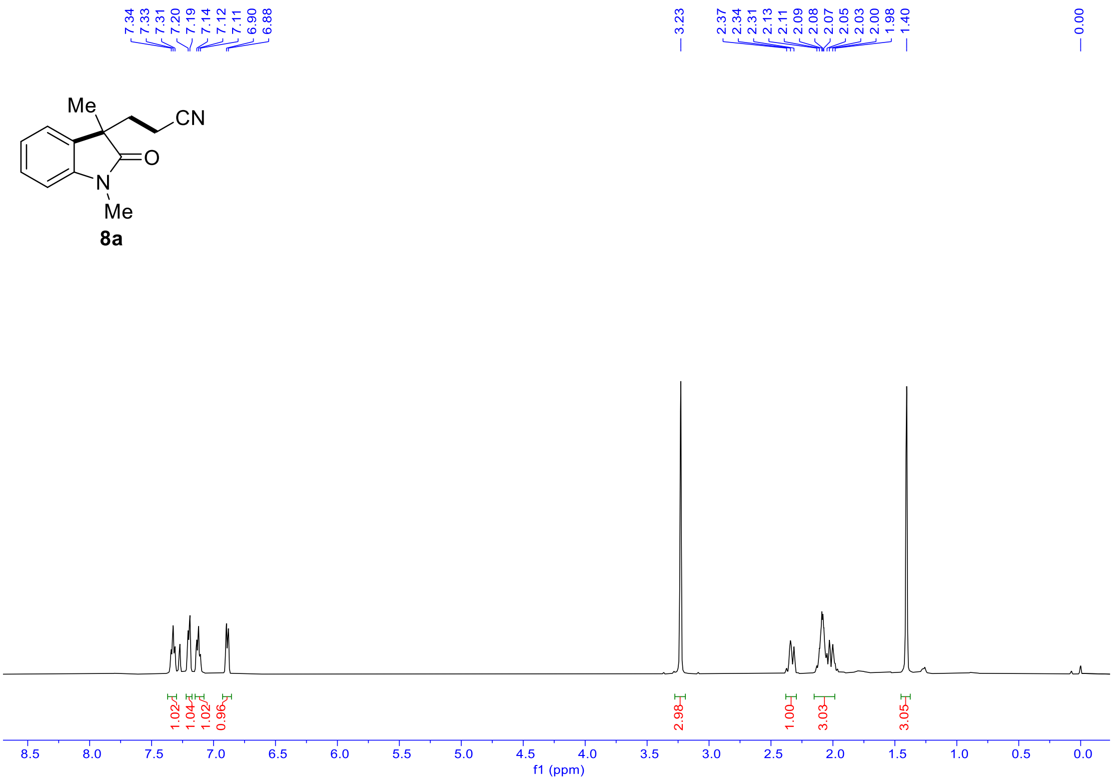

Figure S31: ^{1}H NMR of **8a**, ($CDCl_3$, 500 MHz)

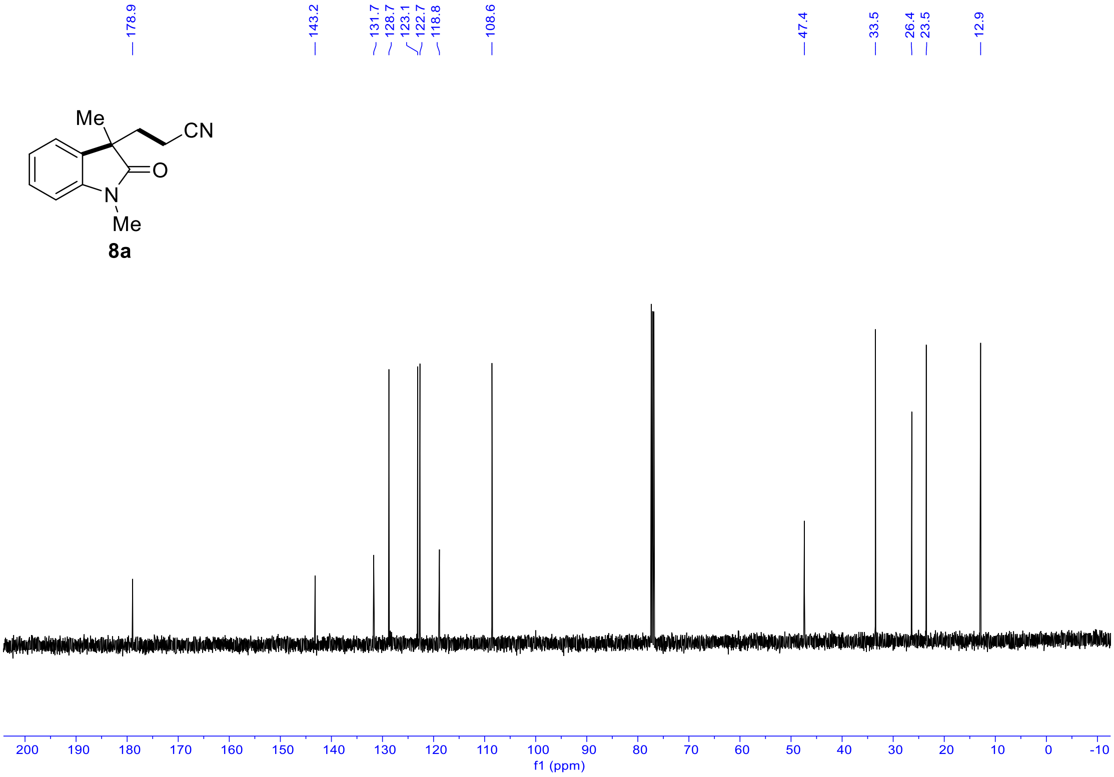

Figure S32: ^{13}C NMR of **8a**, ($CDCl_3$, 126 MHz)

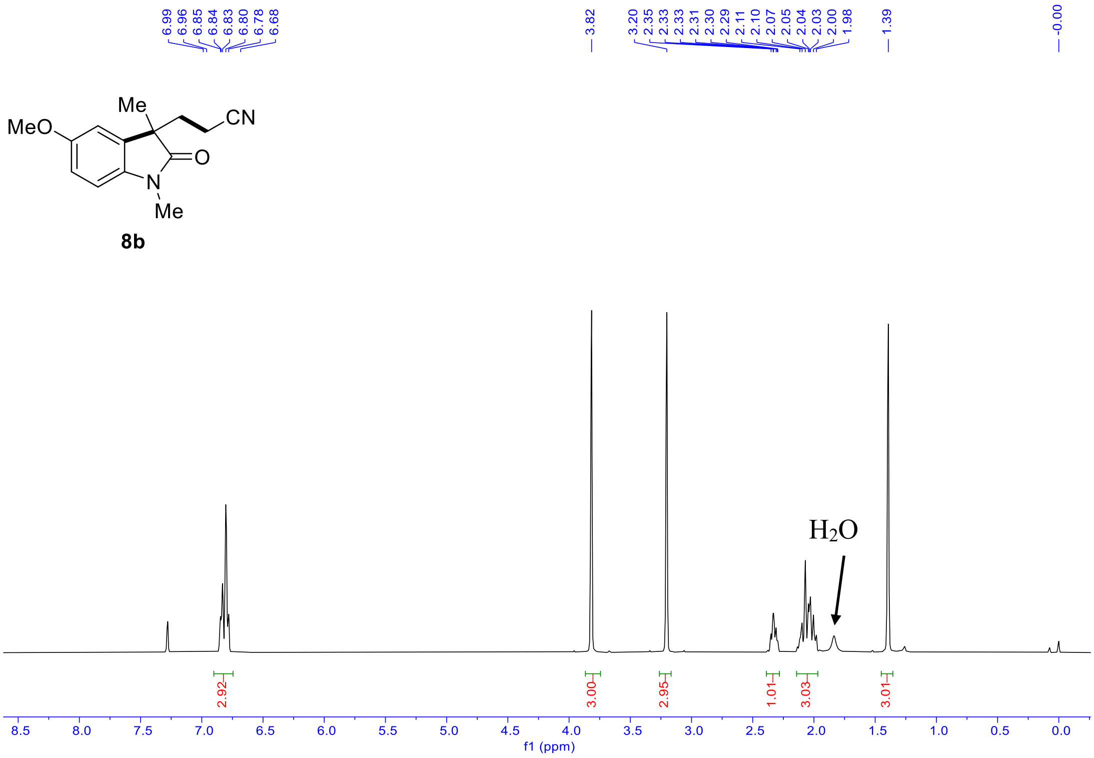

Figure S33: ^{1}H NMR of **8b**, ($CDCl_3$, 500 MHz)

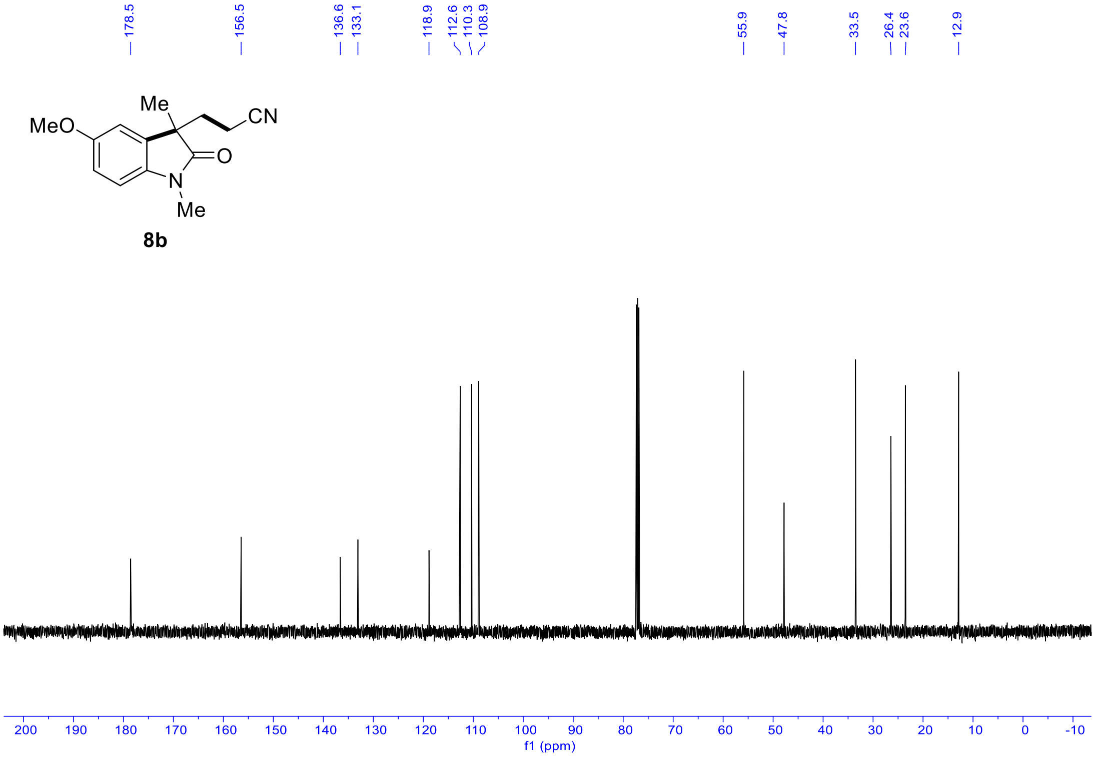

Figure S34: ^{13}C NMR of **8b**, ($CDCl_3$, 126 MHz)

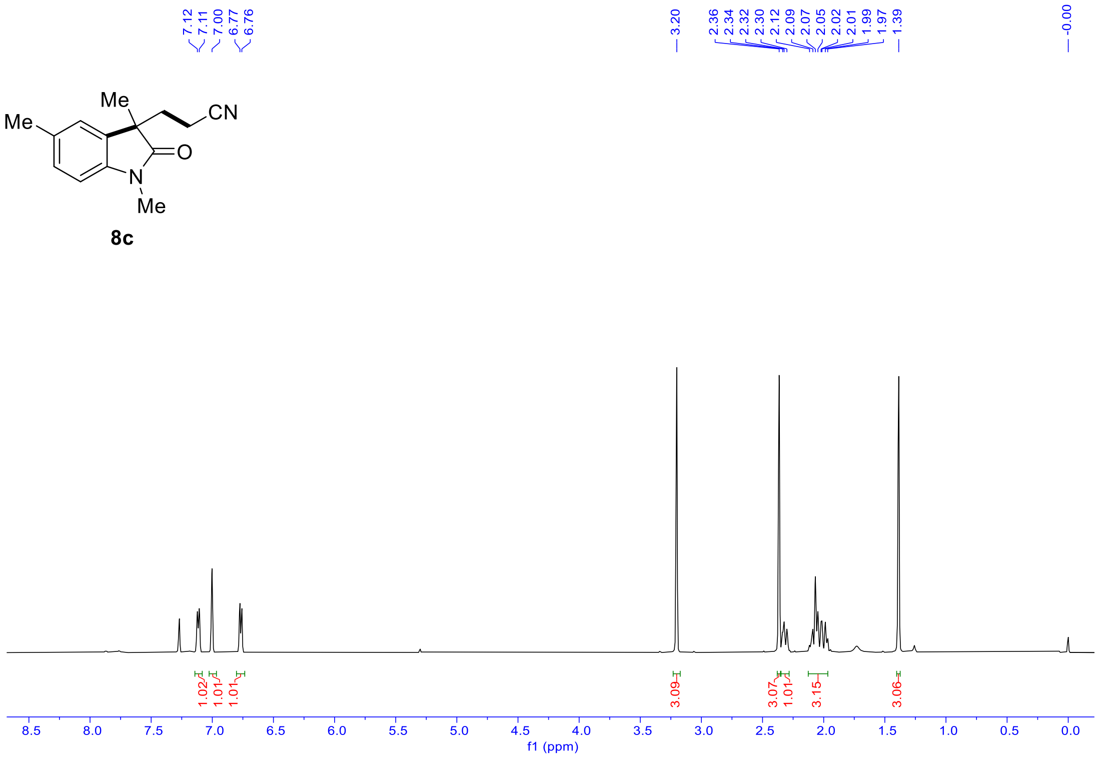

Figure S35: ^{1}H NMR of **8c**, ($CDCl_3$, 500 MHz)

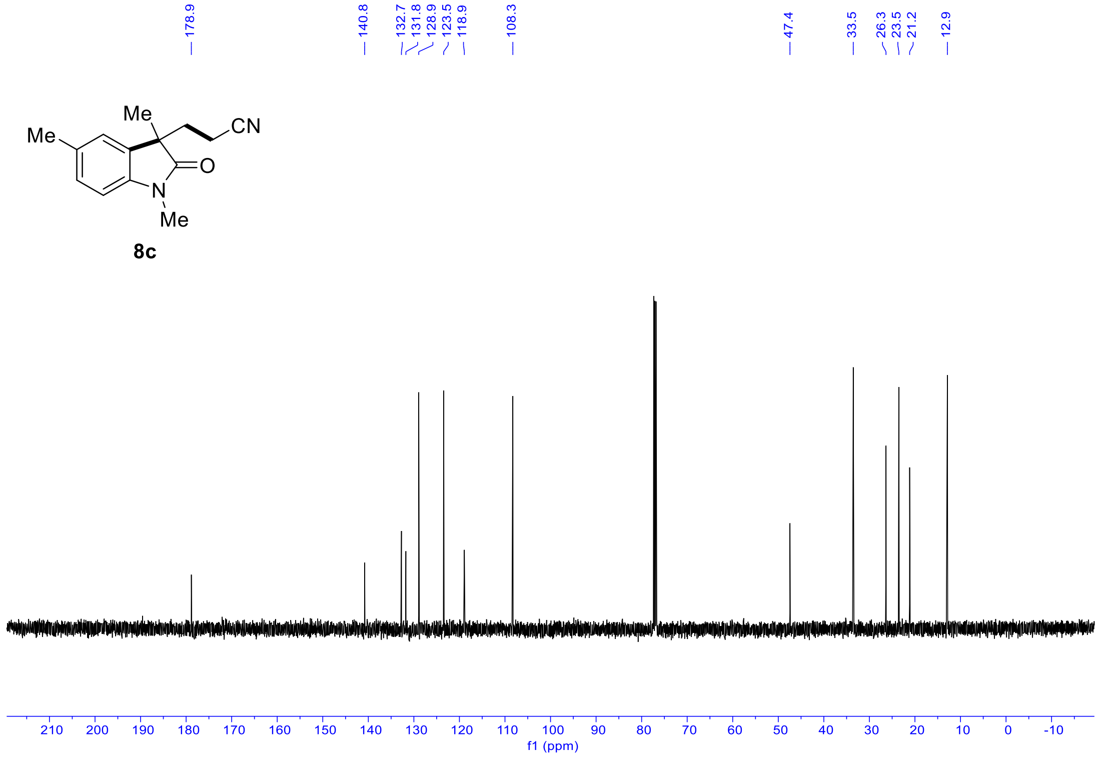

Figure S36: ^{13}C NMR of **8c**, ($CDCl_3$, 126 MHz)

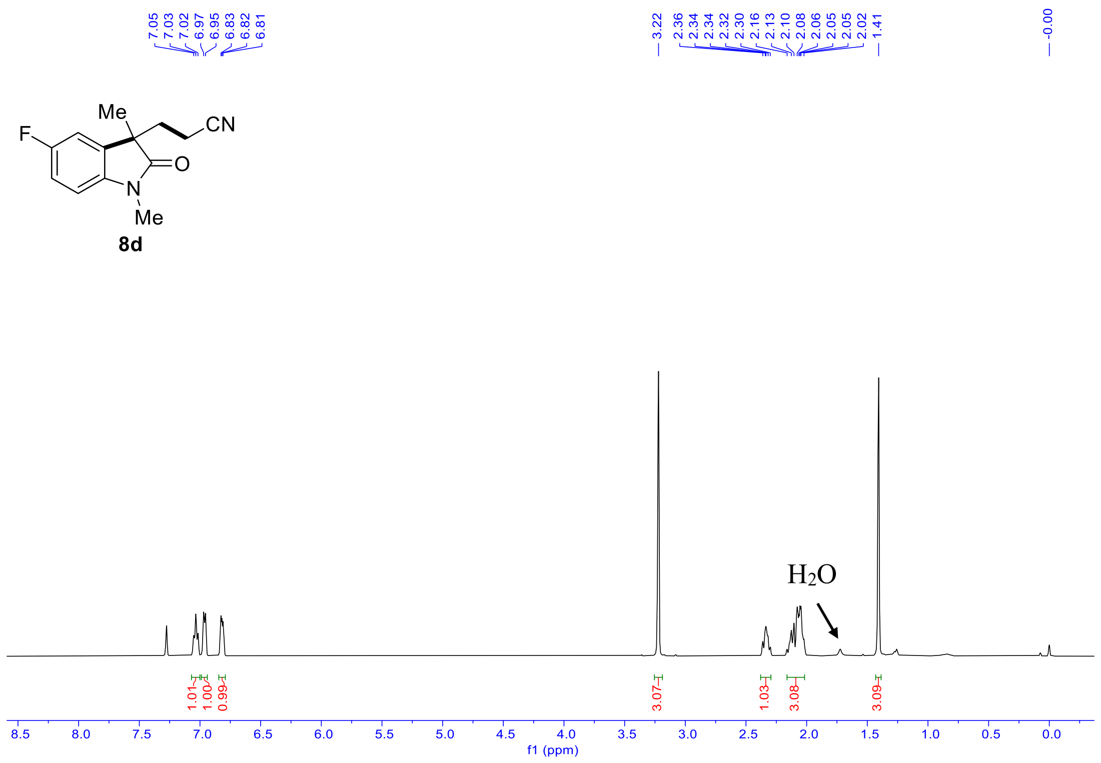

Figure S37: ^{1}H NMR of **8d**, ($CDCl_3$, 500 MHz)

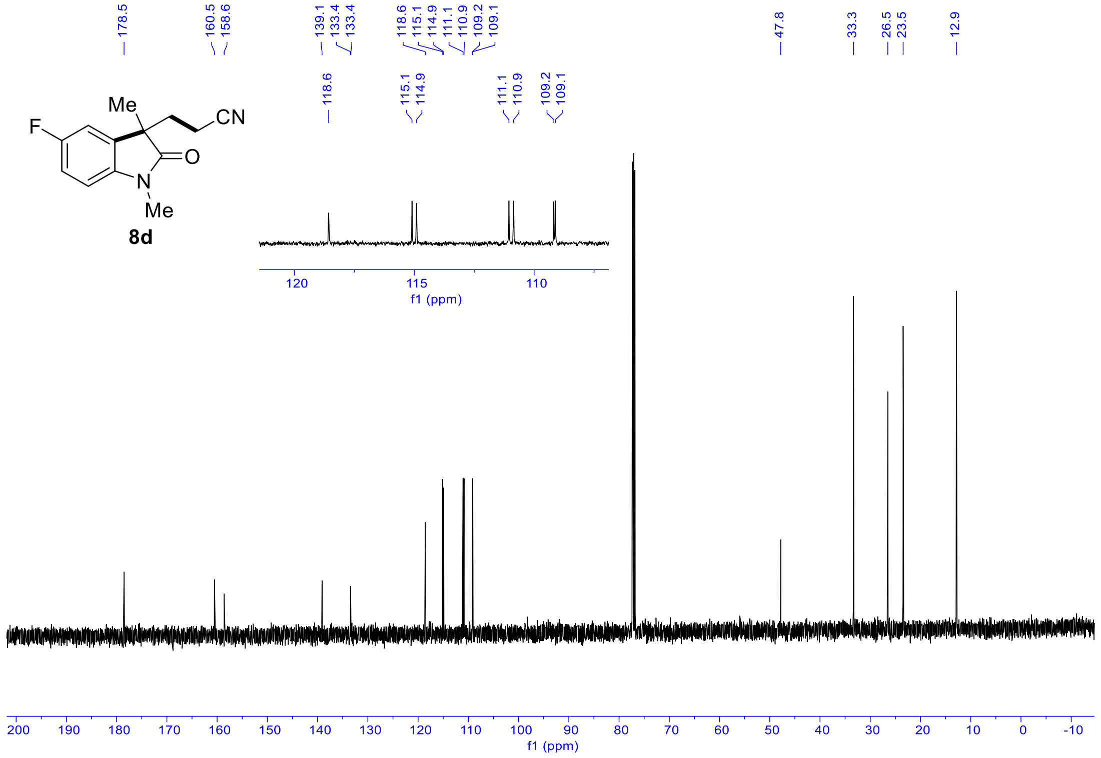

Figure S38: ^{13}C NMR of **8d**, ($CDCl_3$, 126 MHz)

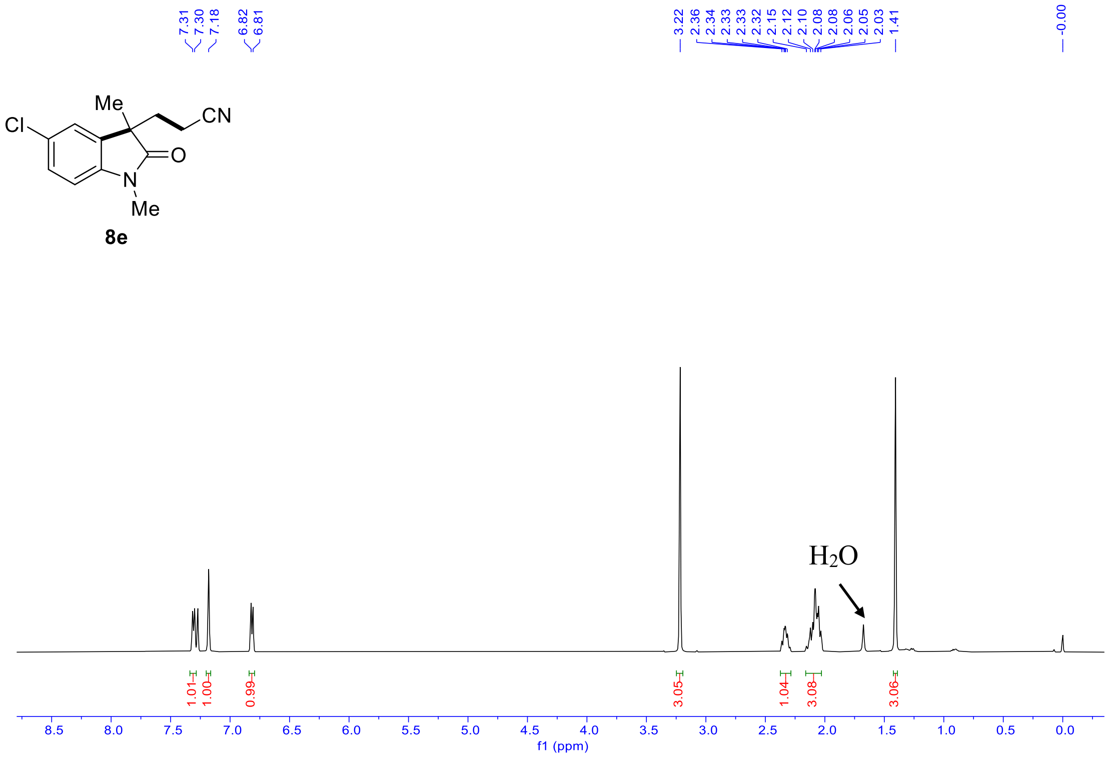

Figure S39: ^{1}H NMR of **8e**, ($CDCl_3$, 500 MHz)

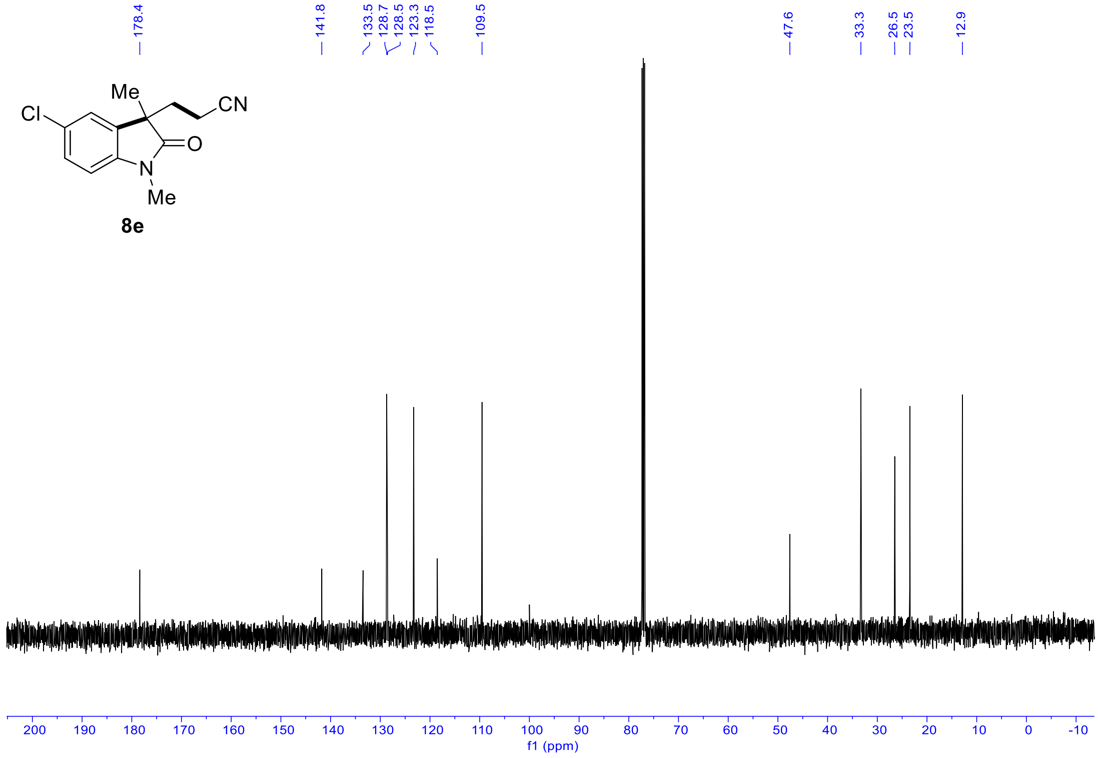

Figure S40: ^{13}C NMR of **8e**, ($CDCl_3$, 126 MHz)

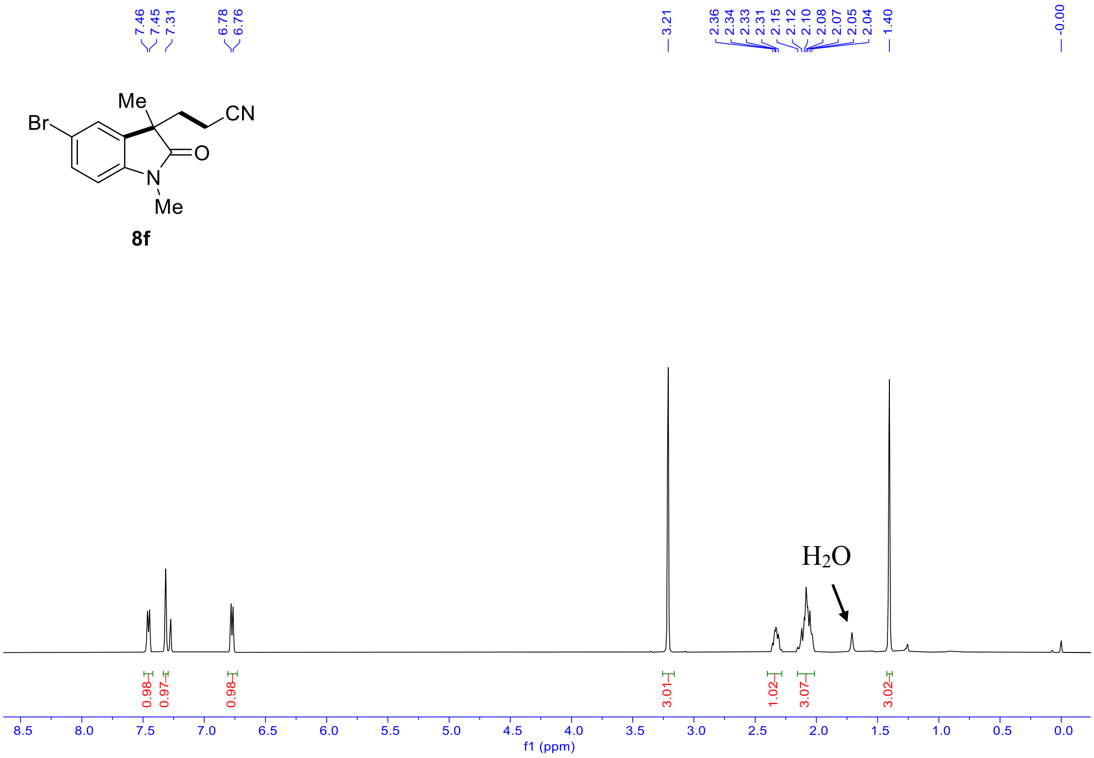

Figure S41: ^{1}H NMR of **8f**, ($CDCl_3$, 500 MHz)

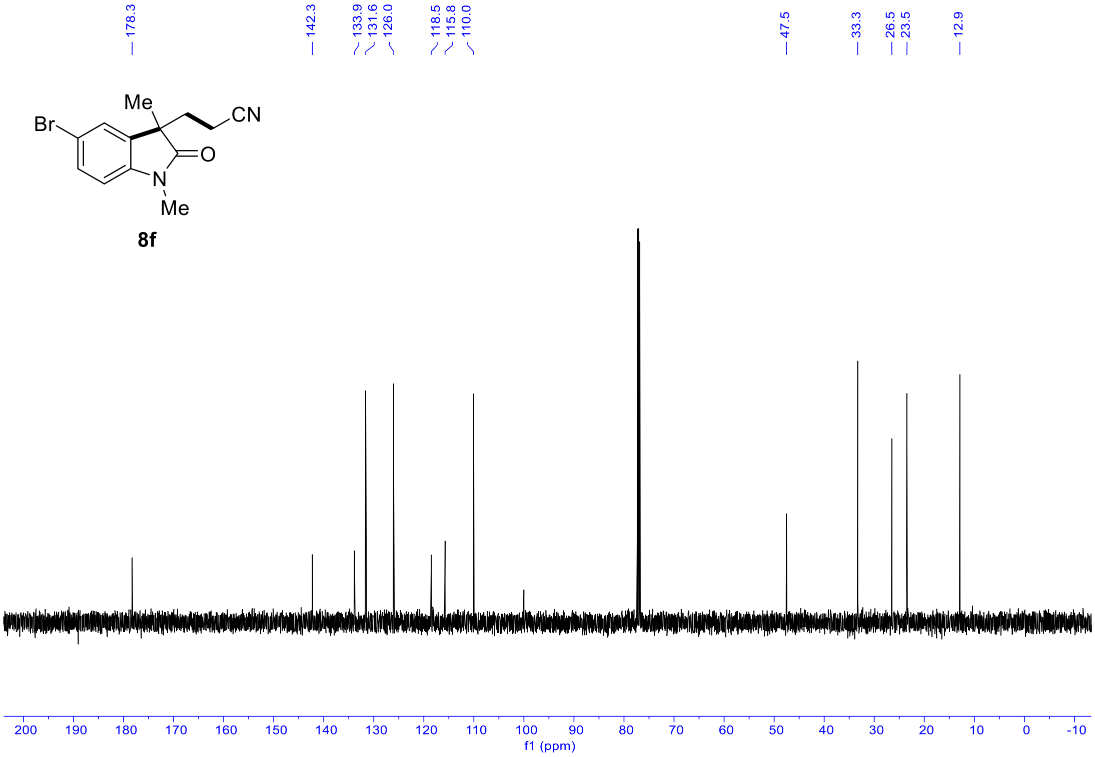

Figure S42: ^{13}C NMR of **8f**, ($CDCl_3$, 126 MHz)

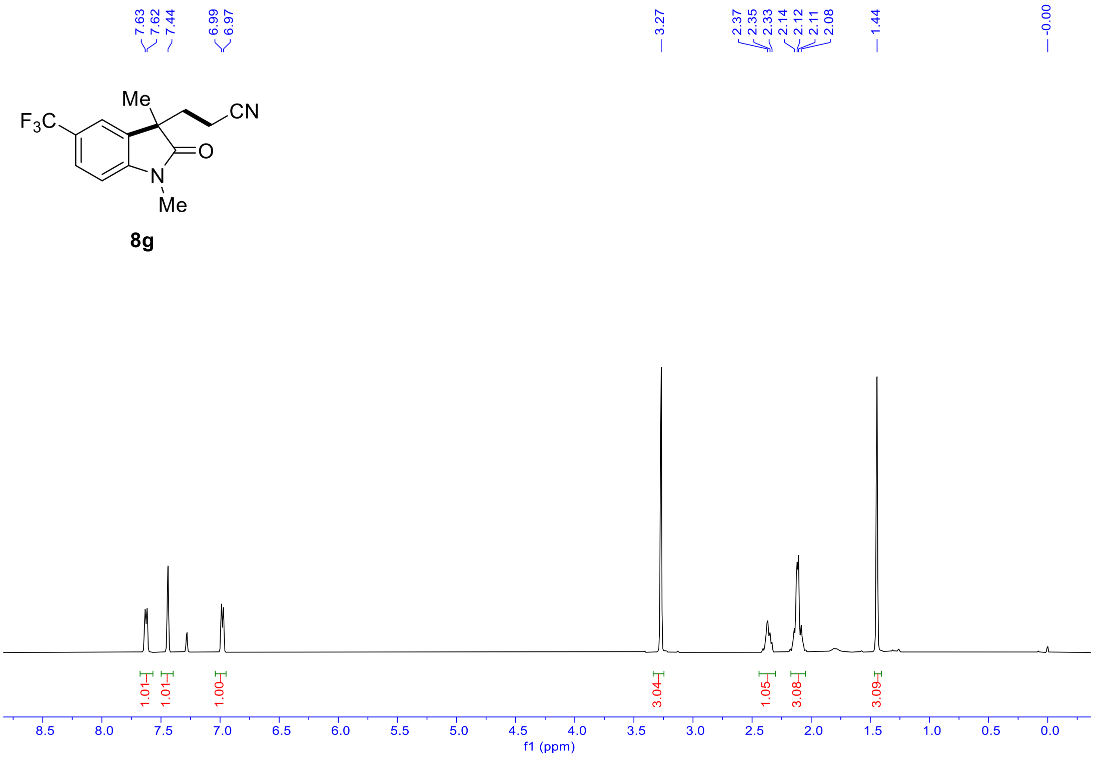

Figure S43: ^{1}H NMR of **8g**, ($CDCl_3$, 500 MHz)

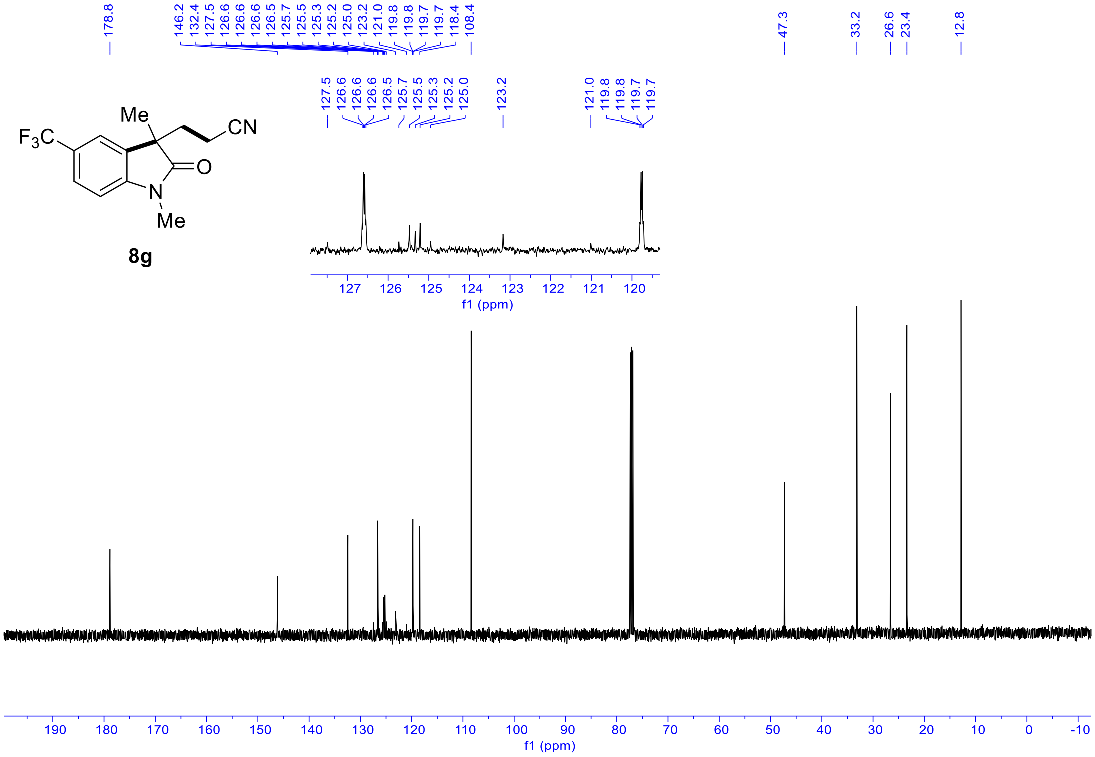

Figure S44: ^{13}C NMR of **8g**, ($CDCl_3$, 126 MHz)

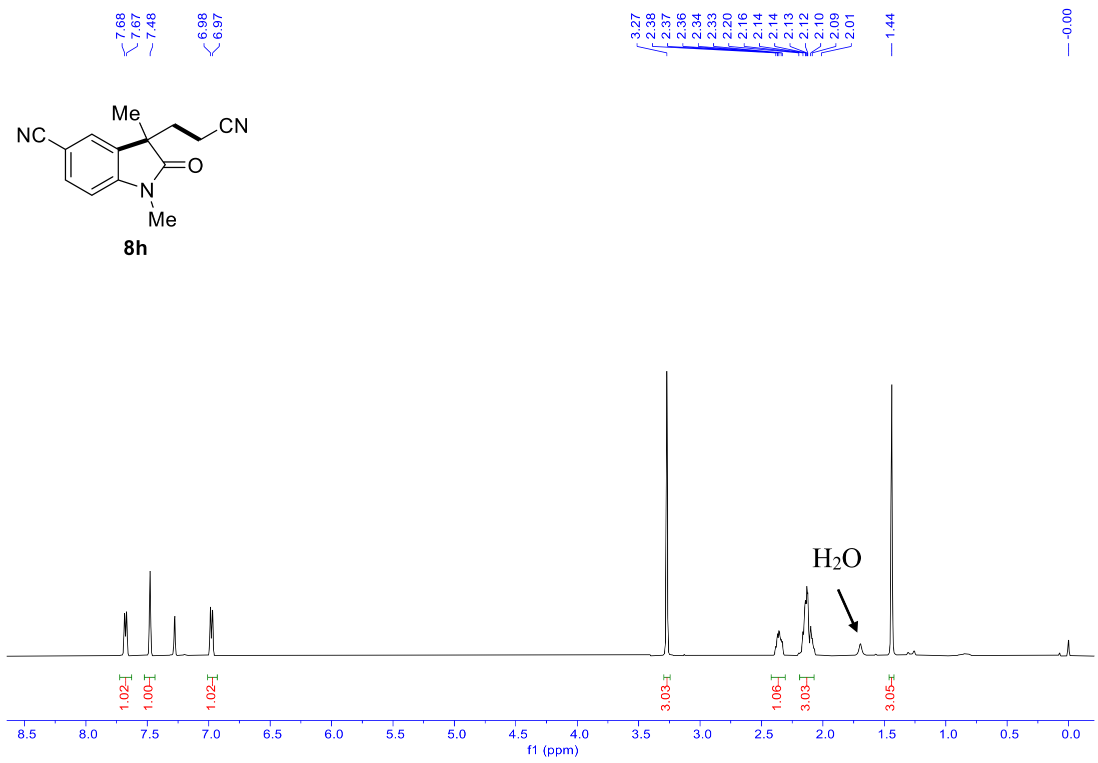

Figure S45: ^{1}H NMR of **8h**, ($CDCl_3$, 500 MHz)

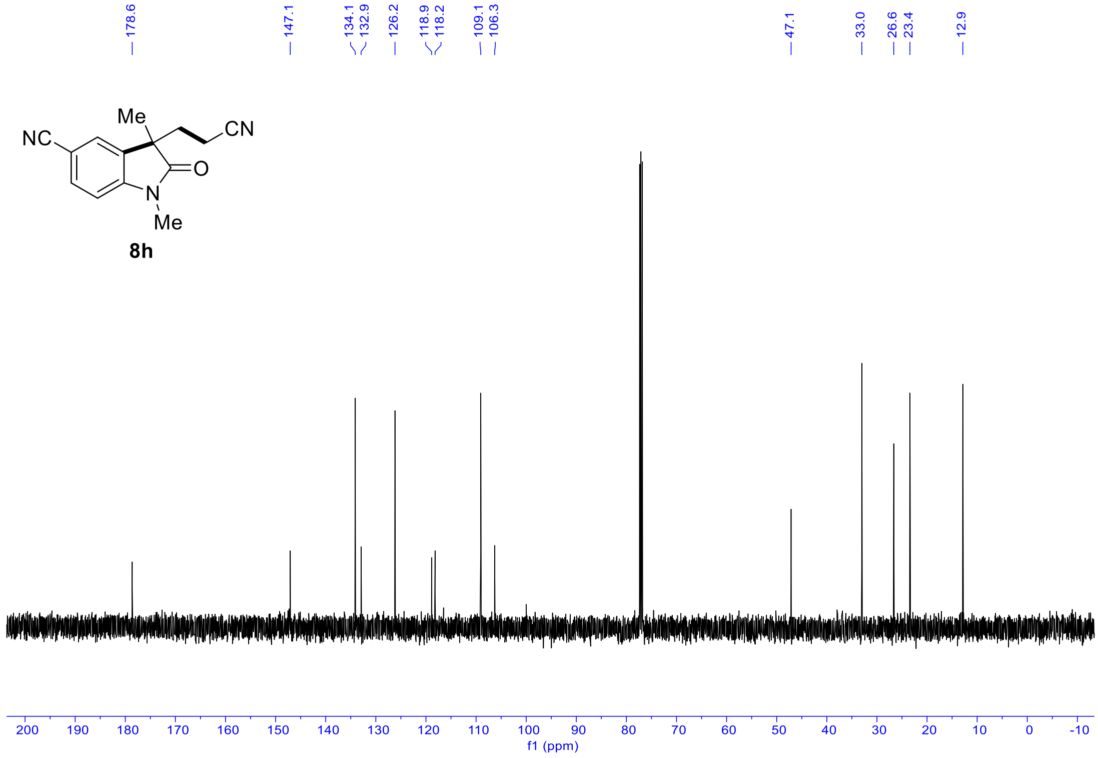

Figure S46: ^{13}C NMR of **8h**, ($CDCl_3$, 126 MHz)

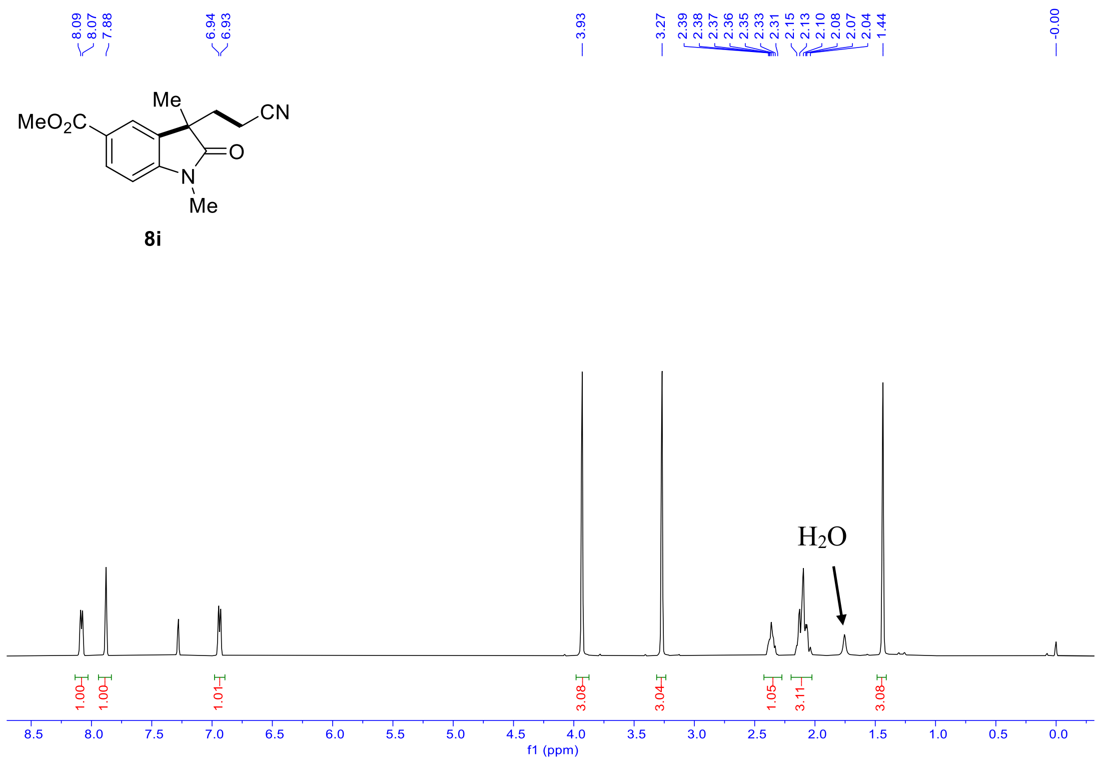

Figure S47: ^{1}H NMR of **8i**, ($CDCl_3$, 500 MHz)

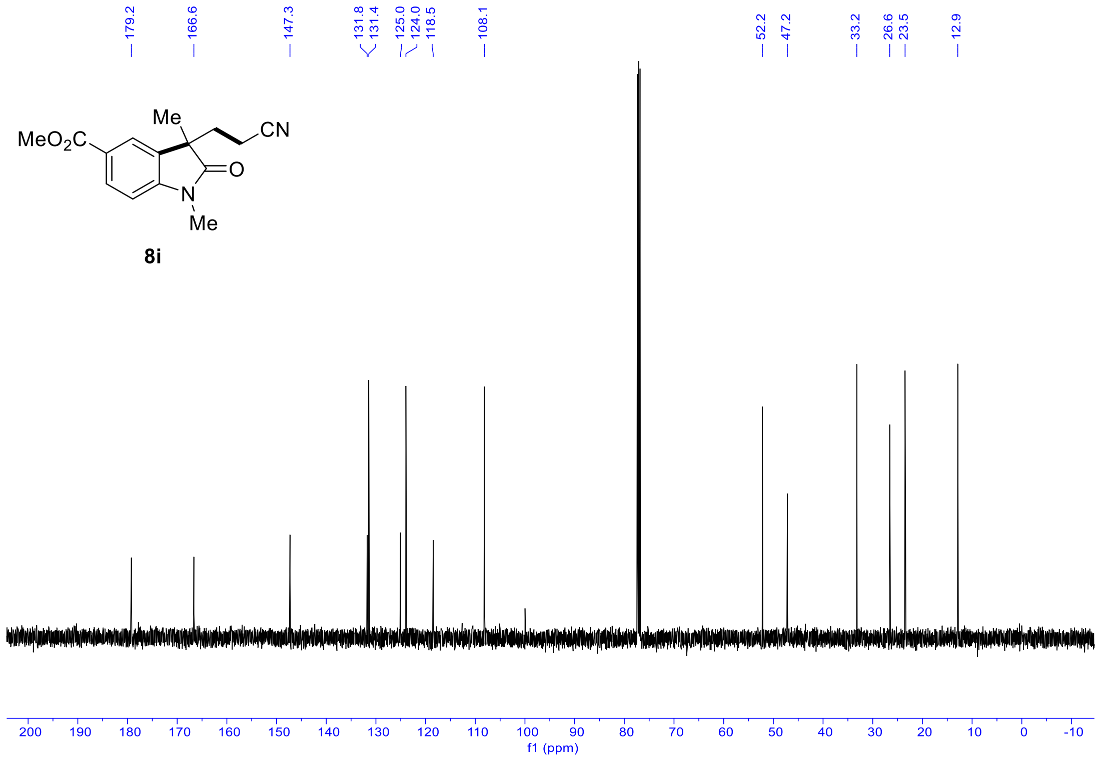

Figure S48: ^{13}C NMR of **8i**, ($CDCl_3$, 126 MHz)

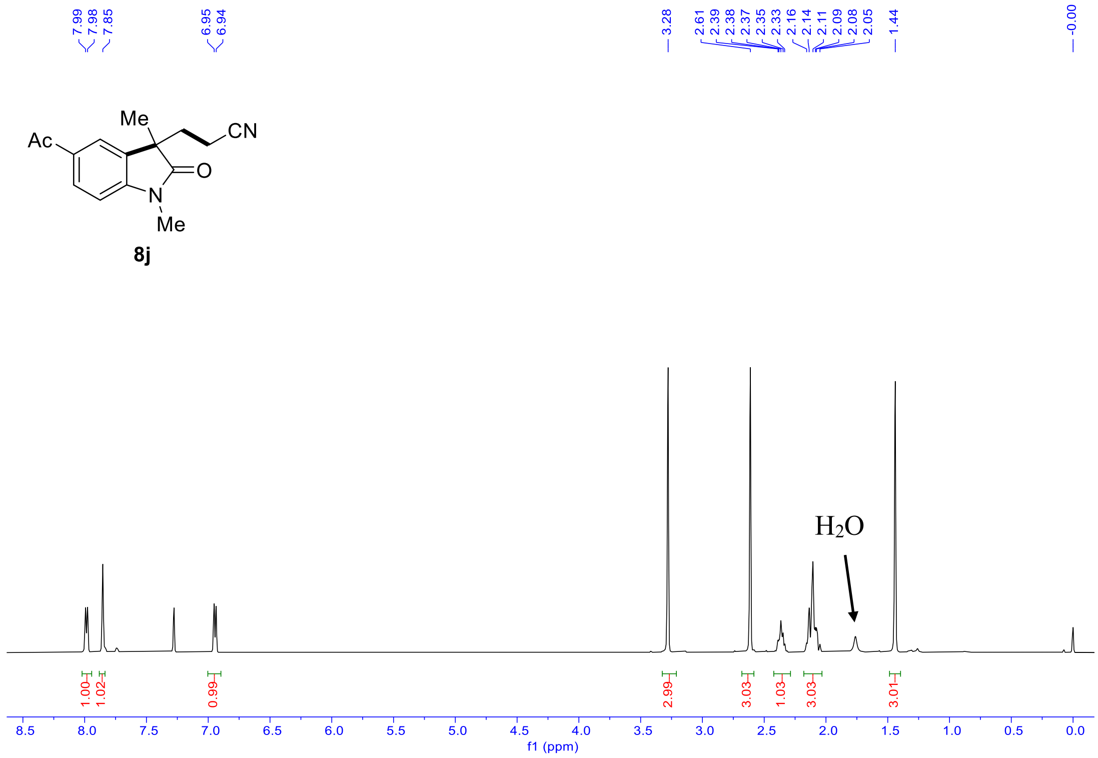

Figure S49: ^{1}H NMR of **8j**, ($CDCl_3$, 500 MHz)

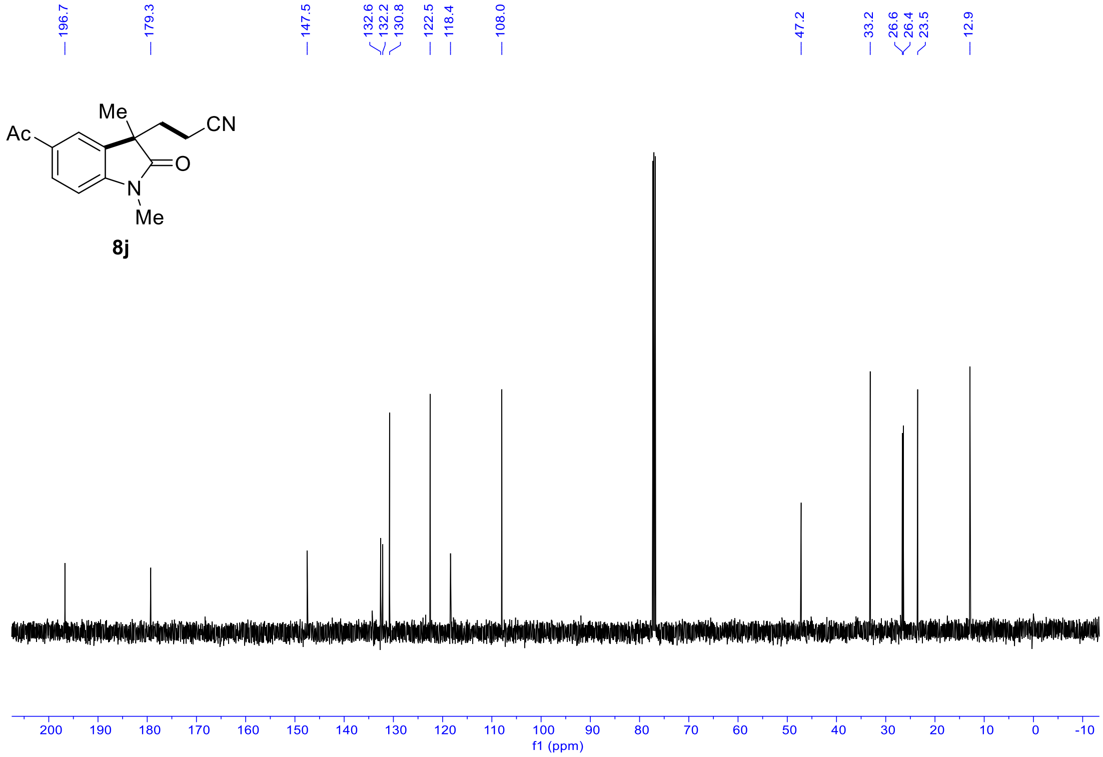

Figure S50: ^{13}C NMR of **8j**, ($CDCl_3$, 126 MHz)

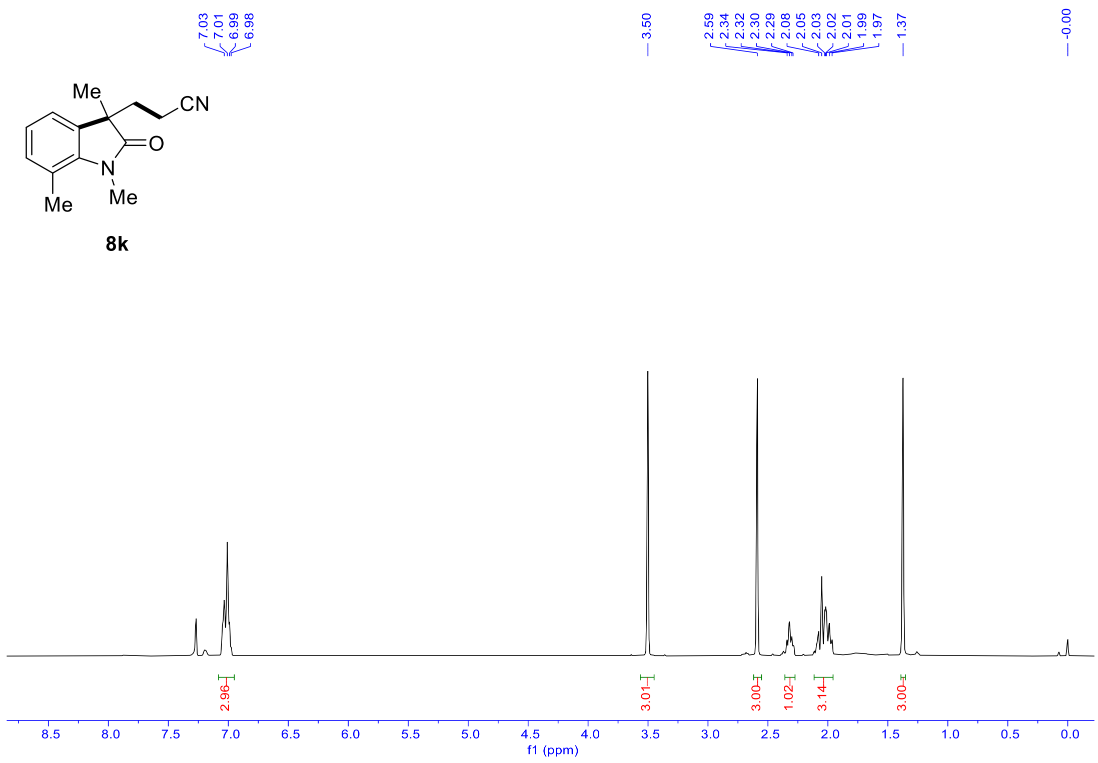

Figure S51: ^{1}H NMR of **8k**, ($CDCl_3$, 500 MHz)

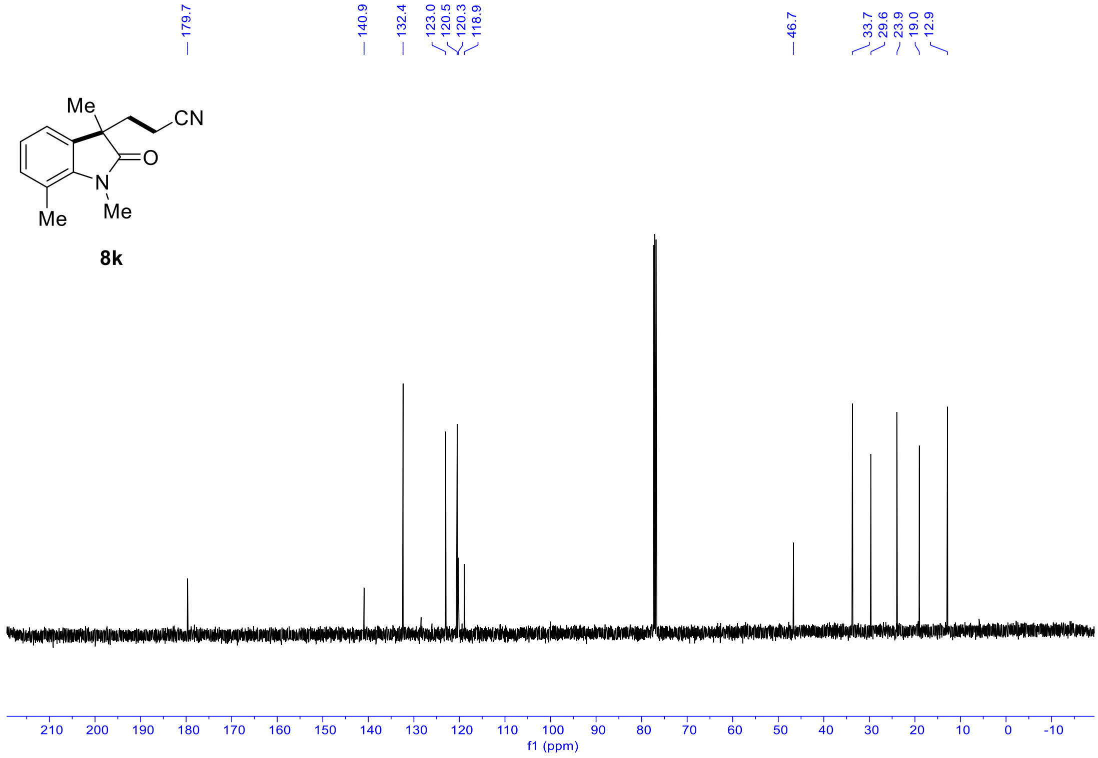

Figure S52: ^{13}C NMR of **8k**, ($CDCl_3$, 126 MHz)

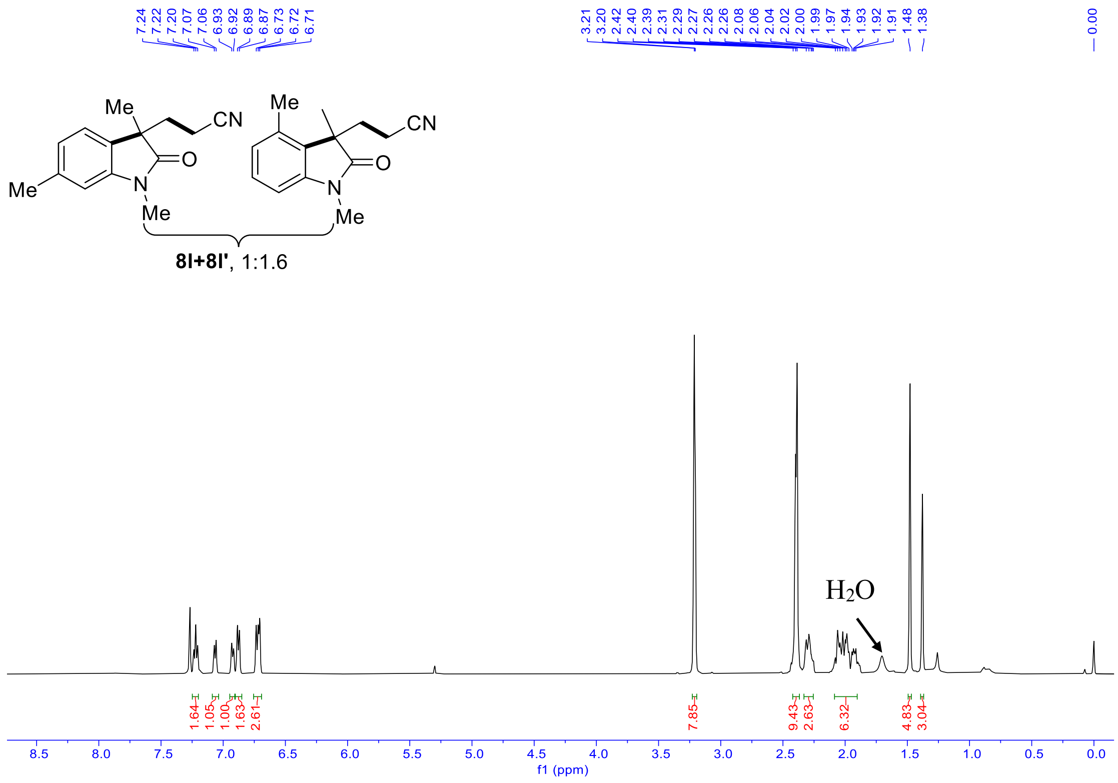

Figure S53: ^{1}H NMR of **8l+8l'**, ($CDCl_3$, 500 MHz)

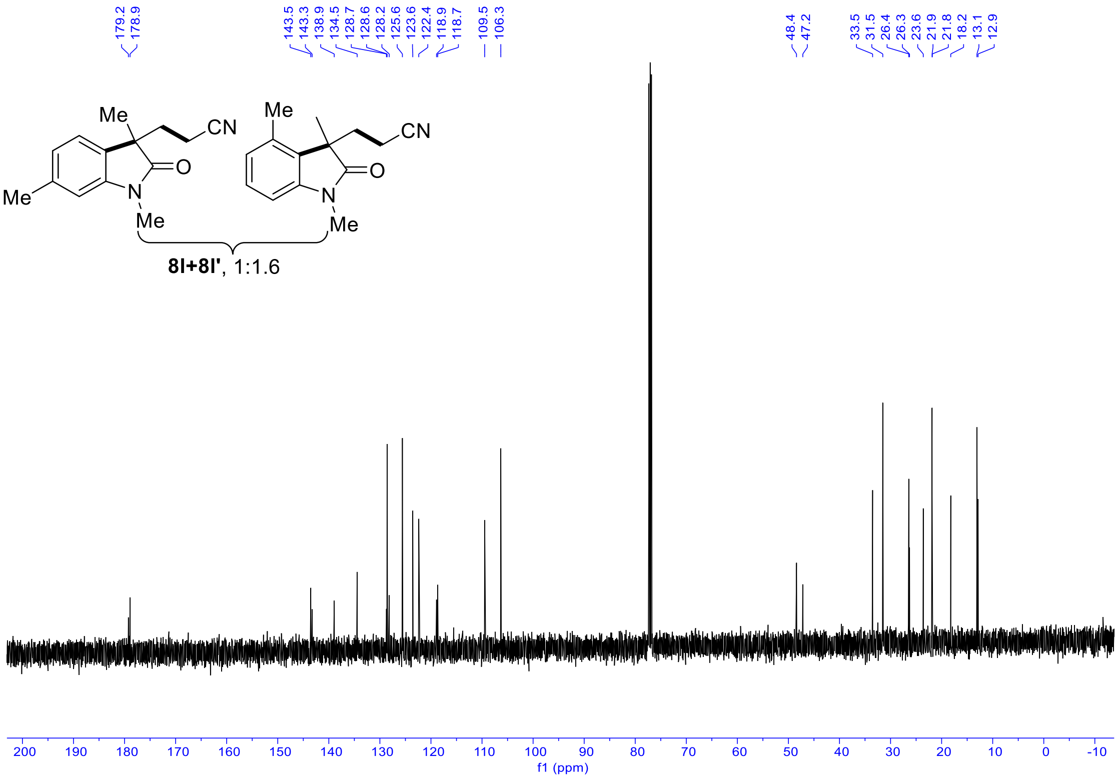

Figure S54: ^{13}C NMR of **8l+8l'**, ($CDCl_3$, 126 MHz)

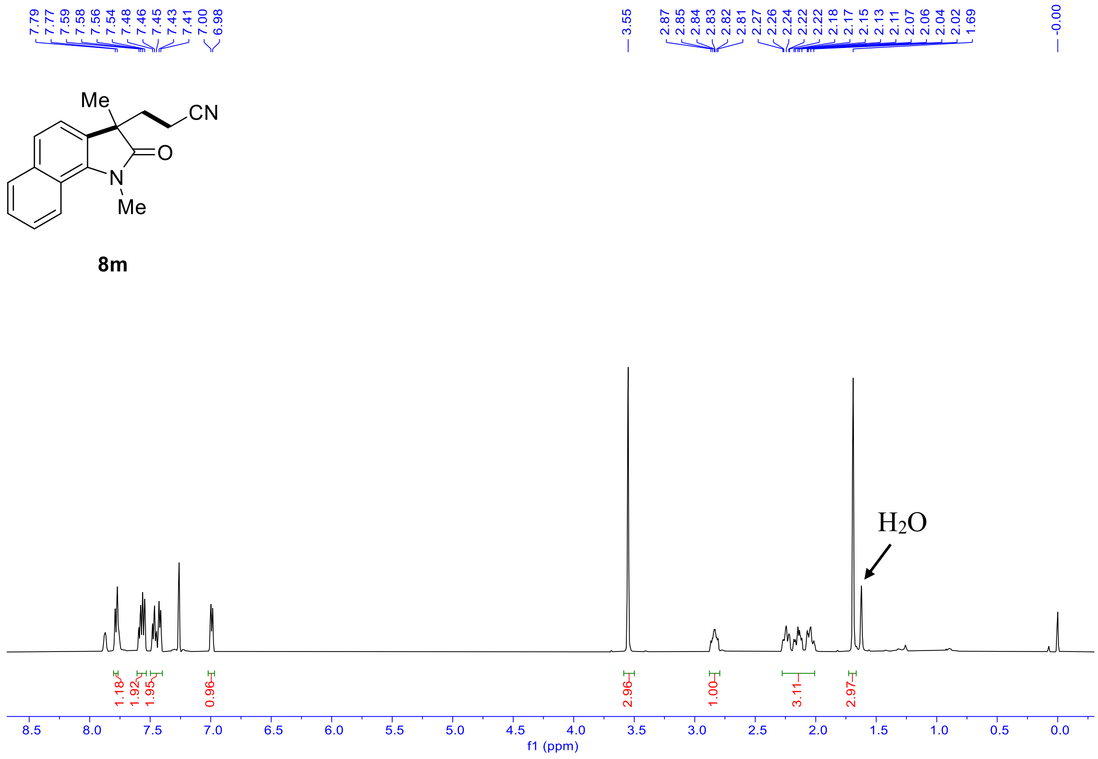

Figure S55: ^{1}H NMR of **8m**, ($CDCl_3$, 500 MHz)

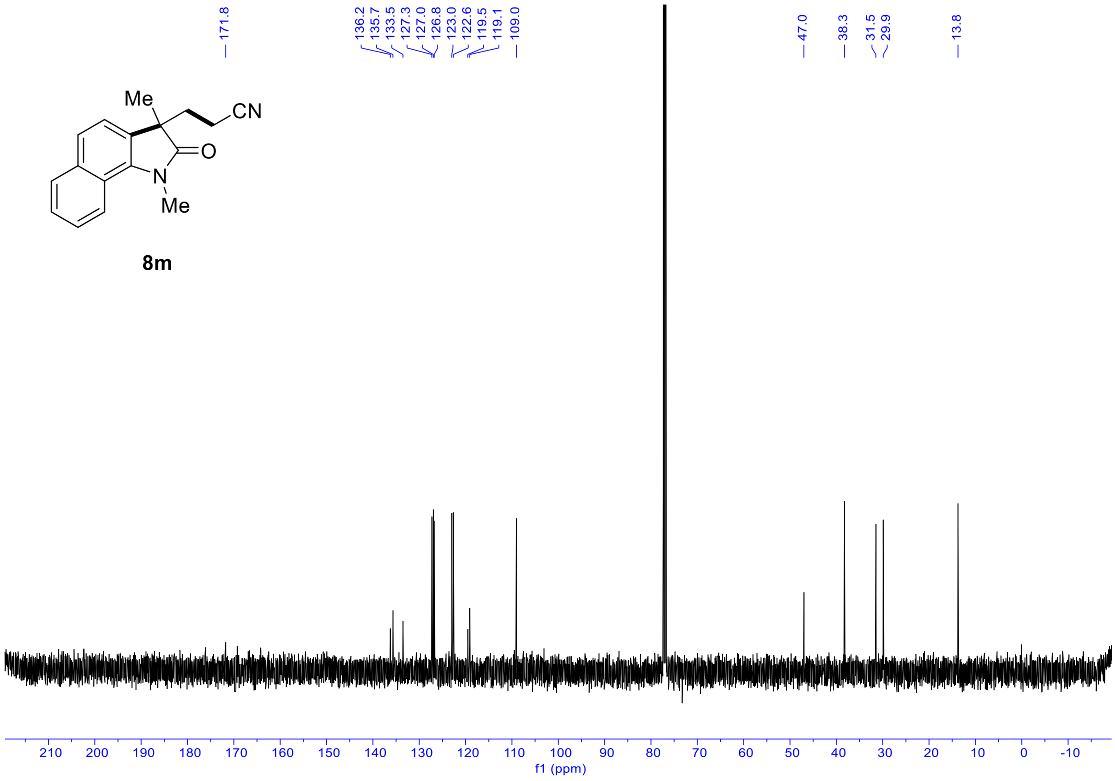

Figure S56: ^{13}C NMR of **8m**, ($CDCl_3$, 126 MHz)

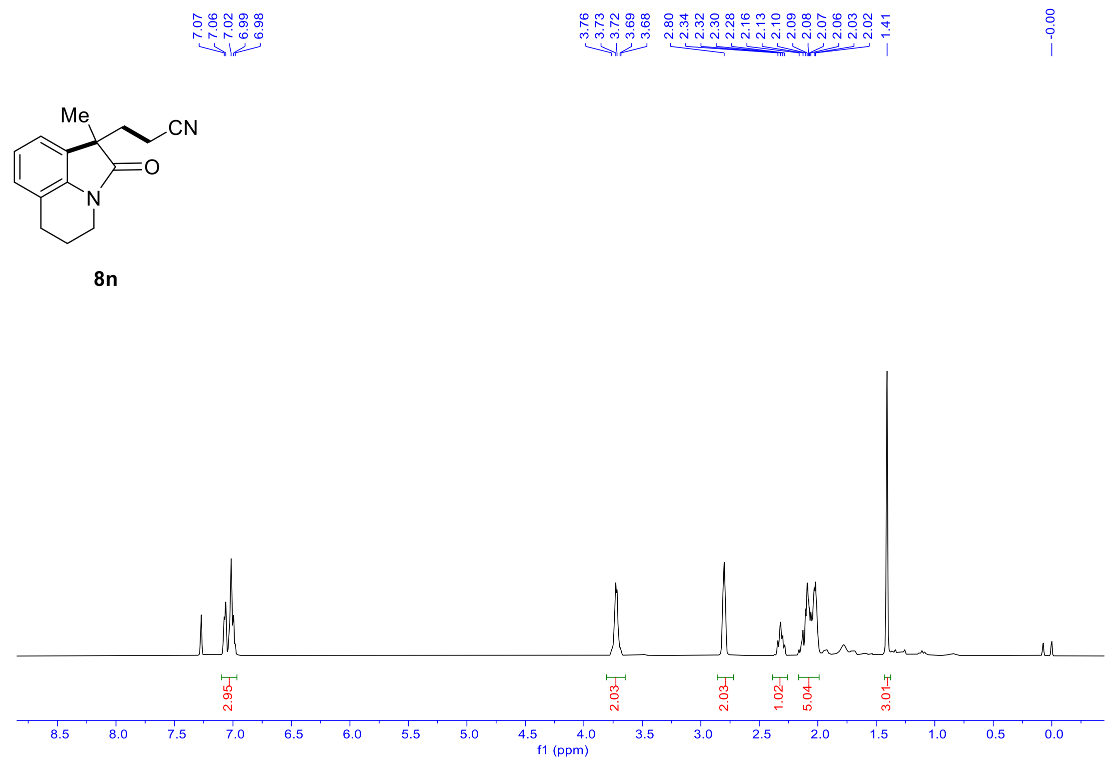

Figure S57: ^{1}H NMR of **8n**, ($CDCl_3$, 500 MHz)

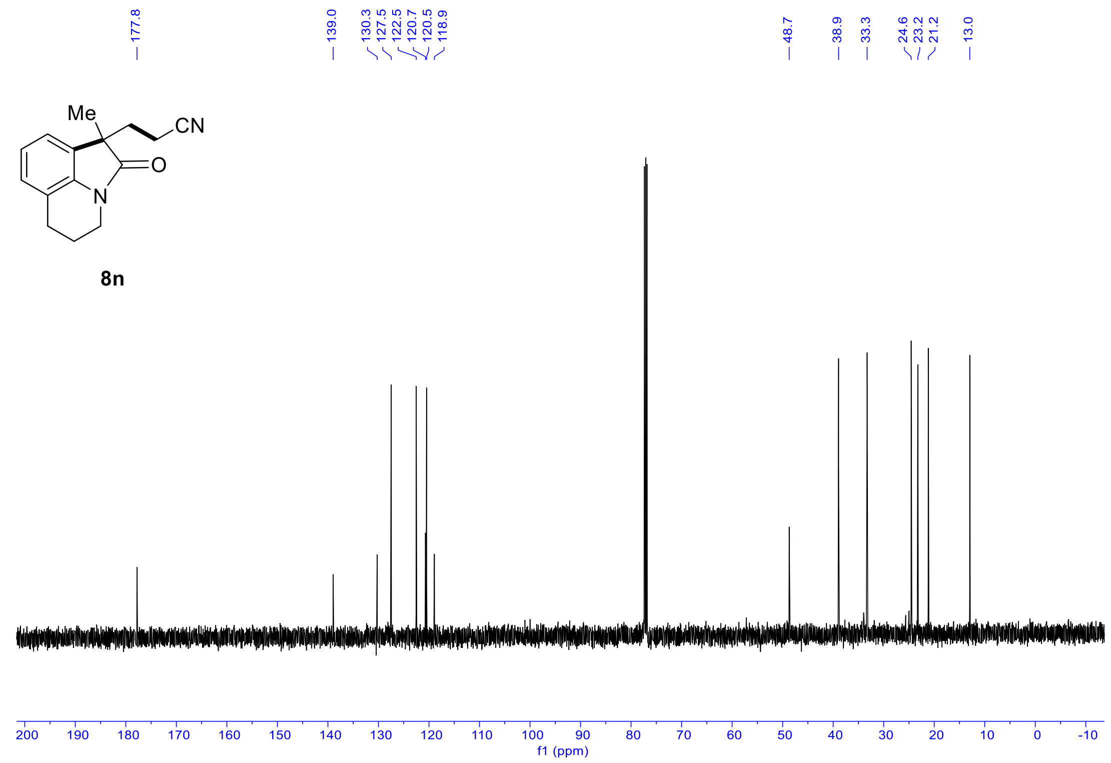

Figure S58: ^{13}C NMR of **8n**, ($CDCl_3$, 126 MHz)

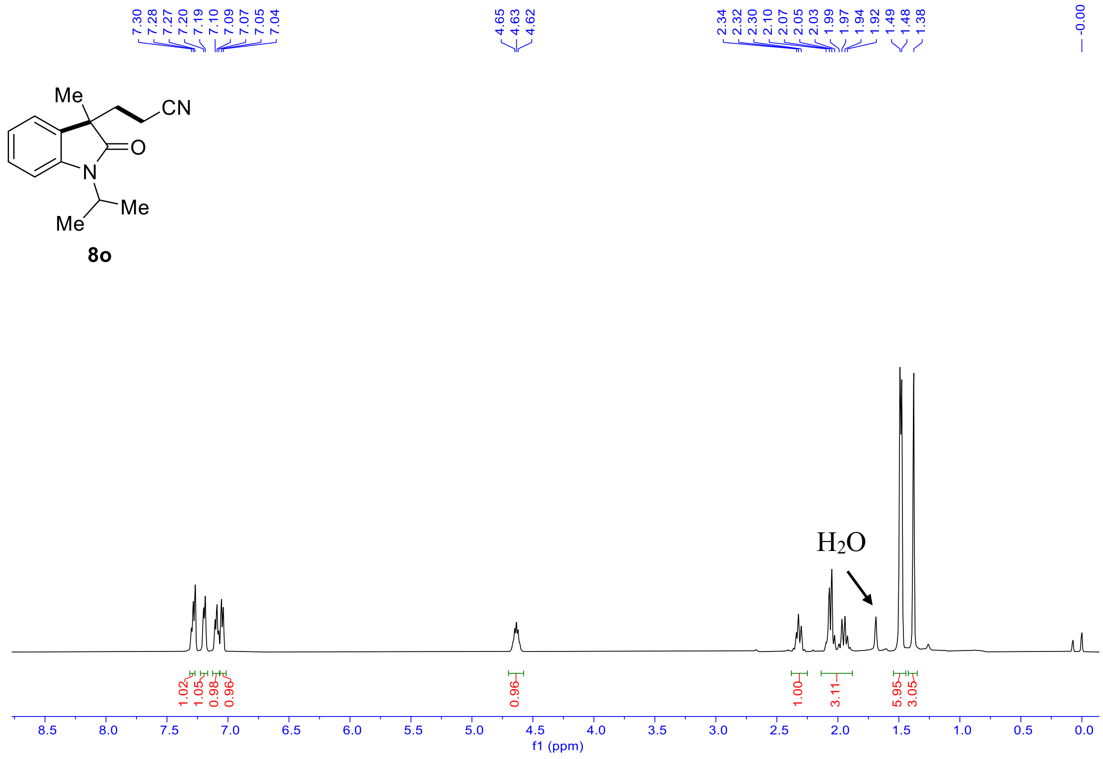

Figure S59: ^{1}H NMR of **8o**, ($CDCl_3$, 500 MHz)

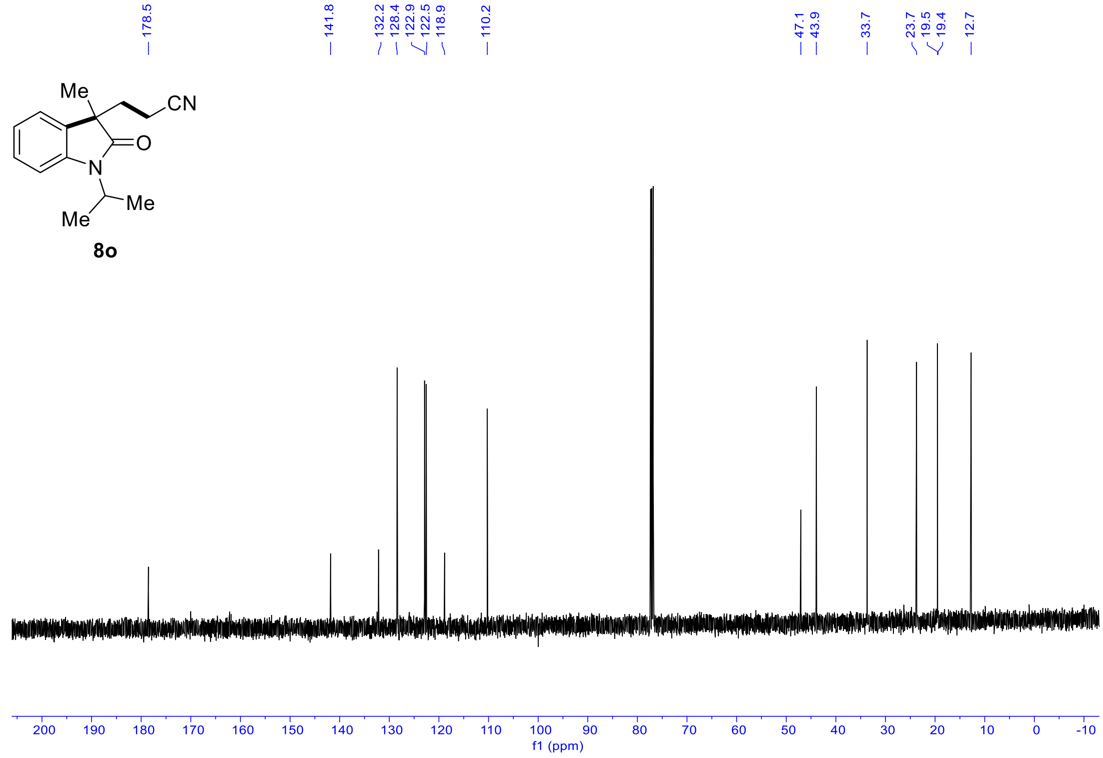

Figure S60: ^{13}C NMR of **8o**, ($CDCl_3$, 126 MHz)

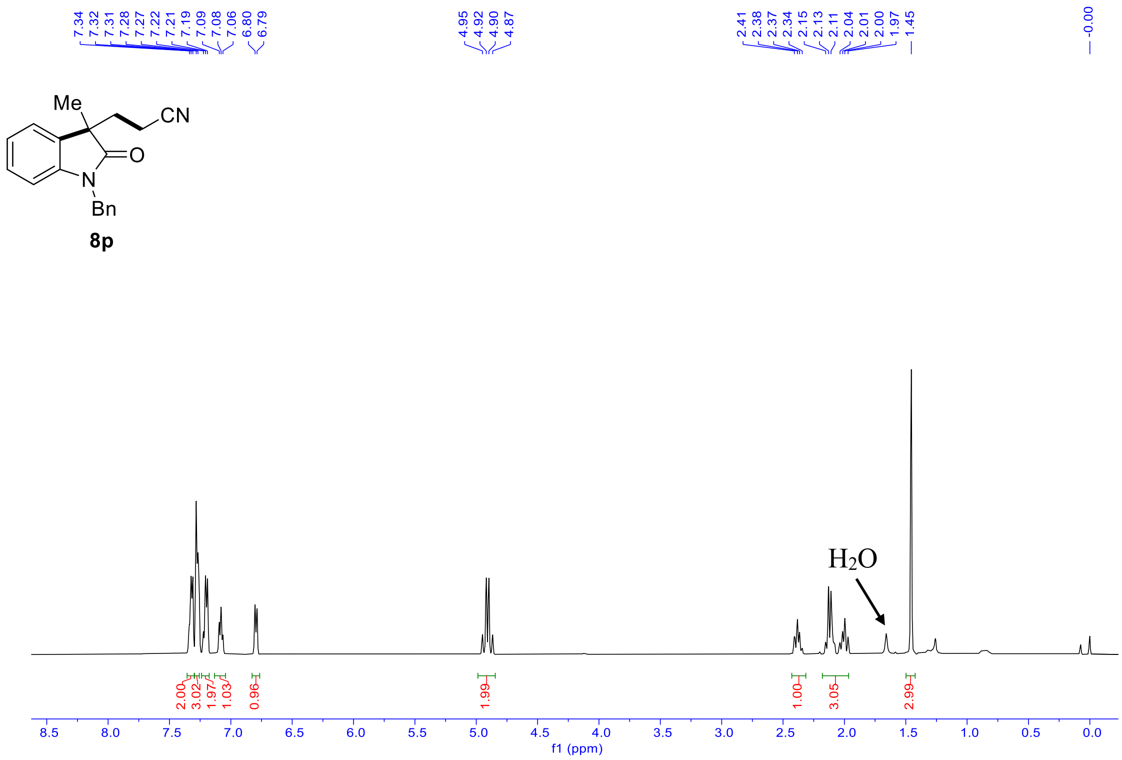

Figure S61: ^{1}H NMR of **8p**, ($CDCl_3$, 500 MHz)

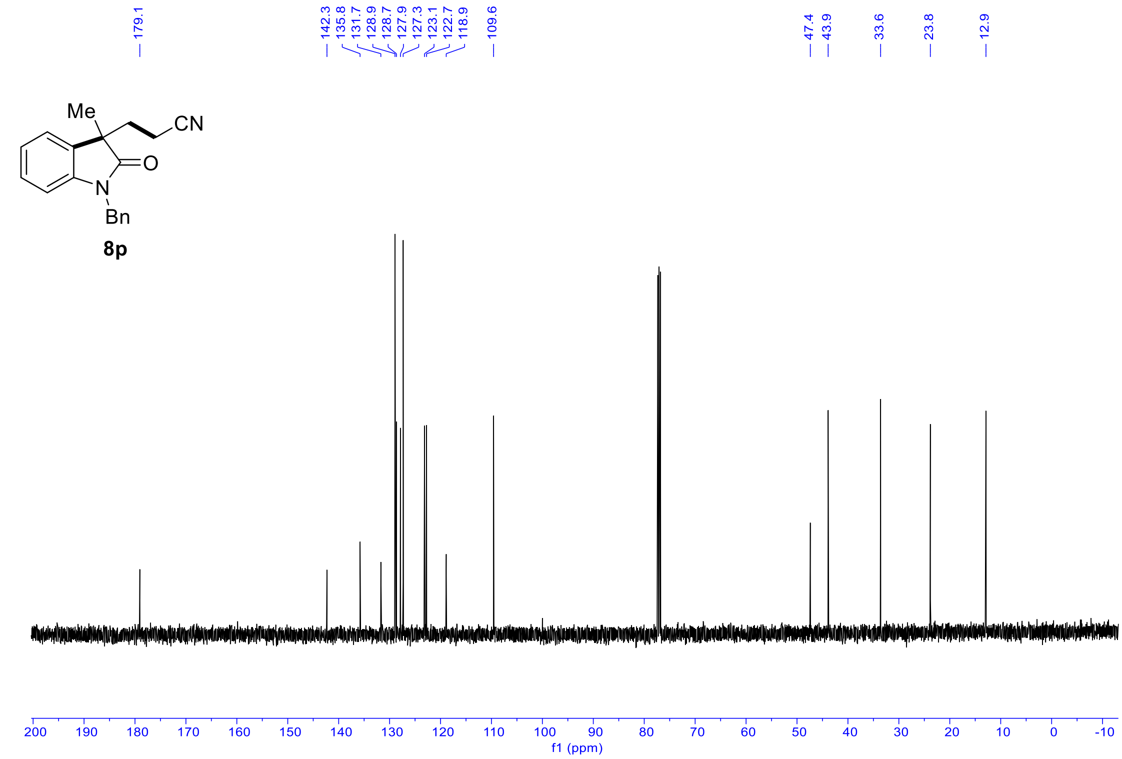

Figure S62: ^{13}C NMR of **8p**, ($CDCl_3$, 126 MHz)

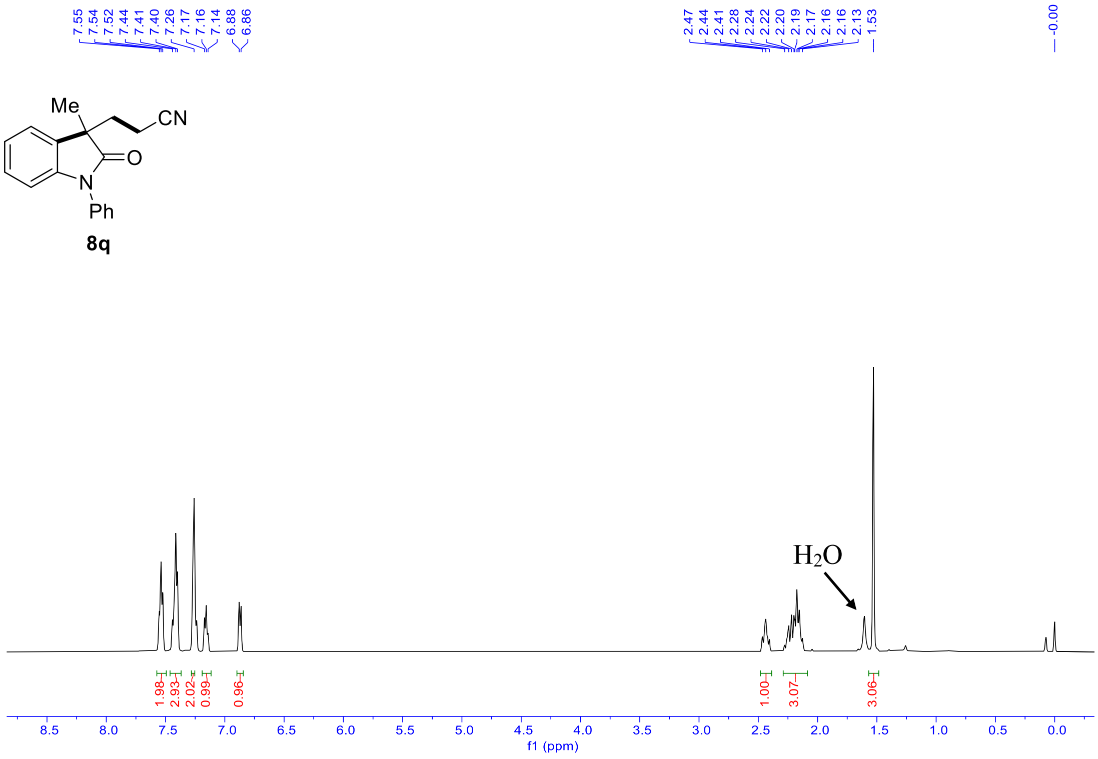

Figure S63: ^{1}H NMR of **8q**, ($CDCl_3$, 500 MHz)

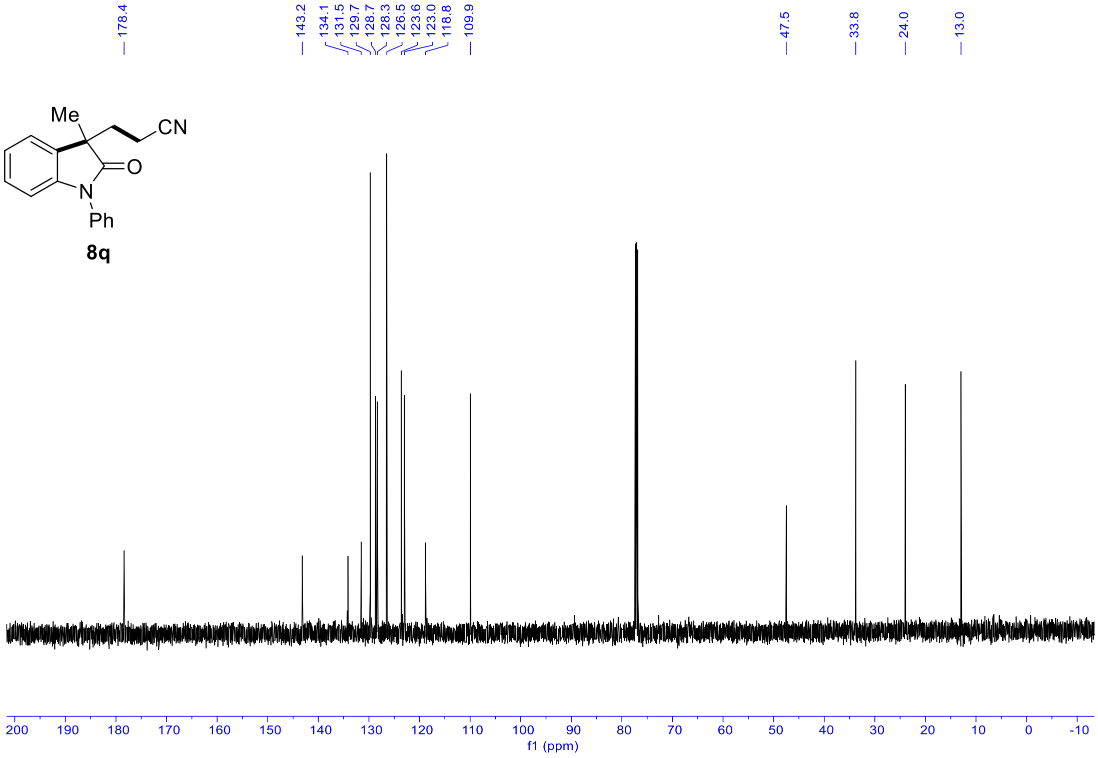

Figure S64: ^{13}C NMR of **8q**, ($CDCl_3$, 126 MHz)

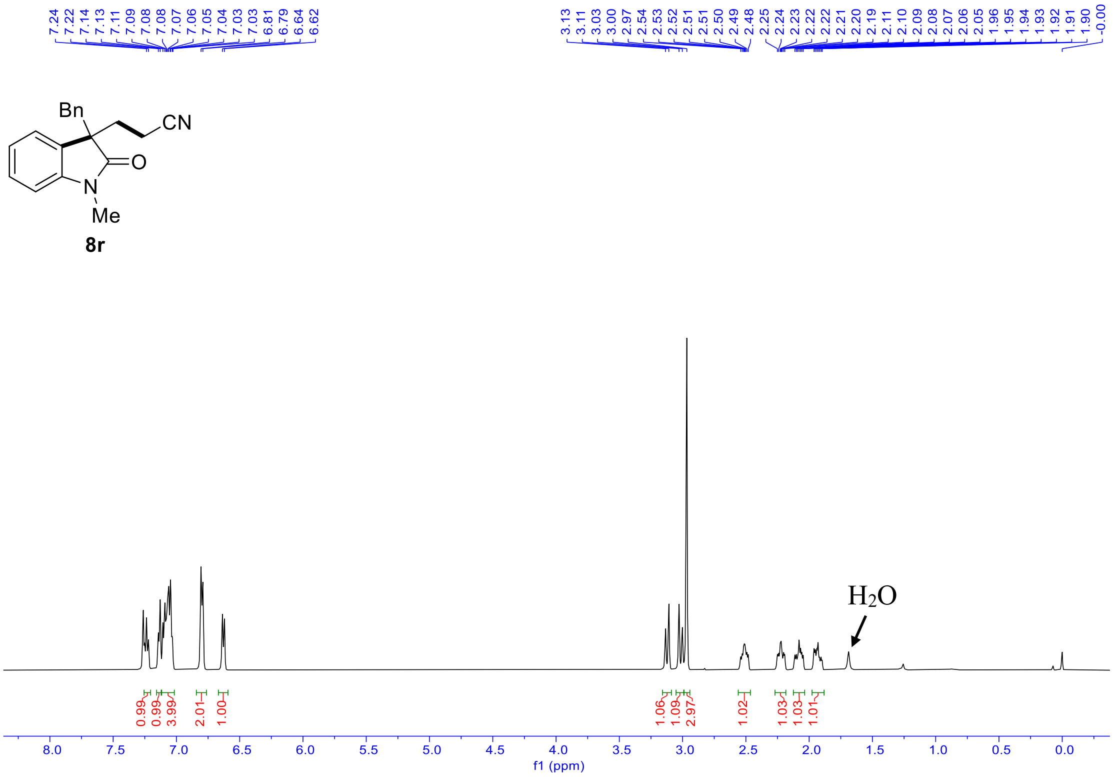

Figure S65: ^{1}H NMR of **8r**, ($CDCl_3$, 500 MHz)

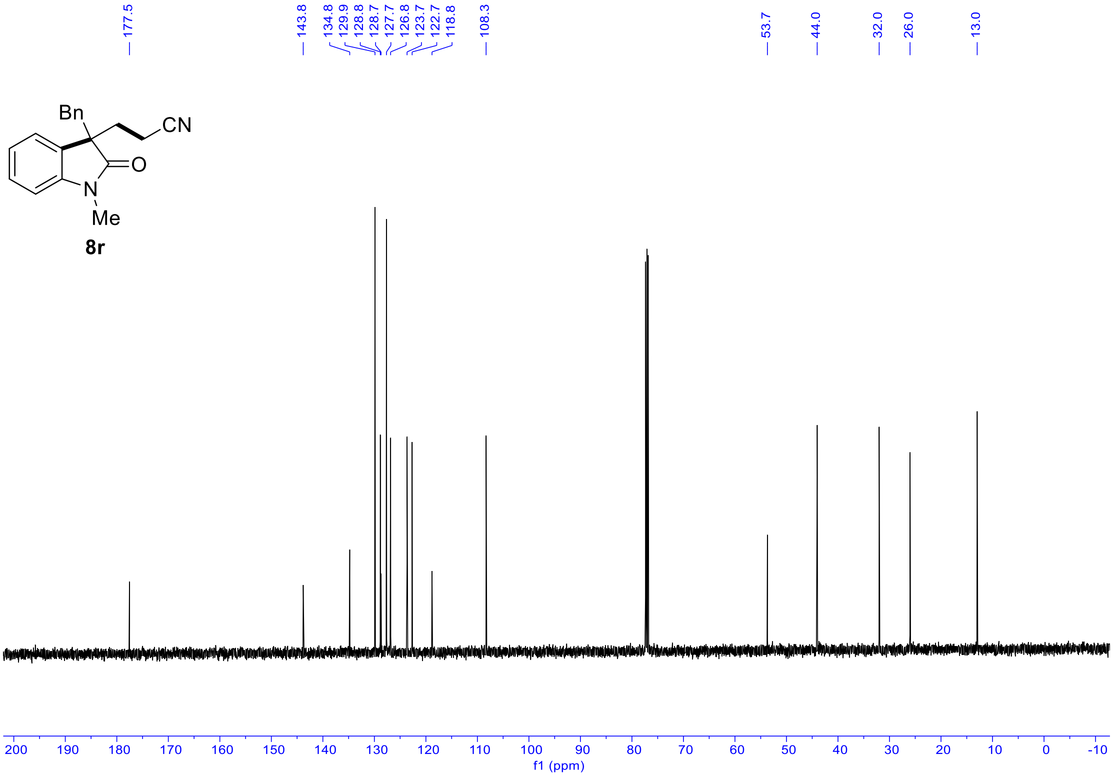

Figure S66: ^{13}C NMR of **8r**, ($CDCl_3$, 126 MHz)

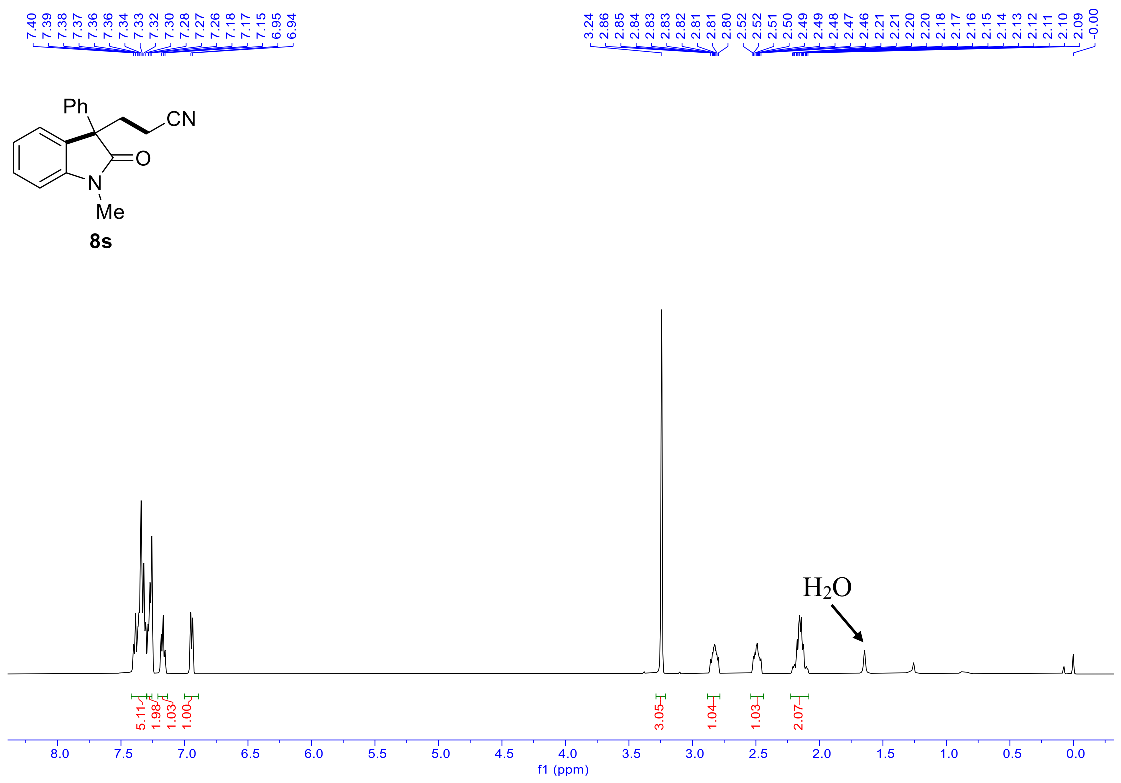

Figure S67: ^{1}H NMR of **8s**, ($CDCl_3$, 500 MHz)

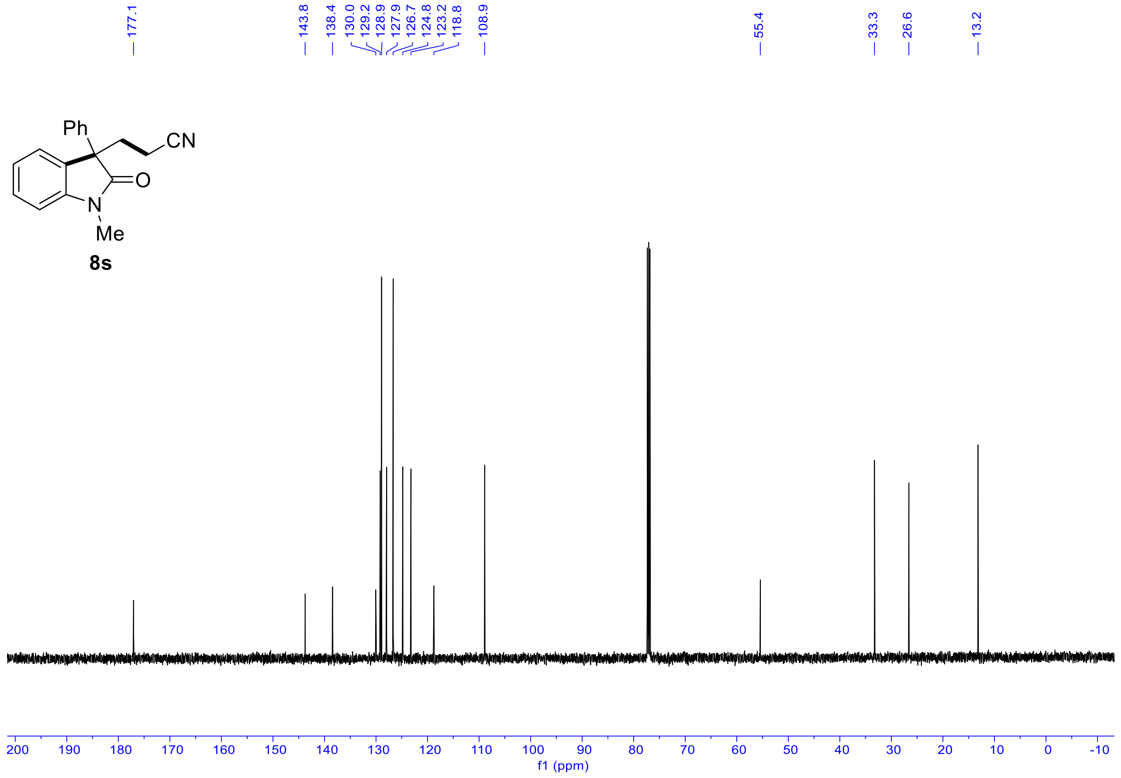

Figure S68: ^{13}C NMR of **8s**, ($CDCl_3$, 126 MHz)

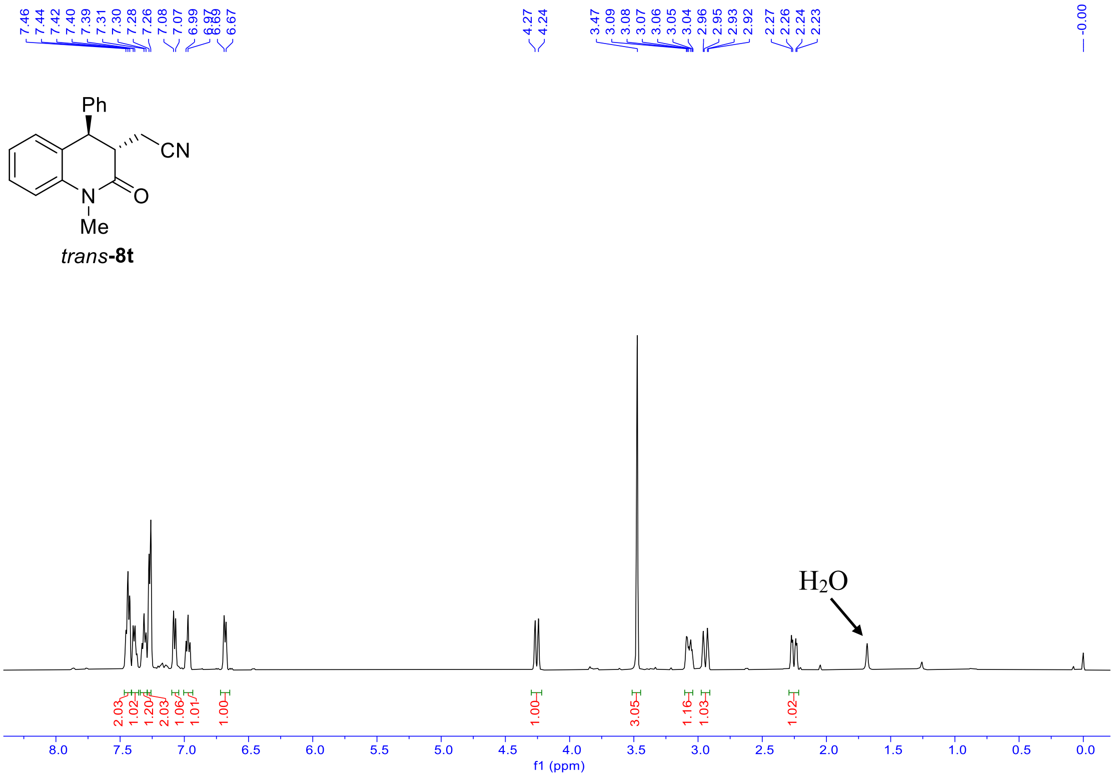

Figure S69: ^{1}H NMR of *trans*-**8t**, ($CDCl_3$, 500 MHz)

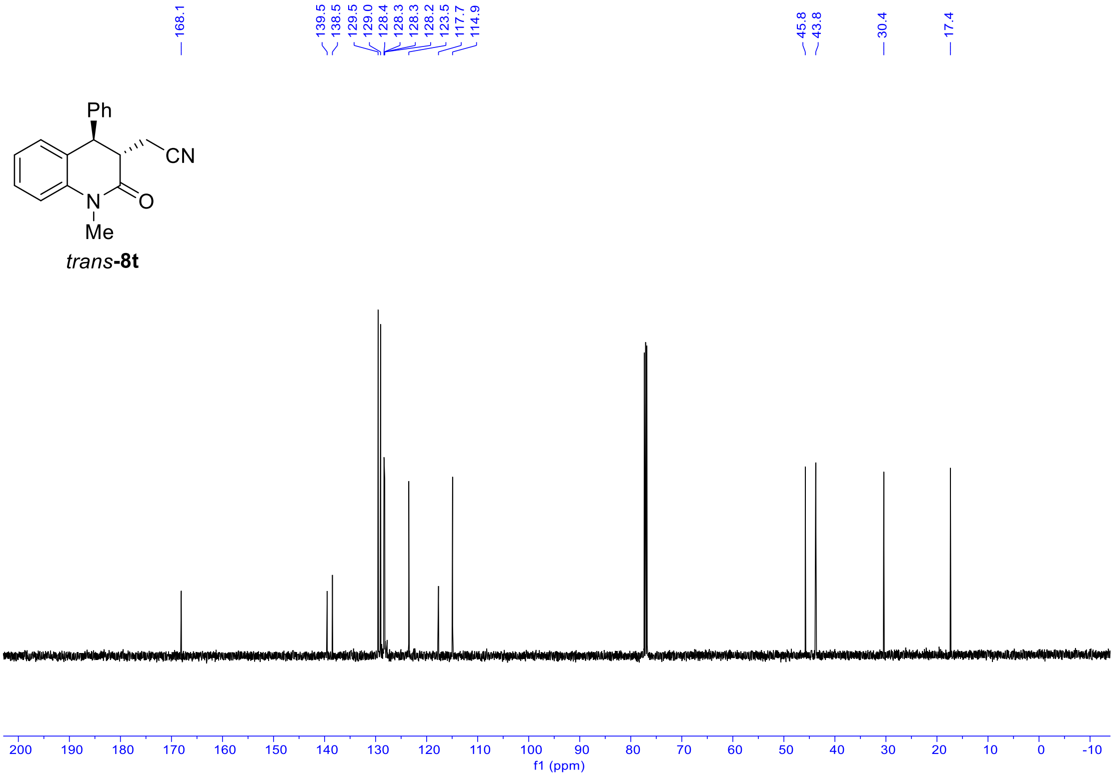

Figure S70: ^{13}C NMR of *trans*-**8t**, ($CDCl_3$, 126 MHz)

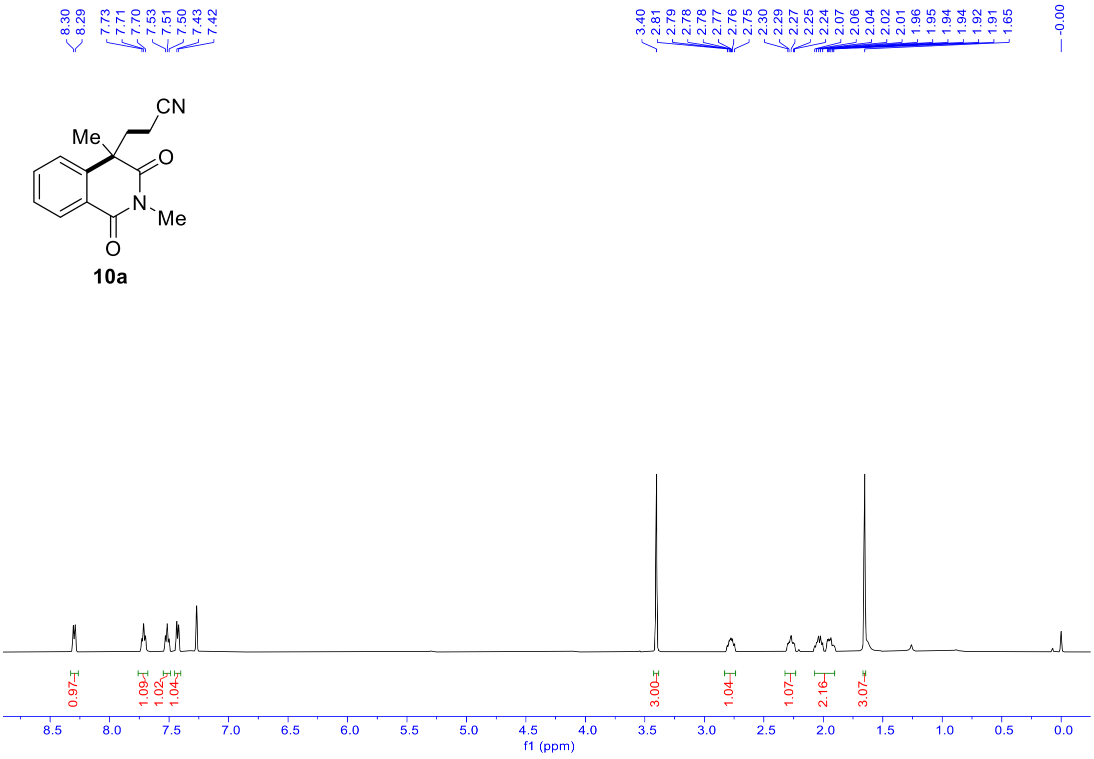

Figure S71: ^{1}H NMR of **10a**, ($CDCl_3$, 500 MHz)

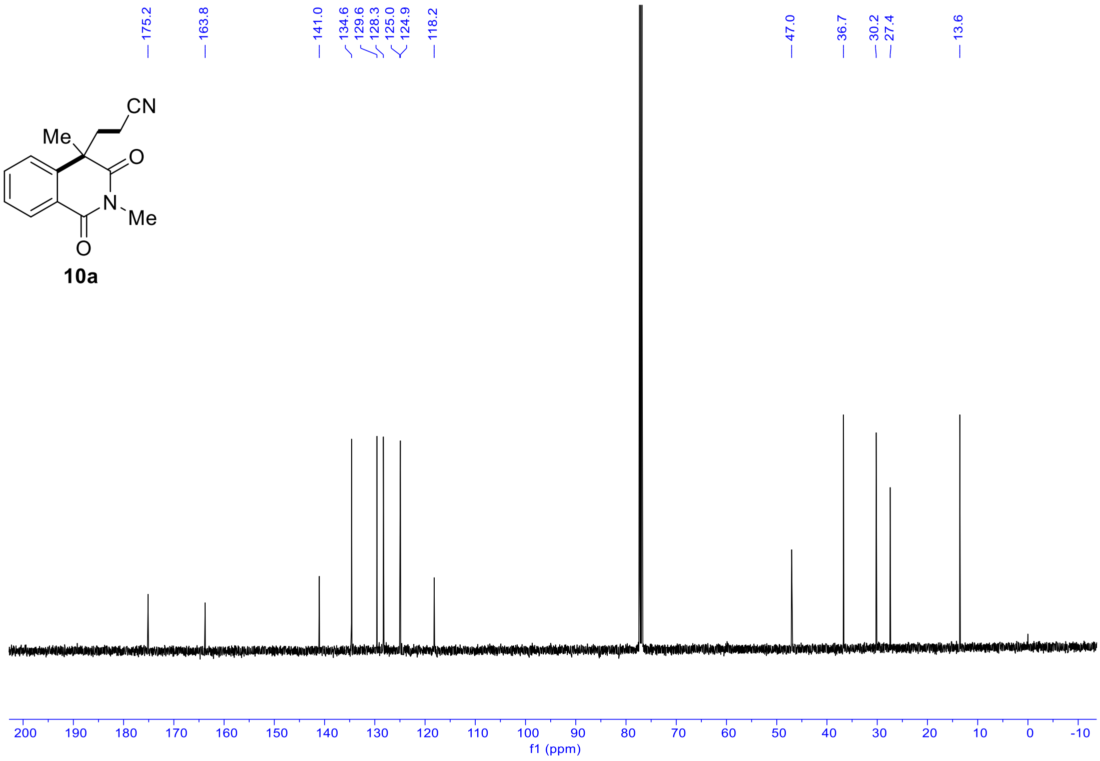

Figure S72: ^{13}C NMR of **10a**, ($CDCl_3$, 126 MHz)

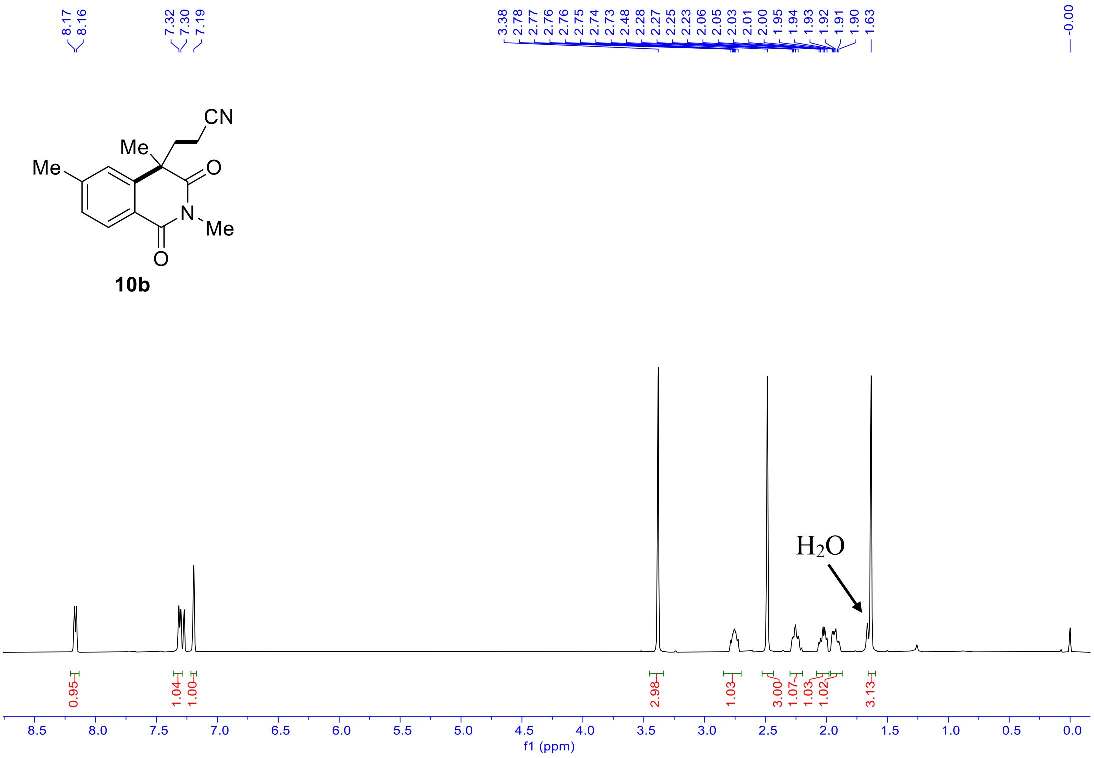

Figure S73: ^{1}H NMR of **10b**, ($CDCl_3$, 500 MHz)

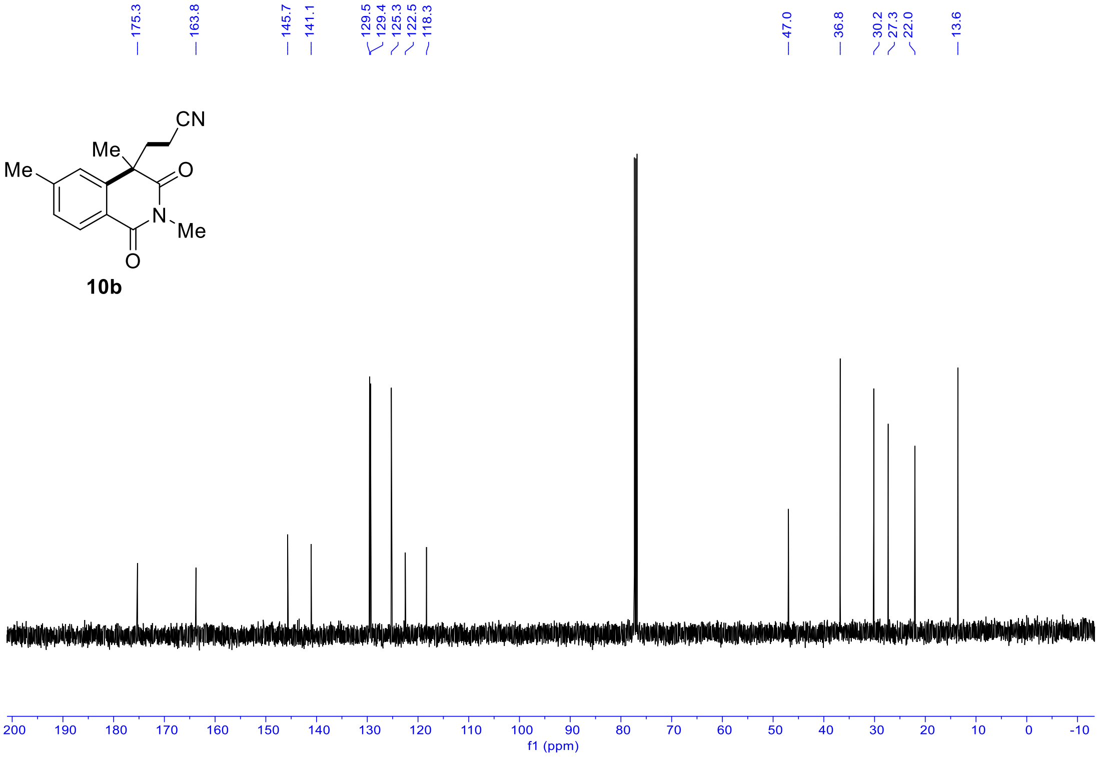

Figure S74: ^{13}C NMR of **10b**, ($CDCl_3$, 126 MHz)

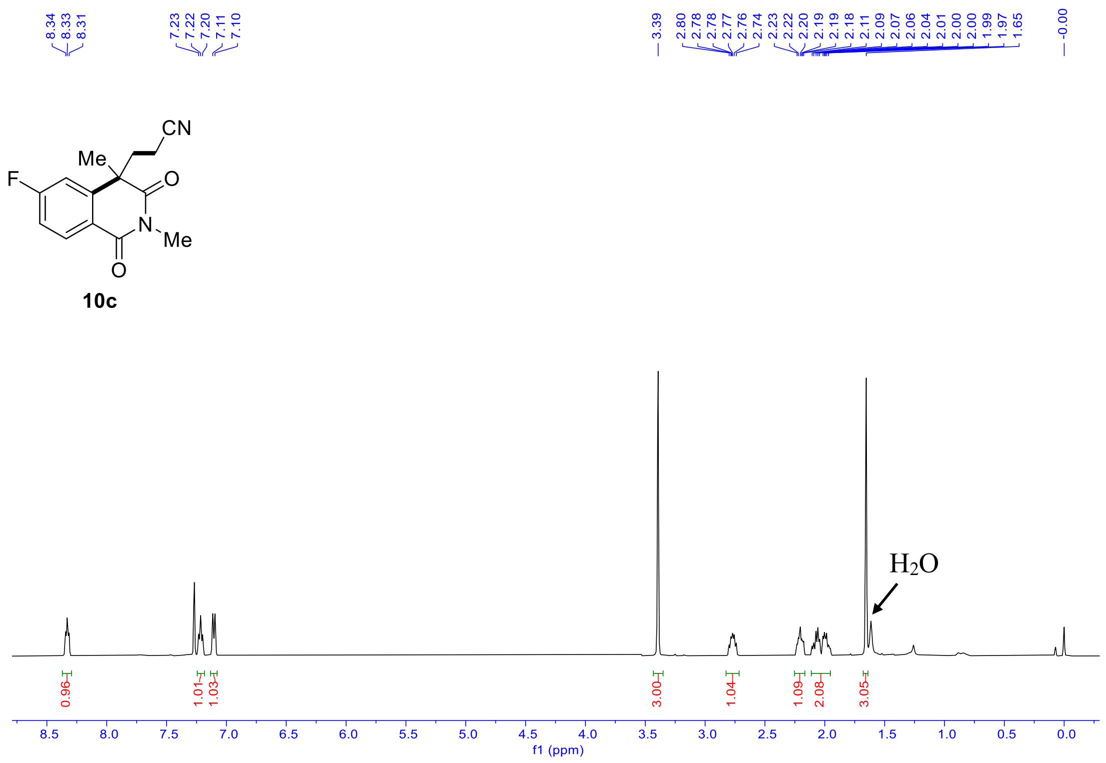

Figure S75: ^{1}H NMR of **10c**, ($CDCl_3$, 500 MHz)

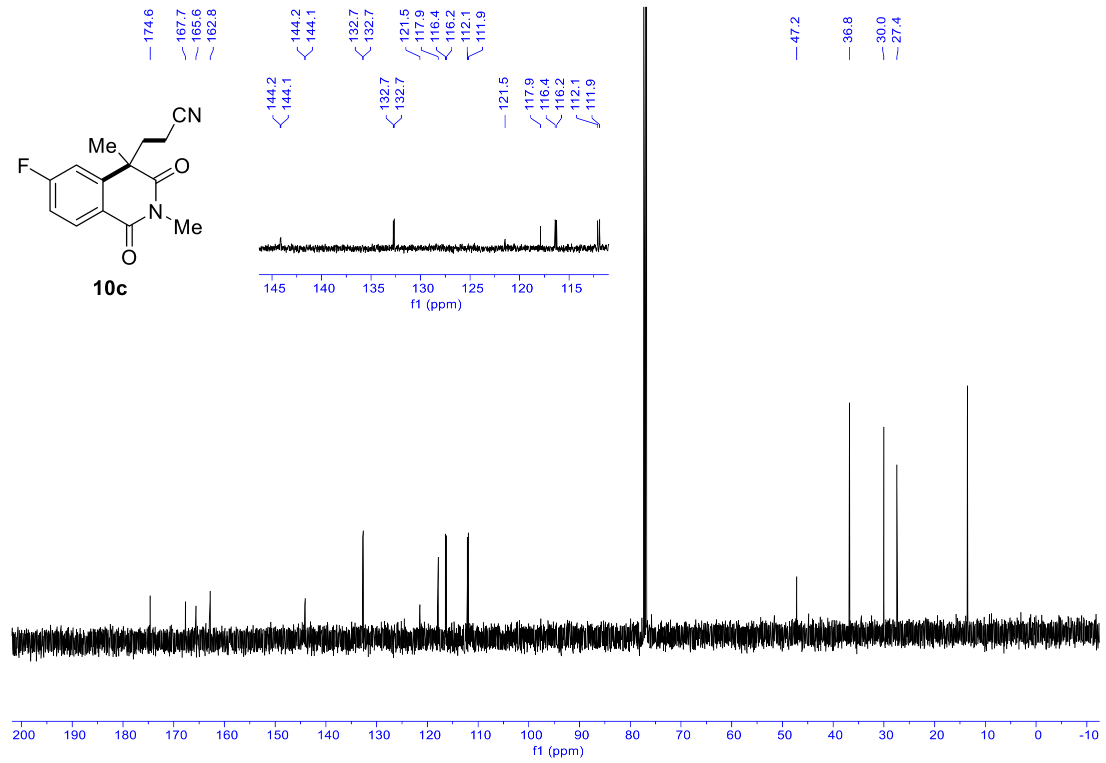

Figure S76: ^{13}C NMR of **10c**, ($CDCl_3$, 126 MHz)

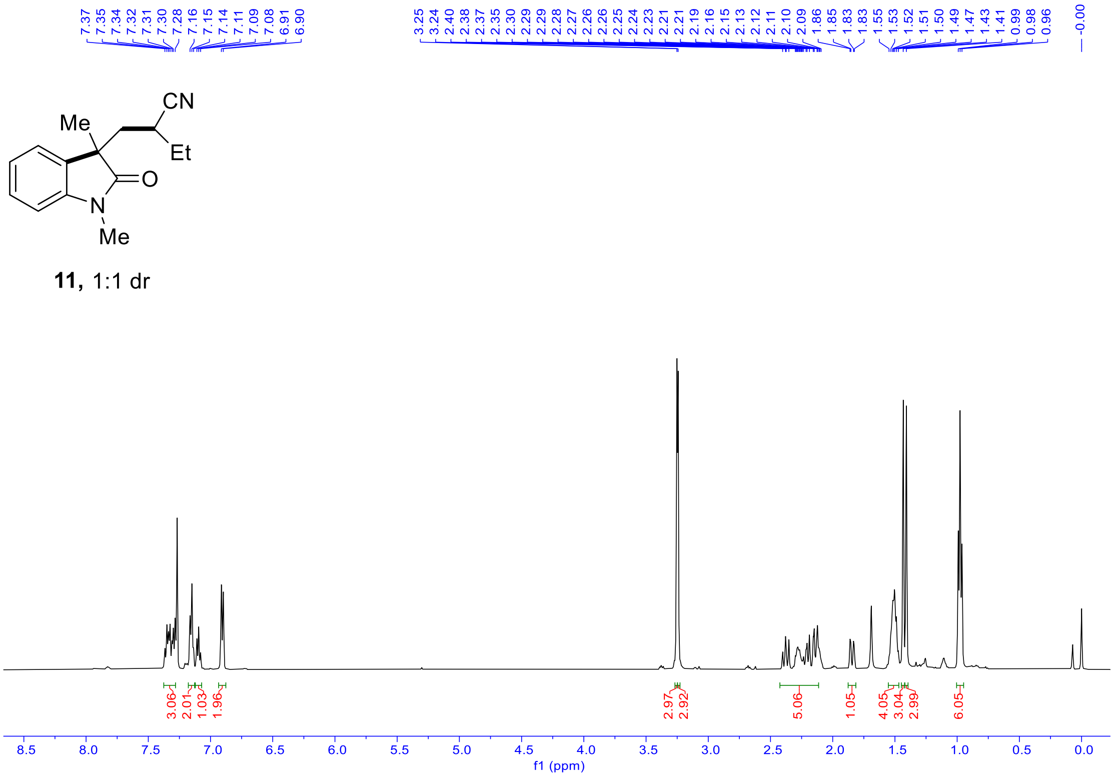

Figure S77: ^{1}H NMR of **11**, ($CDCl_3$, 500 MHz)

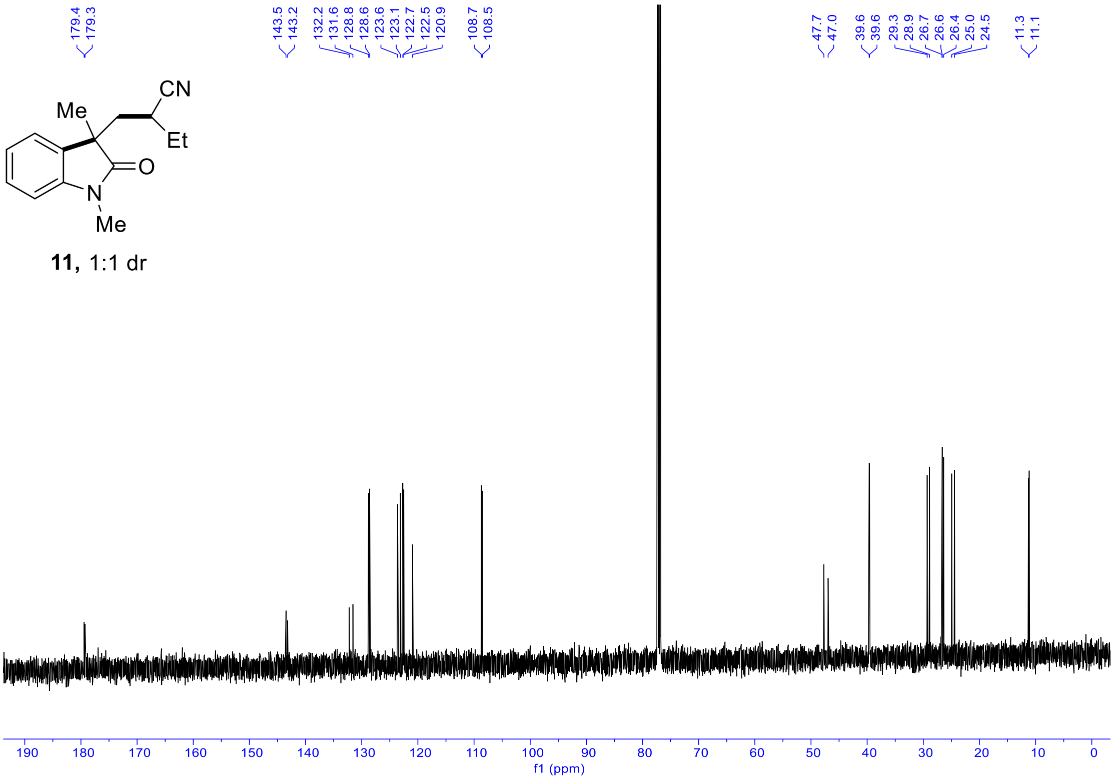

Figure S78: ^{13}C NMR of **11**, ($CDCl_3$, 126 MHz)

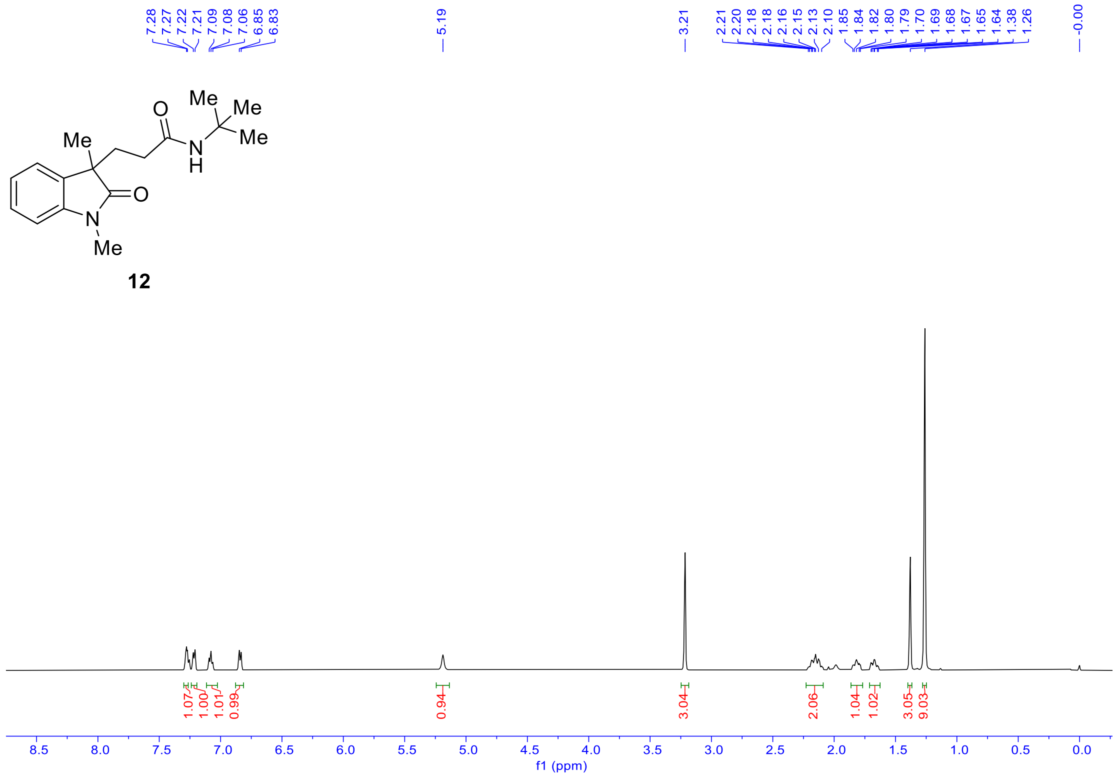

Figure S79: ^{1}H NMR of **12**, ($CDCl_3$, 500 MHz)

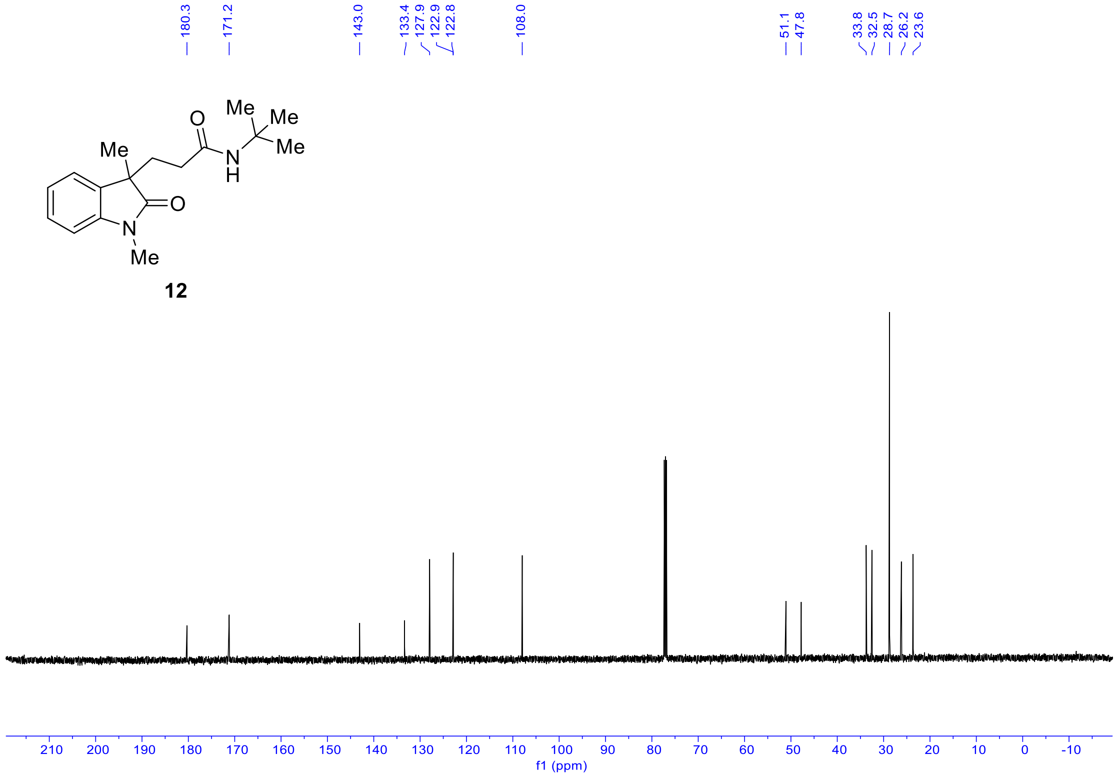

Figure S80: ^{13}C NMR of **12**, ($CDCl_3$, 126 MHz)

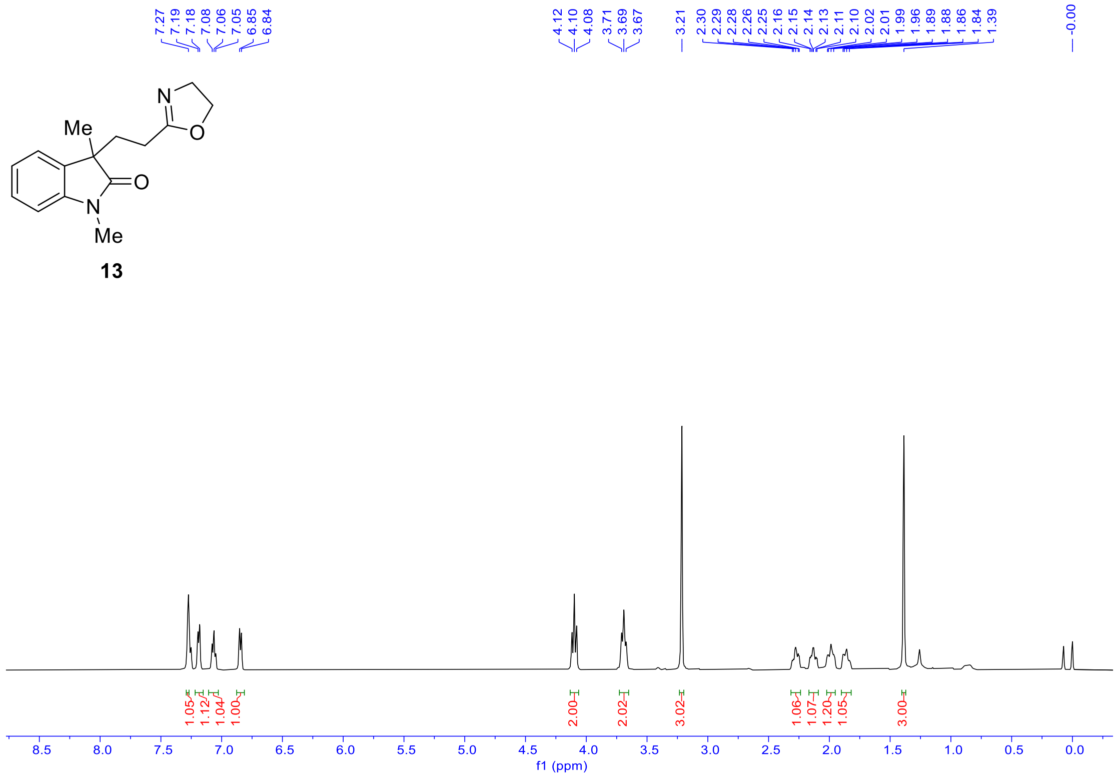

Figure S81: ^{1}H NMR of **13**, ($CDCl_3$, 500 MHz)

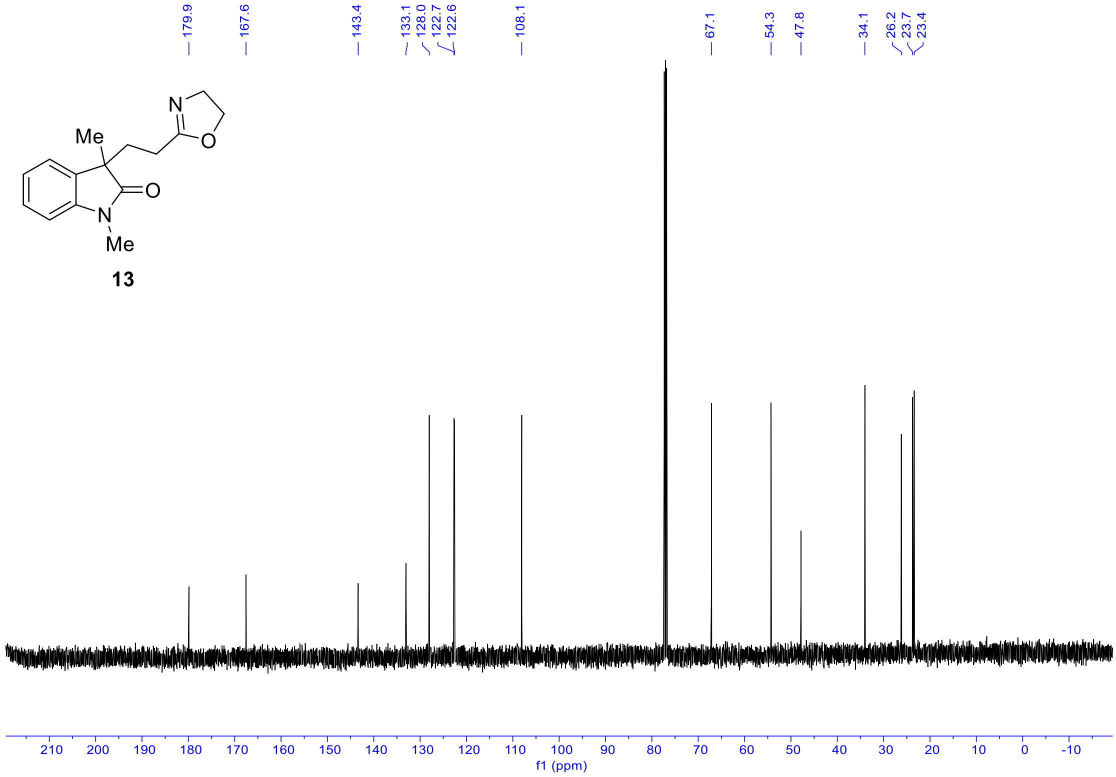

Figure S82: ^{13}C NMR of **13**, ($CDCl_3$, 126 MHz)

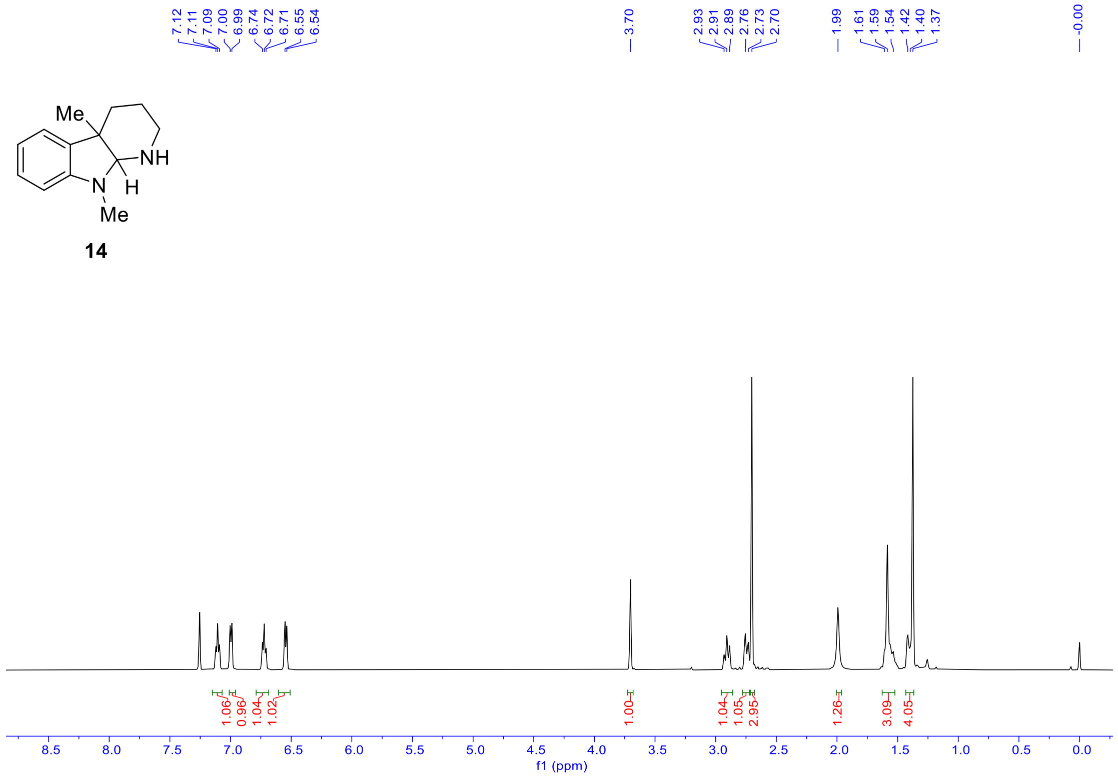

Figure S83: ^{1}H NMR of **14**, ($CDCl_3$, 500 MHz)

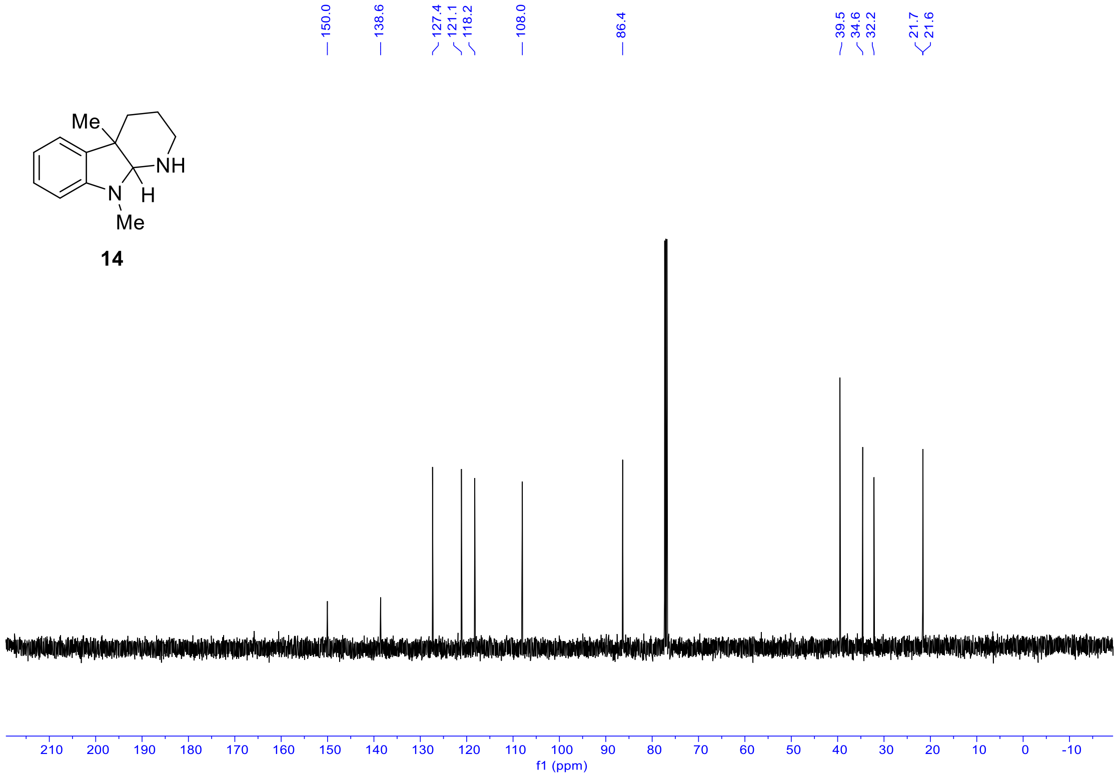

Figure S84: ^{13}C NMR of **14**, ($CDCl_3$, 126 MHz)

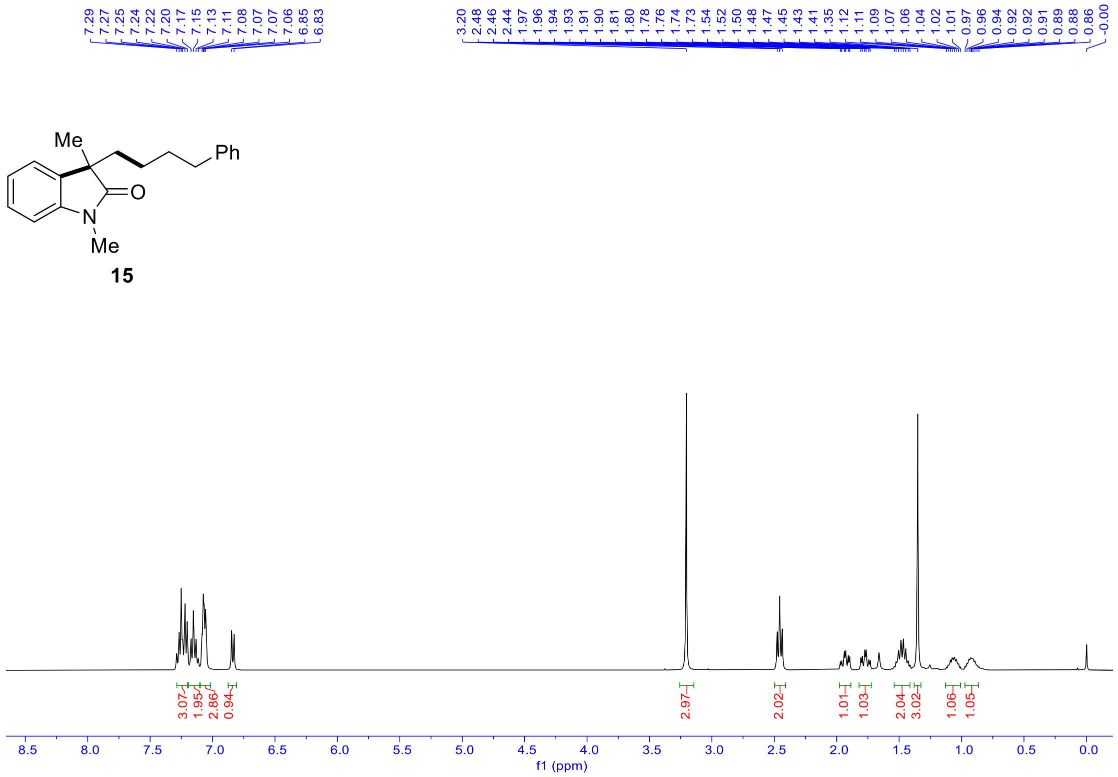

Figure S85: ^{1}H NMR of **15**, ($CDCl_3$, 500 MHz)

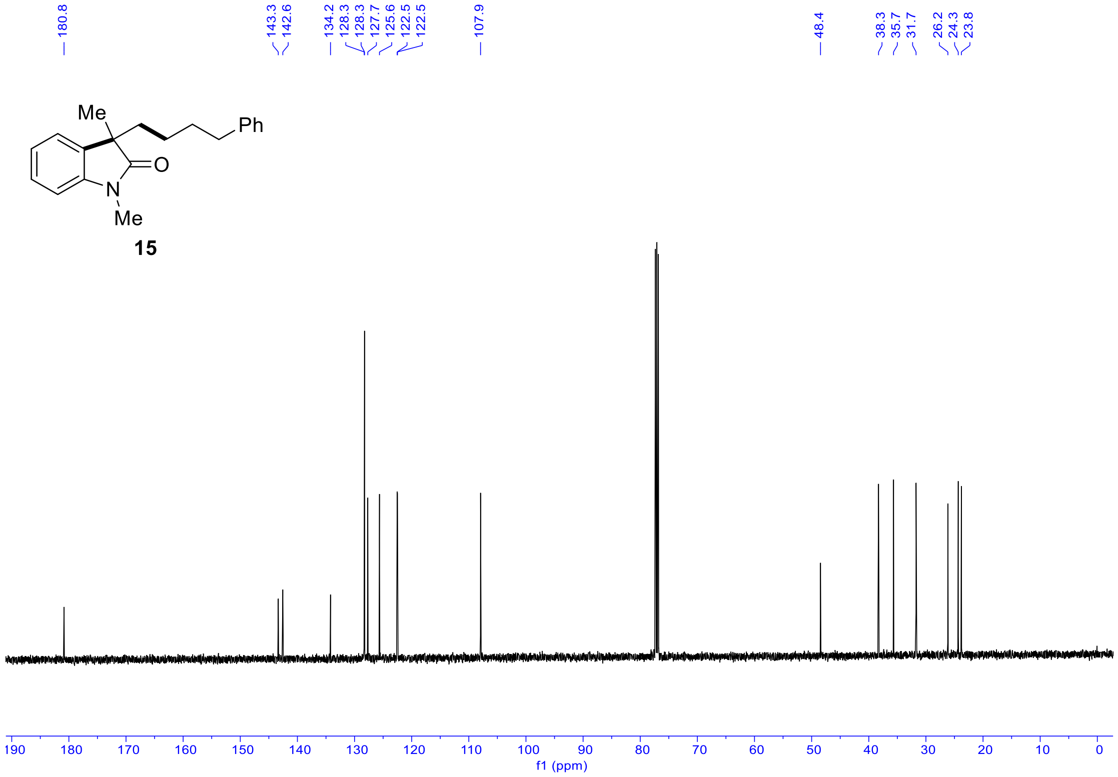

Figure S86: ^{13}C NMR of **15**, ($CDCl_3$, 126 MHz)

References

1. Liu, L.; Dong, J.; Yan, Y.; Yin, S.-F.; Han, L.-B.; Zhou, Y. *Chem. Commun.* **2019**, *55*, 233–236.
2. Zhang, H.; Chen, P.; Liu, G.; *Synlett* **2012**, *23*, 2749–2752.
3. Wu, H.; Yang, B.; Zhu, L.; Lu, R.; Li, G.; Lu, H. *Org. Lett*. **2016**, *18*, 5804–5807.
4. Jia, B.; Yang, Y.; Jin, X.; Mao, G.; Wang, C. *Org. Lett*. **2019**, *21*, 6259–6263.
5. Meca, L.; Císařová, I.; Dvořák, D. *Organometallics* **2003**, *22*, 3703–3709.
6. Li, Y.-L.; Wang, J.-B.; Wang, X.-L.; Cao, Y.; Deng, J. *Eur. J. Org. Chem.* **2017**, *2017*, 6052–6059.
7. Li, C.-C.; Yang, S.-D. *Org. Lett.* **2015**, *17*, 2142–2145.
8. Kong, W.; Casimiro, M.; Fuentes, N.; Merino, E.; Nevado, C.; *Angew. Chem., Int. Ed*. **2013**, *52*, 13086–13090.
9. Yang, Q.; Pan, G.; Wei, J.; Wang, W.; Tang, Y.; Cai, Y. *ACS Sustain. Chem. Eng.* **2021**, *9*, 2367-2377.
10. Pan, C.; Wang, Y.; Wu, C.; Yu, J. T. *Catal. Commun.* **2019**, *131*, 105802.
11. Zhang, H.; Chen, P.; Liu, G. *Synlett* **2012**, *23*, 2749–2752.
12. Li, J.; Wang, Z.; Wu, N.; Gao, G.; You, J. *Chem. Commun.* **2014**, *50*, 15049–15051.
13. Gao, X.; Dong, W.; Hu, B.; Gao, H.; Yuan, Y.; Xie, X.; Zhang, Z. *RSC Adv.* **2017**, *7*, 49299–49302.
14. Ni, Z.; Huang, X.; Wang, J.; Pan, Y. *RSC Adv.* **2016**, *6*, 522–526.
15. Lu, M.; Zhang, T.; Tan, D.; Chen, C.; Zhang, Y.; Huang, M.; Cai, S. *Adv. Synth. Catal.* **2019**, *361*, 4237–4242.
16. Wu, T.; Mu, X.; Liu, G. *Angew. Chem., Int. Ed.* **2011**, *50*, 12578–12581.
17. Pan, C.; Zhang, H.; Zhu, C. *Org. Biomol. Chem*. **2015**, *13*, 361–364.
18. Reddy, K. L. *Tetrahedron Lett.* **2003**, *44*, 1453–1455.

19. Witte, H.; Seeliger, W. *Angew. Chem., Int. Ed.* **1972**, *11*, 287–288.

20. Pinto, A.; Jia, Y.; Neuville, L.; Zhu, J. *Chem. Eur. J.* **2007**, *13*, 961–967.

21. Ding, S.; Ren, H.; Zhu, M.; Ma, Q.; Miao, Z.; Li, P. *Synth. Commun.* **2021**, *51*, 593-600.
